# Author Correction: The REGγ inhibitor NIP30 increases sensitivity to chemotherapy in p53-deficient tumor cells

**DOI:** 10.1038/s41467-020-18767-0

**Published:** 2020-09-23

**Authors:** Xiao Gao, Qingwei Wang, Ying Wang, Jiang Liu, Shuang Liu, Jian Liu, Xingli Zhou, Li Zhou, Hui Chen, Linian Pan, Jiwei Chen, Da Wang, Qing Zhang, Shihui Shen, Yu Xiao, Zhipeng Wu, Yiyun Cheng, Geng Chen, Syeda Kubra, Jun Qin, Lan Huang, Pei Zhang, Chuangui Wang, Robb E. Moses, David M. Lonard, Bert W. O’ Malley, Fuad Fares, Bianhong Zhang, Xiaotao Li, Lei Li, Jianru Xiao

**Affiliations:** 1 East China Normal University and Shanghai Changzheng Hospital Joint Research Center for Orthopedic Oncology, East China Normal University, 500 Dongchuan Road, Shanghai, 200241 China; 2 Department of Orthopedic Oncology, Changzheng Hospital, The Second Military Medical University, 415 Fengyang Road, Shanghai, 200003 China; 3 Shanghai Key Laboratory of Regulatory Biology, Institute of Biomedical Sciences, School of Life Sciences, East China Normal University, 500 Dongchuan Road, Shanghai, 200241 China; 4 Department of Surgery, Department of Physiology & Cell Biology, College of Medicine, Davis Heart and Lung Research Institute, Wexner Medical Center, The Ohio State University, Columbus, OH 43210 USA; 5 The Institute of Aging Research, School of Medicine, Hangzhou Normal University, Hangzhou, 310036 Zhejiang China; 6 Department of Hematology, Guangdong Second Provincial General Hospital, Guangzhou, Guangdong China; 7 Reproductive & Developmental Biology Laboratory, National Institute of Environmental Health Sciences (NIEHS), Research Triangle Park, NC 27709 USA; 8 The Joint Laboratory of Translational Medicine, National Center for Protein Sciences (Beijing) and Peking University Cancer Hospital, State Key Laboratory of Proteomics, Institute of Lifeomics, 102206 Beijing, China; 9 Department of Physiology and Biophysics, University of California, Irvine, CA 92697 USA; 10 Department of Pathology, The Second Chengdu Municipal Hospital, Chengdu, 610017 China; 11 Institute of Translational Medicine, Shanghai General Hospital, Shanghai Jiao Tong University School of Medicine, Shanghai, China; 12 Department of Molecular and Cellular Biology, Dan L. Duncan Cancer Center, Baylor College of Medicine, One Baylor Plaza, Houston, TX 77030 USA; 13 Department of Human Biology. Faculty of Natural Sciences, University of Haifa, Haifa, 3498838 Israel

**Keywords:** Cancer therapeutic resistance, Tumour-suppressor proteins, Cell signalling, Post-translational modifications

Correction to: *Nature Communications* 10.1038/s41467-020-17667-7, published online 06 August 2020.

The original version of this article contained an error in the spelling of the author Syeda Kubra, which was incorrectly given as Syeda Krubra. This has now been corrected in both the PDF and HTML versions of the article.

