# Supplementary material for: The REGγ inhibitor NIP30 increases sensitivity to chemotherapy in p53-deficient tumor cells
Source: Nat Commun. 2020 Aug 6;11:3904. doi: 10.1038/s41467-020-17667-7 (PMC7413384; doi:10.1038/s41467-020-17667-7)
Supplement: Supplementary file 1 — Supplementary Information [file 41467_2020_17667_MOESM1_ESM.pdf]

Supplementary Information

The REGγ inhibitor NIP30 increases sensitivity to chemotherapy in p53-deficient tumor cells

Gao et al .

Supplementary Figure Legends

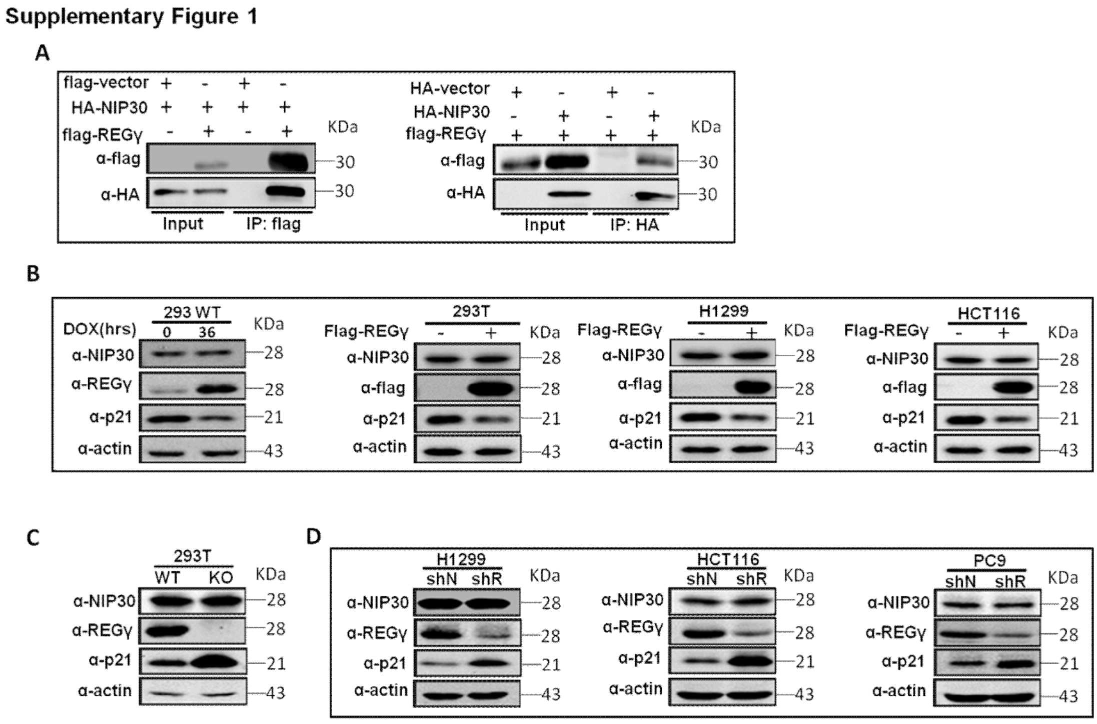

Supplementary Figure 1. NIP30 is not a substrate of REGγ. (A) Reciprocal interactions between REGγ and NIP30 in 293T cells were determined by exogenous co-immunoprecipitation and Western blot analysis in three independent experiments. Data represent mean ± SEM. (B) 293 REGγ-WT (active REGγ) cells were induced by doxycycline (DOX, 1μg mL-1) for 36 hrs; 293T, H1299 and HCT116 cells were transfected with flag-REGγ for 24hrs, then the expression of NIP30, REGγ and p21 was examined by Western blotting in three independent experiments. Data represent mean ± SEM. (C) The protein level of NIP30 and p21 were tested in REGγ WT and knock out 293T cells. Actin was used as a loading control. Data represent mean ± SEM from three independent experiments. (D) Stably expressing a control shRNA (shN) or a REGγ-specific shRNA (shR) was generated in H1299, HCT116 and PC9 cells. The total cell lysates were analyzed by immunoblotting with indicated antibodies (NIP30, Flag, p21 and actin). Data represent mean ± SEM from three independent experiments.

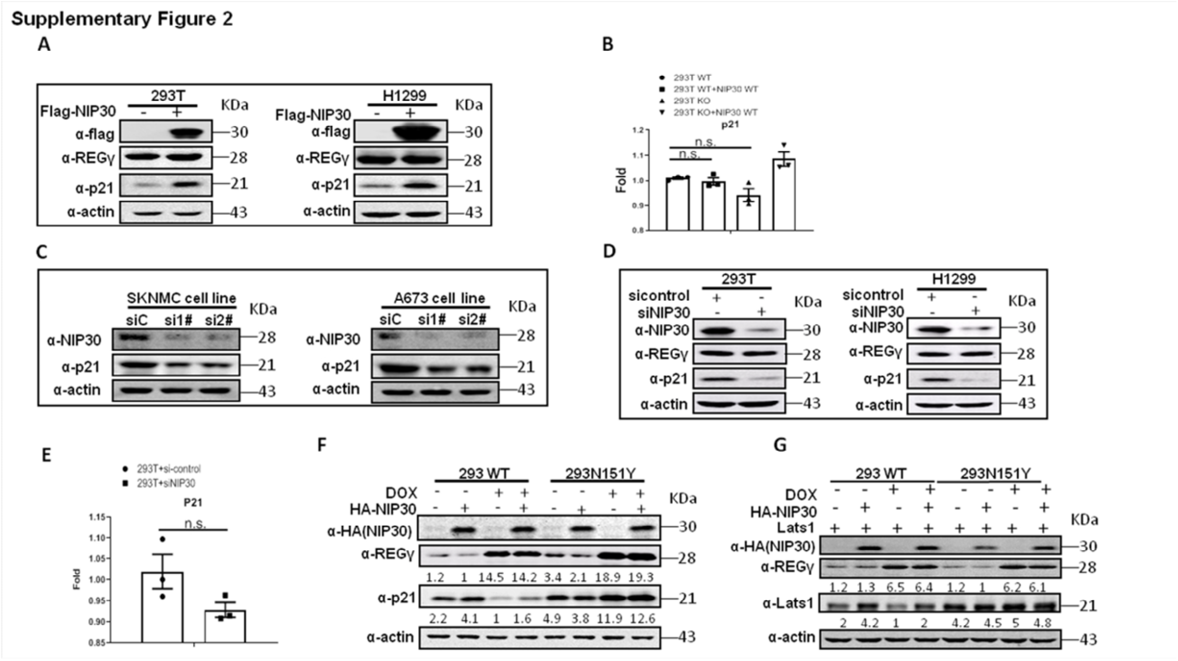

Supplementary Figure 2. NIP30 prevents the turnover of p21 protein. (A) 293T and H1299 cells were transfected with 2μg NIP30 plasmid. Cell lysates were subjected to Western blot analysis using flag, REGγ, p21 and actin in three independent experiments. Data represent mean ± SEM. (B) 293T (REGγ-WT) and REGγ-KO cells were transfected with flag-NIP30 plasmid for 24hrs. The RT-PCR assay was performed to analyze the expression of p21 PCR; n=3, each group. Data represent means±SEM. n.s.=no significance (one-way Anova). (C) SKNMC and A673 cells were transiently transfected with NIP30-siRNA or control-siRNA for 72 hrs. The protein level of NIP30 and p21 was checked by Western blot in three independent experiments. Data represent mean ± SEM. (D) 293T and H1299 cells were transiently transfected with NIP30-siRNA or control-siRNA for 72 hrs. Cell lysates were subjected to Western blot analysis using indicated antibodies in three independent experiments. Data represent mean ± SEM. (E) The mRNA expression of p21 was detected in 293T cells transfected NIP30-siRNA or control-siRNA by RT-PCR; n=3, each group. Data represent means±SEM. n.s.=no significance (two tail t-test). (F) 293 REGγ-WT (active REGγ) and 293 REGγ-N151Y (inactive REGγ) cells were induced by doxycycline (DOX, 1μg mL-1) for 36 hrs, and then transfected 1ug HA-p21, 1ug HA-vector or HA-NIP30 followed by

Western Blot analysis. Data represent mean ± SEM from three independent experiments. (G) 293 REGγ-WT (active REGγ) and 293 REGγ-N151Y (inactive REGγ) cells were induced by doxycycline (DOX, 1μg mL-1) for 36 hrs, and then transfected 1ug HA-Lats1, 1ug HA-vector or HA-NIP30 followed by Western Blot analysis. Data represent mean ± SEM from three independent experiments.

Supplementary Figure 3

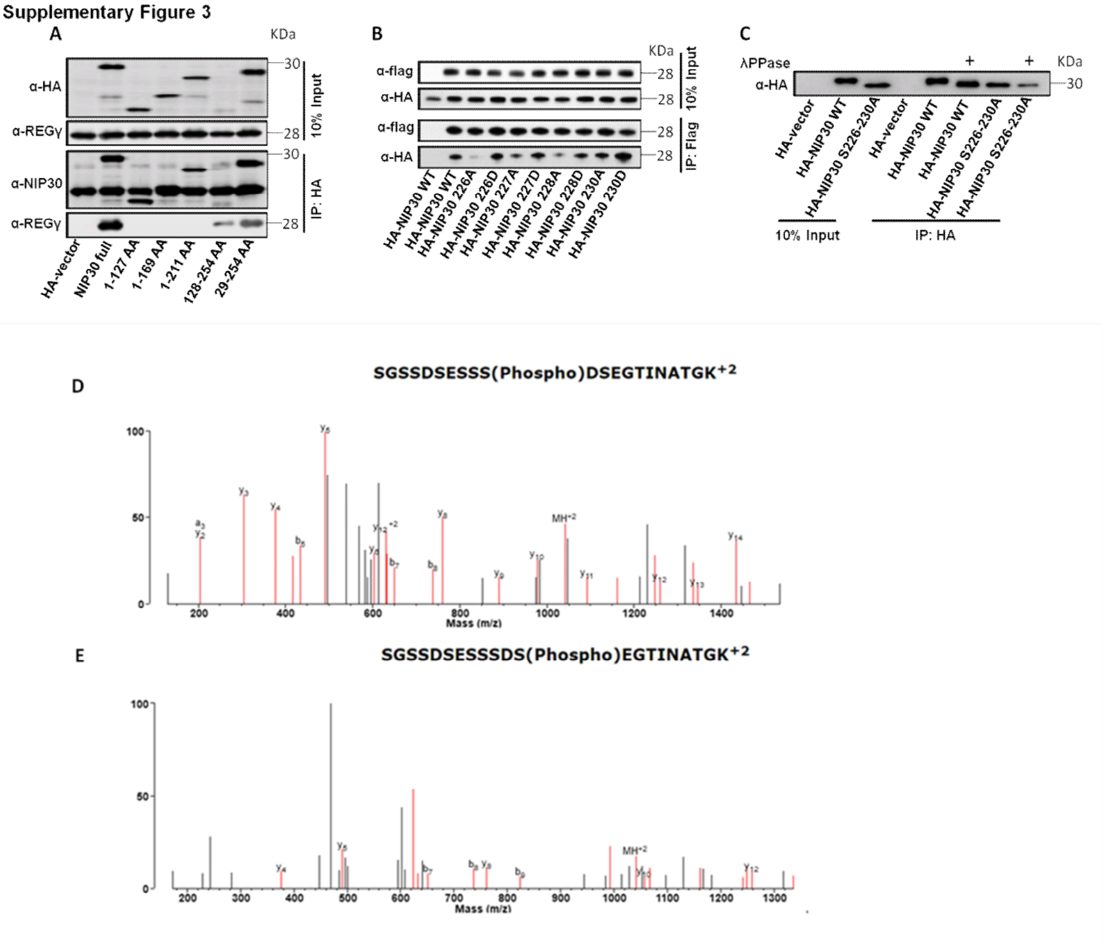

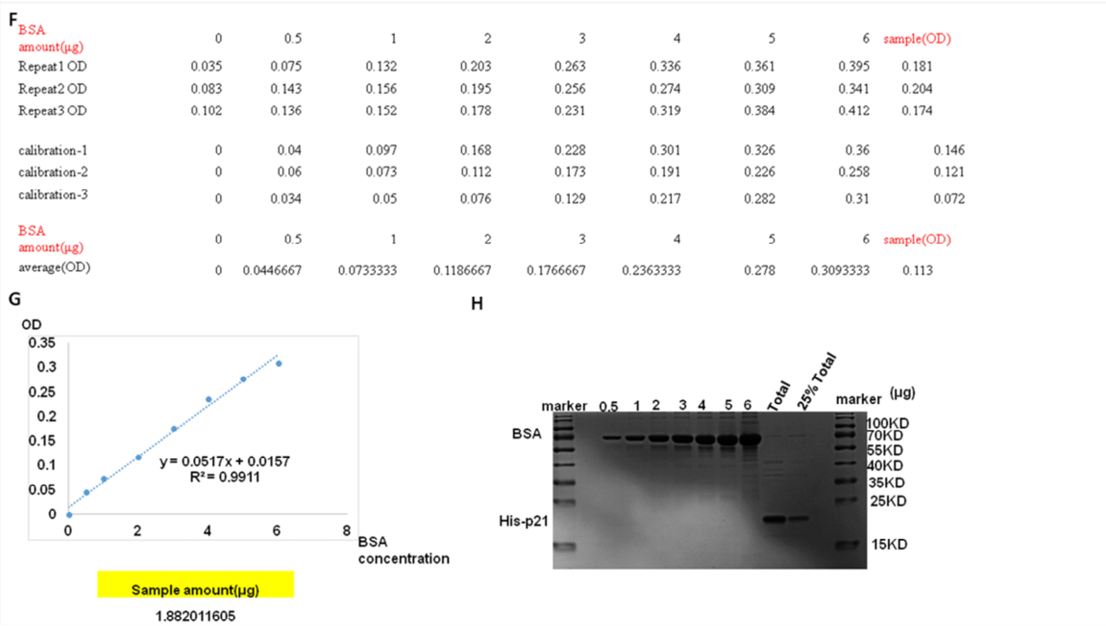

F									
BSA amount(µg)	0	0.5	1	2	3	4	5	6	sample(OD)
Repeat1 OD	0.035	0.075	0.132	0.203	0.263	0.336	0.361	0.395	0.181
Repeat2 OD	0.083	0.143	0.156	0.195	0.256	0.274	0.309	0.341	0.204
Repeat3 OD	0.102	0.136	0.152	0.178	0.231	0.319	0.384	0.412	0.174
calibration-1	0	0.04	0.097	0.168	0.228	0.301	0.326	0.36	0.146
calibration-2	0	0.06	0.073	0.112	0.173	0.191	0.226	0.258	0.121
calibration-3	0	0.034	0.05	0.076	0.129	0.217	0.282	0.31	0.072
BSA amount(µg)	0	0.5	1	2	3	4	5	6	sample(OD)
average(OD)	0	0.0446667	0.0733333	0.1186667	0.1766667	0.2363333	0.278	0.3093333	0.113

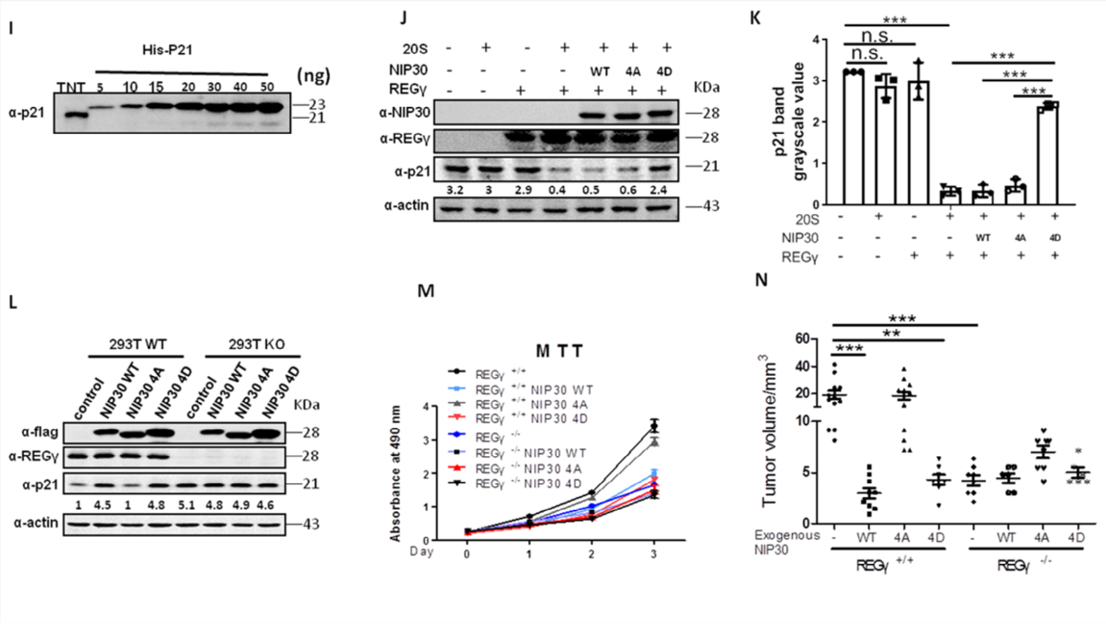

Supplementary Figure 3. Phosphorylation of NIP30 at specific sites is required for its regulation of REGγ pathway. (A) NIP30 fragments 1-127, 1-169, 1-211, 128-254, 29-254 were constructed in PSG5-HA tag vector and co-transfected with PSG5-HA vector (HA-empty), NIP30 full length and flag-REGγ in 293T cells for 48hrs. HA beads were used for immunoprecipitation assay. (B) 293T cells were transfected with 2µg HA-vector or HA-NIP30 WT or HA-NIP30 4A for 36hrs. HA beads were used for immunoprecipitation assay, then treated with λPP or dd water. Cell lysates

were analyzed by immunoblotting with anti-HA antibody. Data in this figure are representatives of three independent repeats. (C) 293T cells were transfected with flag-tagged REGγ and HA-tagged NIP30 wildtype (WT) or single point mutation of NIP30 S226A, NIP30 S226D, NIP30 S227A, NIP30 S227D, NIP30 S228A, NIP30 S228D, NIP30 S230A, NIP30 S230D plasmids. Cell lysates were immunoprecipitated with anti-Flag beads and probed with anti-HA and anti-flag antibodies. (D, E) Stable 293 cell lines expressing HBTH-hNIP30 pellets were collected and incubated with streptavidin beads. And then the conjugated protein complexes in beads were digested and identified by LC MS/MS analysis. (F-H) The His-p21 protein concentration purified by *E. coli* was measured as 1.88μg/μl based on a standard curve with commercially available BSA (0.5μg/μl, 1μg/μl, 2μg/μl, 3μg/μl, 4μg/μl, 5μg/μl, 6μg/μl) using a BCA kit for the absorbance at 562 nm. A Coomassie brilliant blue staining result was shown to demonstrate different concentrations of BSA standards and the purified His-p21 proteins. (I) Following application of 1 microgram of p21 plasmid in a 50 microliter TNT system for *in vitro* translation, the amount of p21 in a 5 microliter TNT translation system was estimated as 10~15 ng by Western blot analysis of 5 microliter of TNT mix along with 5ng, 10ng, 15ng, 20ng, 30ng, 40ng, and 50ng His-p21. (J) Purified NIP30 WT, NIP30 4A, NIP30 4D, REGγ, 20S proteasome and *in vitro* translated p21 proteins were incubated at 30℃ as described in Materials and Methods. Anti-actin immunoblot is shown as the loading controls. Grayscale values of p21 were measured by Image J. The numbers below p21 bands represent values normalized against loading controls (actin) relative to the grayscale value in lane 1 which is set as a reference for the rest of bands. (K) The quantitated p21 values in J were statistically analyzed along with additional two repeating degradation experiments (see Source Data P 41/42). To ensure reliable comparison, the grayscale values of the first p21 bands in the repeating experiments were arbitrarily set to 3.2 (same as in J) followed by normalization of relative p21 levels for the rest of lanes. Values in each bar are

presented as the means ± SEM. ***, $p<0.001$. n.s.=no significance (one-way Anova). P (lane1, lane4) = 9.6E-7, P (lane4, lane7) = 1.4E-4, P (lane5, lane7) = 1.9E-4，P (lane6, lane7) = 3.3E-4. (L) REGγ WT and knock out 293T cells were infected with plvx-EF1α-ires-puro-flag-NIP30 (WT, 4A, 4D) to construct stable cells. Cell lysates were collected and flag, REGγ, p21 and actin were analyzed by Western blotting. (M) The same quantity of 293T cells as were seeded, cell proliferation was analyzed by MTT assay. Data represented mean + SEM (n=4) (N) Xenograft Tumors were dissected and volumes were measured; n=12, REGγ+/+ group; n=9, REGγ+/+NIP30 WT group; n=12, REGγ+/+NIP30 4A group; n=8, REGγ+/+NIP30 4D group; n=8, REGγ-/- group; n=6, REGγ-/-NIP30 WT group; n=8, REGγ-/-NIP30 4A group; n=6, REGγ-/-NIP30 4D group. Values are presented as the means ± SEM. *, $p<0.05$; **, $p<0.01$ (one-way Anova). P(lane1, lane2) = 3.4E-4, P(lane1, lane4) = 0.002, P(lane1, lane4) = 9.7E-4.

Supplementary Figure 4

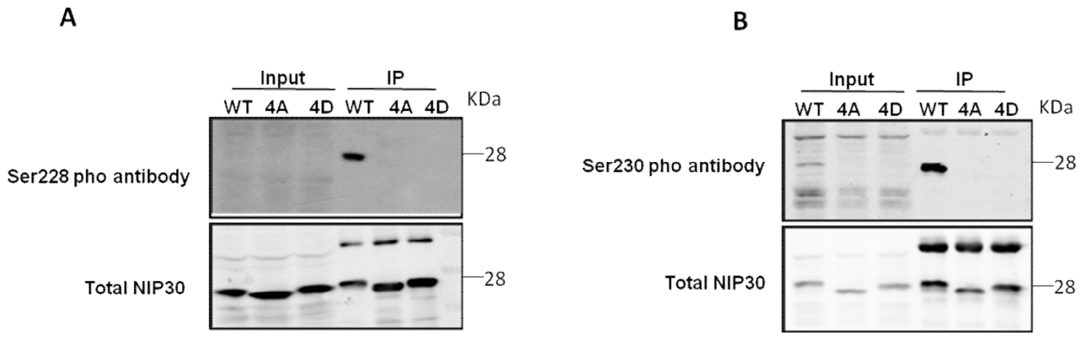

Supplementary Figure 4. Validation of the specificity of antibodies for pNIP30-Ser228 and pNIP30-Ser230. (A) 293T cells were transfected with HA-NIP30 WT, HA-NIP30 4A, HA-NIP30 4D for 48 hrs, and then HA beads were used to pull down the protein by anti-HA. pNIP30-Ser228 and total NIP30 antibody were used in Western blot. (B) 293T cells were transfected with HA-NIP30 WT, HA-NIP30 4A, HA-NIP30 4D for 48 hrs, and then HA beads were used to pull down the protein by HA-antibody. –pNIP30-Ser230 and total

NIP30 antibody were used in Western blot. All data in this figure are representatives of three independent repeats.

Supplementary Figure 5

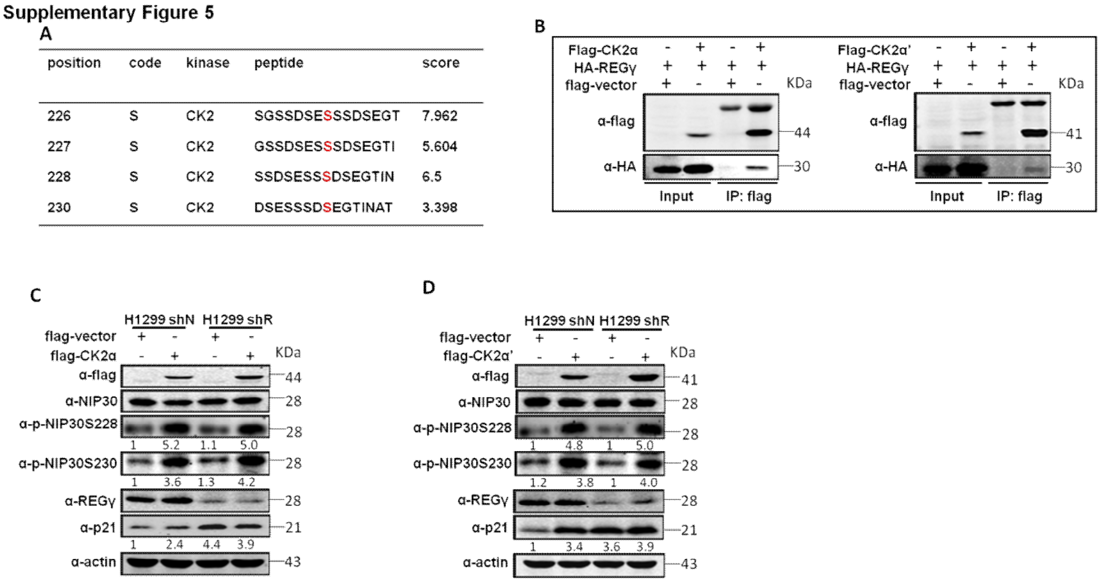

A

position	code	kinase	peptide	score
226	S	CK2	SGSSDSESSSSDSEGT	7.962
227	S	CK2	GSSDSESSSSDSEGTI	5.604
228	S	CK2	SSDSESSSSDSEGTIN	6.5
230	S	CK2	DSESSSDSEGTINAT	3.398

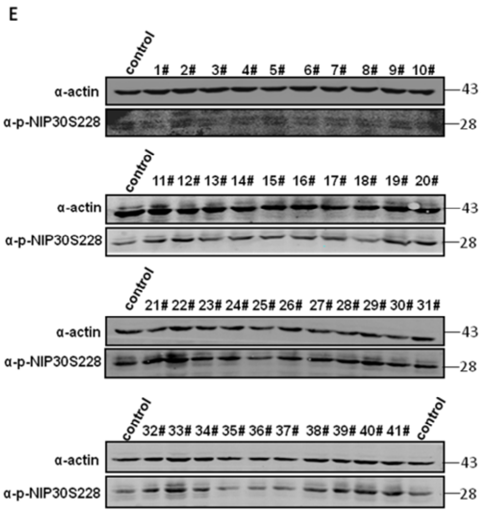

F

```
   1 ggtgaactat agaatagggc cctctagatg catgctcgag tcagagcttc ttcagacgac
  61 tgtacatctc cctcttgctc ttctcccctg cccaggtccg gctcttggtg cggaacttct
 121 tcaggtcttc tttaaagtcc tcgtggtgca tgggccggta gctagggggc tcacagtaag
 181 actggcattt cataaagaac tccttgtatc ccccttcag gacatacagc tcagggtagt
 241 ggagtttggg gtattcatta cccaggcgat ctctctctct cacataccgg cacatgcggg
 301 gacctctctc agaagaaaac tcgcagtgaa acacaacaat gacacgcttg ccatcagtag
 361 gtacaatggg cttcttcaat aagaagtctt caacctcttc ttccatgtgc aagttcactg
 421 caccccttgat gtggcctccc tcgtattcat atgggtatcg acagtcgatg ataacaaact
 481 ctttaatgag gttggcaaac ttgccattca aaacagatgc cataatttct ggagagatgt
 541 attttaaatc ctgatgtttc ccagcaactg tatgaaagag ataacccttg gagaagtctc
 601 ctataaggtc ccttgggtca ttgtccaaaa tgttctcaat ggttcctttg ggggaagatg
 661 ccagggataa agactgatga agagtctcat gggccttctc tggattagtt gactctttgg
 721 ggctggcccc agacatgctc ttcctcctct ttgtacttcc aggtggagac tcctcttgag
 781 atcgttctgg tctcttcaac actgaccgag tgctggagct acacagggaa ggggagtcaa
 841 acagcttgc atcggttgtc aaggtttgta gttctcatga cgagaggagc tgtccagaggc
 901 ttgccatgc acgagggggt ctcctcctca ttcttcagat tctctccatc gagaaggtcca
 961 cgaagccat catcctcatc agacaaagtg gctgtcacag gtgactgggg tgtaaaaagag
1021 gaatgaaat tccctggttc actgctatct ctttcatttg aggaaagcat ccgagctgggg
1081 cagagttct gcctctgtgt gaagagatct ttaccctcct ggagtccatg agagtgcaggc
1141 agccacgag atacaggtct tactggcttc ttaaactcaa aggcttcatt ttccttgttct
1201 catctgggt cgatgagctg aaagatgtca tggtcaagag aatcagaatg gctcctcttca
1261 gagctggac tacatcccaa cagcttctga ggtagggaat gtattcttct catagg attt
1321 caaggtttt ctttactgtc caatggccca ggagaatcta gacagaaacc tgaatctgttg
1381 actcggagg agcccattct ctgcagatta ctgttgttct tcacctccag tggttgctcat
1441 aatcactgc ccagaccctg cagctggtcc atagtgacgg tcaggttggt gacaggcgaca
1501 gtcccccgg cggctgaagc gccaaatagc gccttcacga cgggctgcga cgcgggagggg
1561 ggctgcagg cgaagagcag gcggcggcgg tgcgggggct ccgggcccag ttccatggtgg
1621 cggatccct tgtcatcatc gtccttgtag tctcccatgg taagcttggg tctccctatag
1681 tgagtcgta ttaatttcga taagccagta agcagtgggt tctctagtta gccagagagct
1741 ctgct
```

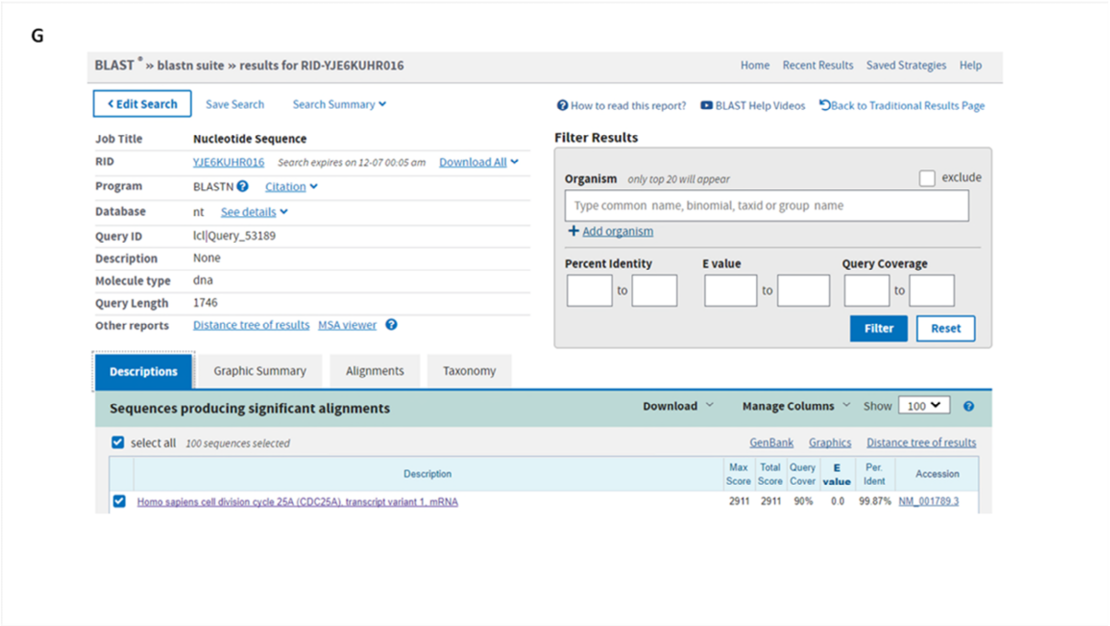

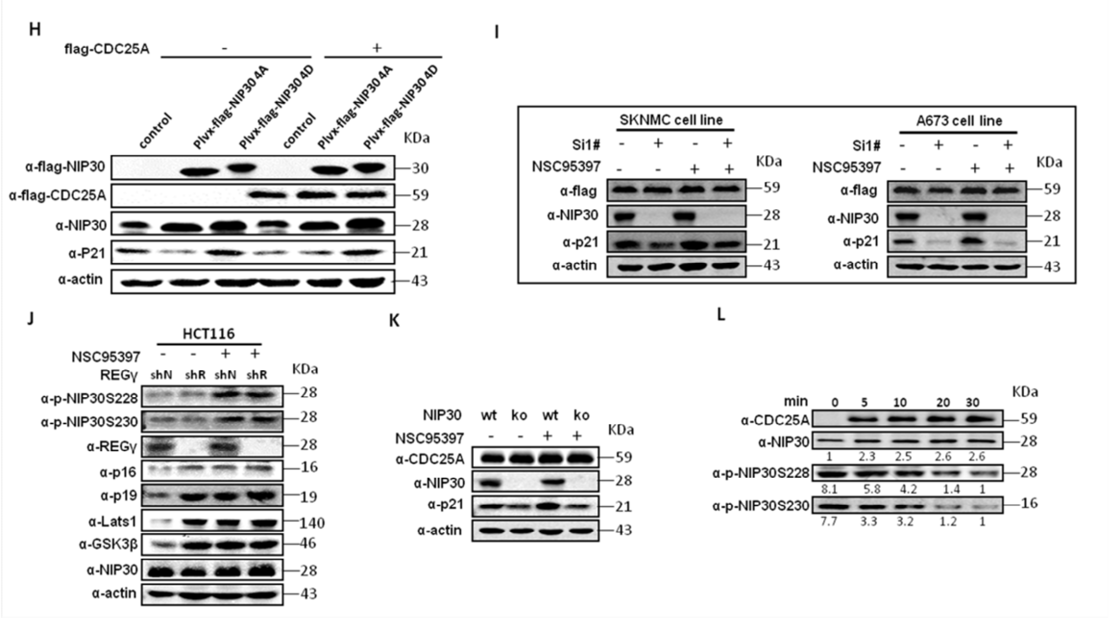

Supplementary Figure 5. CK2 is an upstream kinase of NIP30. (A) Prediction of the upstream kinase using GPS2.1. (B) Reciprocal interactions between CK2 and NIP30 in 293T cells were determined by exogenous co-immunoprecipitation and Western blot analysis. Data represent mean ± SEM from three independent experiments. (C) Overexpressed Flag-CK2α in H1299 cells. Cell lysates were subjected to Western blot analysis using flag, REGγ, total NIP30, pNIP30Ser228, pNIP30Ser230, p21 and actin. Data represent mean ± SEM from three independent experiments. (D) Overexpressed Flag-CK2α' in H1299 cells. Cell lysates were subjected to

Western blot analysis using flag, REGγ, total NIP30, pNIP30Ser228, pNIP30Ser230, p21 and actin. Data represent mean ± SEM from three independent experiments. (E) In 293T cells, 2ug candidate phosphatase plasmids (indicated by numbers for double-blinded screening) or pcDNA3.0-vector (control) were transiently transfected for 36hrs and then the expression of p-NIP30S228 was examined by Western blotting. Data represent mean ± SEM from three independent experiments. (F) The sequencing results of the selected candidate. (G) Sequencing results were obtained through NCBI database. (H) 293T WT and stable overexpression of NIP30 4A/ 4D cells were transfection with flag-CDC25A. Flag-CDC25A, p21 and actin were analyzed by Western blotting in three independent experiments. Data represent mean ± SEM. (I) NIP30 was knocked down in SKNMC and A673 cells after treatment with NSC95397 for 3 hrs. Cell lysates were subjected to Western blot analysis. Data represent mean ± SEM from three independent experiments. (J) HCT116 (shN) and shR cells were treated with NSC95397 for 6 hrs. Cell lysates were subjected to Western blot analysis. Data represent mean ± SEM from three independent experiments. (K) Primary MEF cells were isolated from NIP30 Wild type and knock out mice and then NSC95397 was treated in cells for 6 hrs. The cell lysates were collected and the protein level of CDC25A, actin, p21 and NIP30 were analyzed by Western blotting in three independent experiments. Data represent mean ± SEM. (L) Flag-NIP30 and flag-CDC25A were individually expressed in 293T cells. Cell lysates were immunoprecipitated with anti-flag M2 agarose beads. The purified NIP30 was incubated with or without CDC25A for 5, 10, 20, or 30 minutes. The reaction products were separated by SDS-PAGE and analyzed by Western blot. Data represent mean ± SEM from three independent experiments.

Supplementary Figure 6

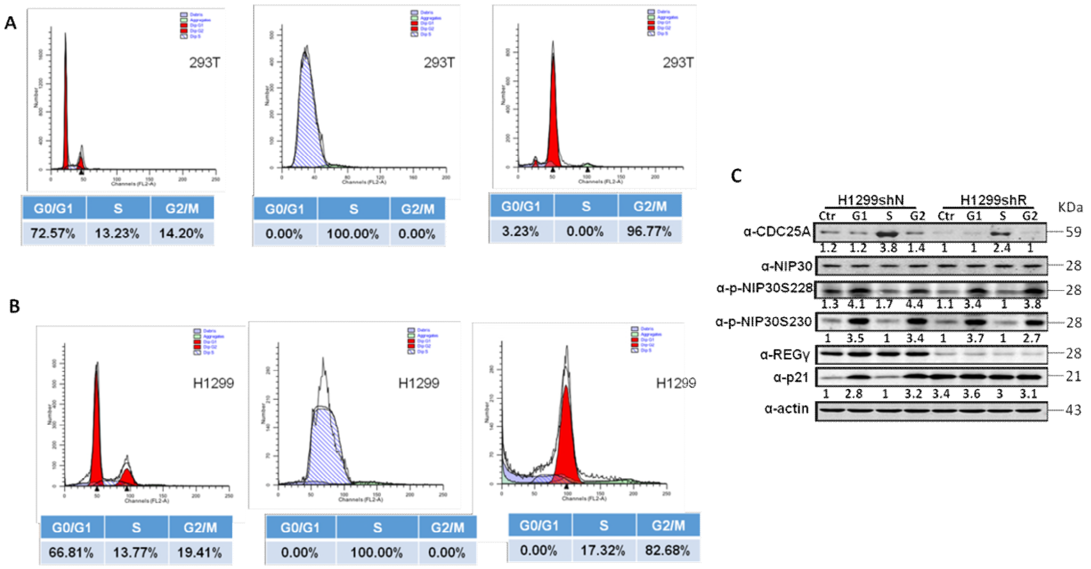

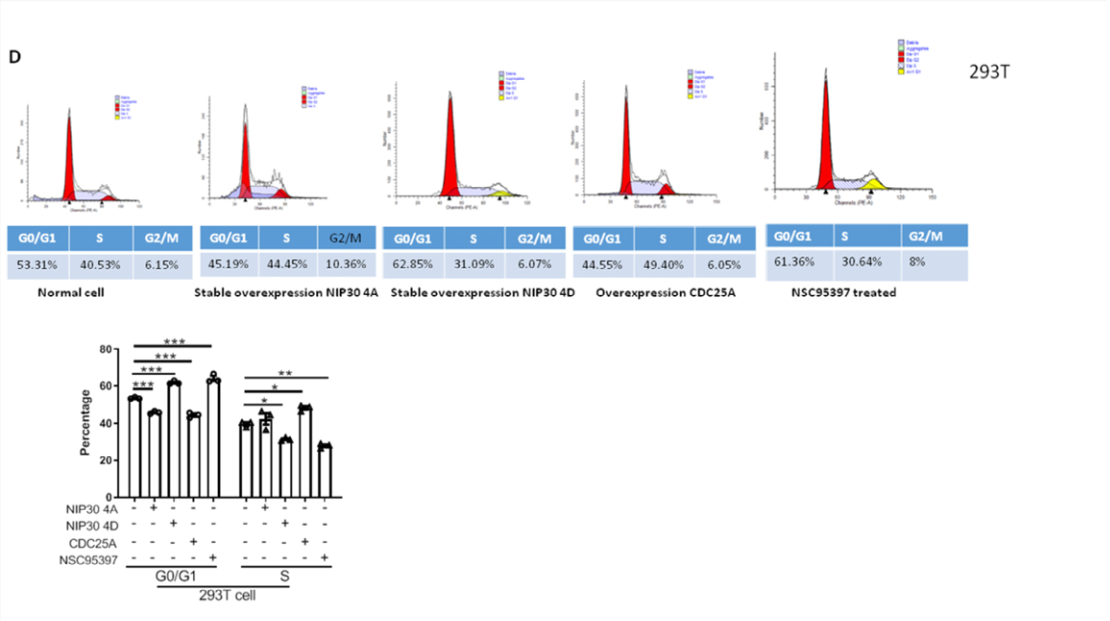

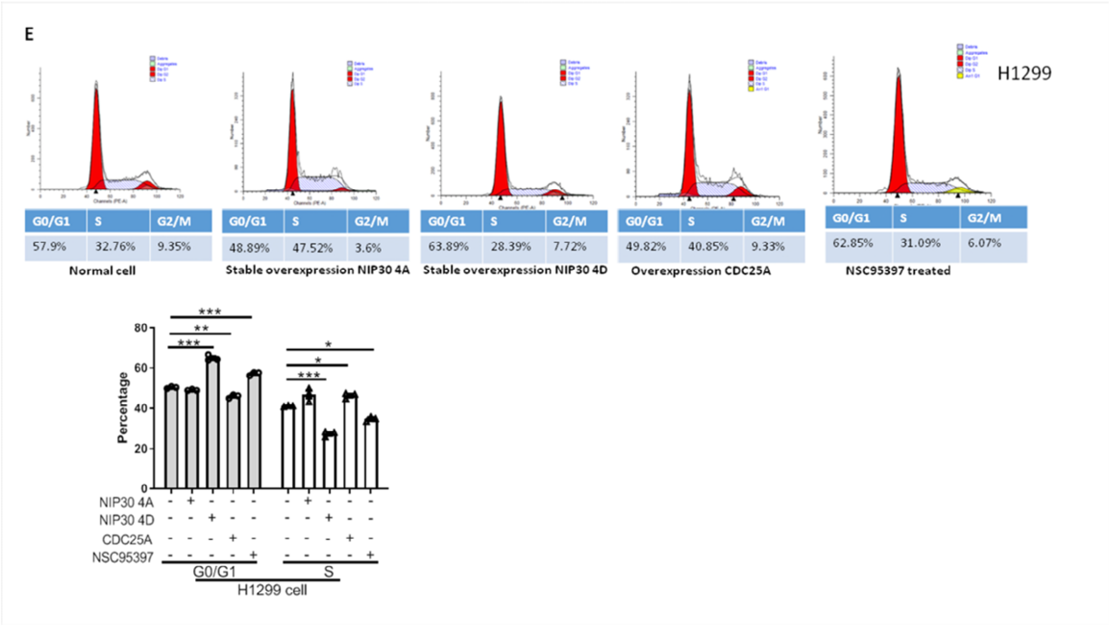

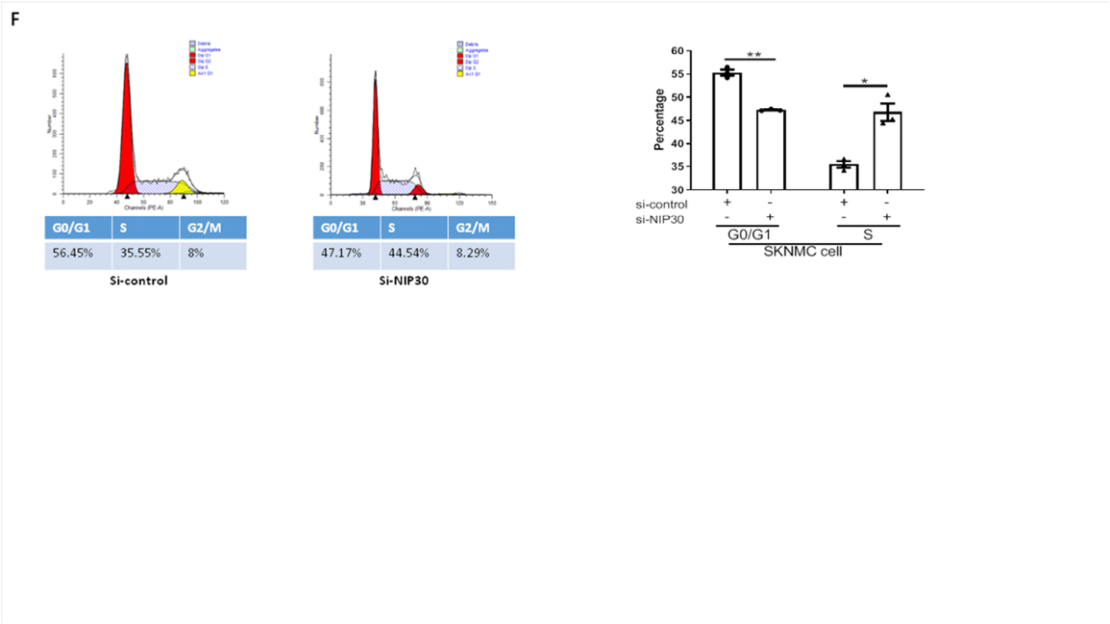

Supplementary Figure 6. CDC25A-NIP30-REGγpathway activated in cell cycle from G0/G1 to S phase. (A) REGγ WT and knock out 293T cells were stained with PI and analyzed by flow cytometry analysis to enure synchronized to G0/G1, S, G2/M phase. (B) H1299 REGγ shN and shR cells were stained with PI and analyzed by flow cytometry analysis to make sure synchronized to G0/G1, S, G2/M phase. (C) Synchronized H1299 REGγ shN and shR cells to G0/G1, S, G2/M phase, followed by Western blot analysis using indicated antibodies. Data represent mean ± SEM from three independent experiments. (D) DNA content was analyzed by flow cytometry analysis in Normal, stable

NIP30 4A/4D overexpressed, CDC25A overexpressed and NSC95397 treated 293T cells. Data represented mean ± SEM (n=3, one-way Anova). *, p<0.05; **, p<0.01; ***, p<0.001. P(lane1, lane2)=2.6E-4, P(lane1, lane3)=1.6E-4, P(lane1, lane4)=6.3E-5, P(lane1, lane5)=2E-5, P(lane6, lane8)=0.032, P(lane6, lane9)=0.027, P(lane6, lane10)=0.003. (E) DNA content was analyzed by flow cytometry analysis in Normal, stable NIP30 4A/4D overexpressed, CDC25A overexpressed and NSC95397 treated H1299 cells. Data represented mean ± SEM (n=3, one-way Anova). *, p<0.05; **, p<0.01; ***, p<0.001. P(lane1, lane3) =2.1E-8, P(lane1, lane4) =1.6E-3, P(lane1, lane5) =2.5E-5, P(lane6, lane8) =3.3E-5, P(lane6, lane9) =0.046, P(lane6, lane10) =0.015. (F) DNA content was analyzed by flow cytometry analysis in SKNMC cells treated with si-Control or siRNA. Data represented mean ± SEM (n=3, two-tailed t-test). *, p<0.05; **, p<0.01. P(lane1, lane2) =4.1E-3, P(lane3, lane4) =0.048.

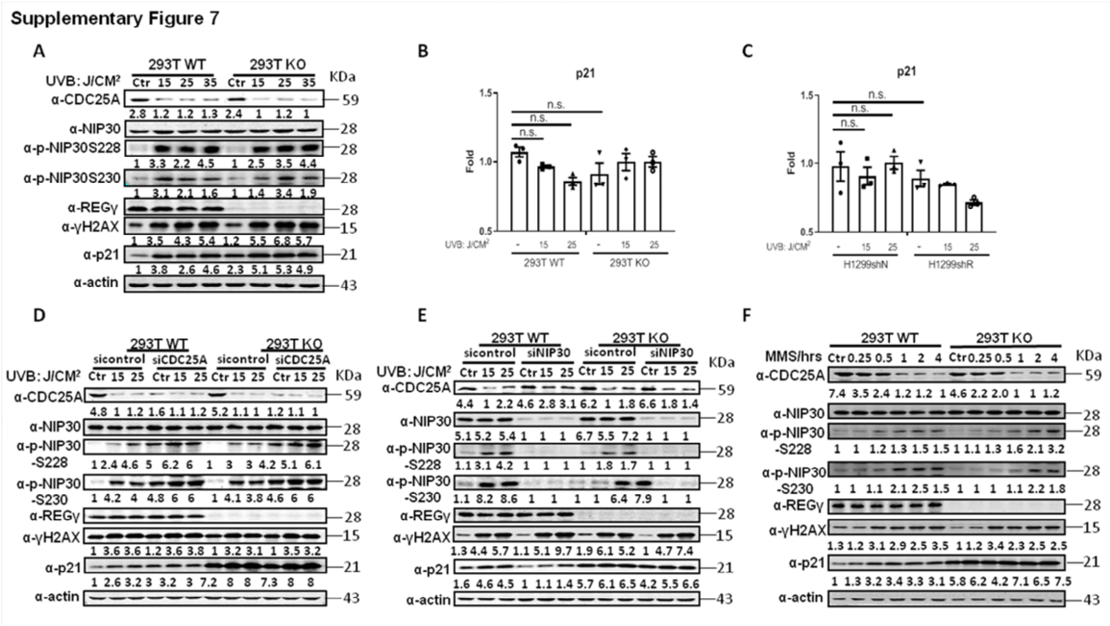

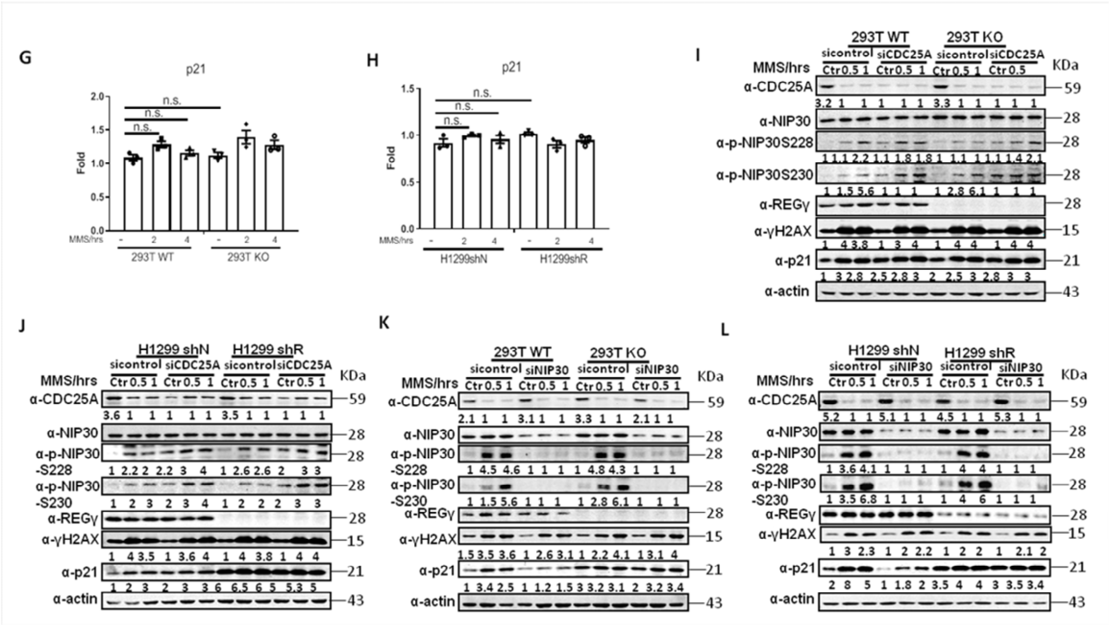

Supplementary Figure 7. The action of the CDC25A-NIP30-REGγ pathway following DNA Damage. (A) REGγ WT and knock out 293T cells were treated with 0, 15 J/cm^2, 25 J/cm^2 or 35 J/cm^2 UV irradiation and harvested 12hrs later. Cell lysates were immunoprecipitated with indicated antibodies. Data represent mean ± SEM from three independent experiments. (B) REGγ WT and knock out 293T cells were treated with 0, 15 J/cm^2, 25 J/cm^2 UV irradiation and harvested 12hrs later. The real time PCR assay was performed to analyze p21 gene expression; n=3, each group. Values are presented as the means ± SEM. n.s.=no significance (one-way Anova) (C) H1299 REGγ shN and shR cells were treated with 0, 15 J/cm^2, 25 J/cm^2 UV irradiation and harvested 12hrs later. The real time PCR assay was performed to analyze p21 gene expression; n=3, each group. Values are presented as the means ± SEM. n.s.=no significance (one-way Anova) (D) REGγ WT and knock out 293T cells were transiently transfected with siCDC25A/Control , then treated with 15 J/cm^2, 25 J/cm^2 UV irradiation and harvested for 12hrs. Cell lysates were subjected to Western blot using indicated antibodies. Data represent mean ± SEM from three independent experiments. (E) REGγ WT and knock out 293T cells were transiently transfected with siNIP30/Control, then treated with 15 Jcm^2, 25 J/cm^2 UV irradiation and harvested for 12hrs. Cell lysates were

immunoprecipitated with indicated antibodies. Data represent mean ± SEM from three independent experiments. (F) REGγ WT and knock out 293T cells were treated with 0.2mM MMS for different time. The levels of CDC25A, total NIP30, $NIP30^{Ser228p}$, $NIP30^{Ser230p}$, p21, REGγ and γH2AX were determined by immunoblotting. Actin was used as a loading control. Data represent mean ± SEM from three independent experiments. (G) REGγ WT and knock out 293T cells were treated with 0.2mM MMS for different time and real time PCR assay was performed to analysis p21 gene expression; n=3, each group. Values are presented as the means ± SEM. n.s.=no significance (one-way Anova) (H) H1299 REGγ shN and shR cells were treated with 0.2mM MMS for different time and real time PCR assay was performed to analysis p21 gene expression; n=3, each group. Values are presented as the means ± SEM. n.s.=no significance (one-way Anova) (I) REGγ WT and knock out 293T cells that knock down CDC25A by siRNA were treated with 0.2mM MMS for 0.5 hr or 1hr. Cell lysates were subjected to Western blot using indicated antibodies. Data represent mean ± SEM from three independent experiments. (J) H1299 REGγ shN and shR cells were transiently transfected with siCDC25A/Control, and then cells were treated with 0.2mM MMS for 0.5 hr or 1hr. Cell lysates were subjected to Western blot using indicated antibodies in three independent experiments. Data represent mean ± SEM. (K) REGγ WT and knock out 293T cells that knock down NIP30 by siRNA were treated with 0.2mM MMS for 0.5 hr or 1hr. Cell lysates were subjected to Western blot using indicated antibodies. Data represent mean ± SEM from three independent experiments. (L) H1299 REGγ shN and shR cells cells were transiently transfected with siNIP30/Control and then cells were treated with 0.2mM MMS for 0.5 hr or 1hr. Cell lysates were subjected to Western blot using indicated antibodies. Data represent mean ± SEM from three independent experiments.

Supplementary Figure 8

A

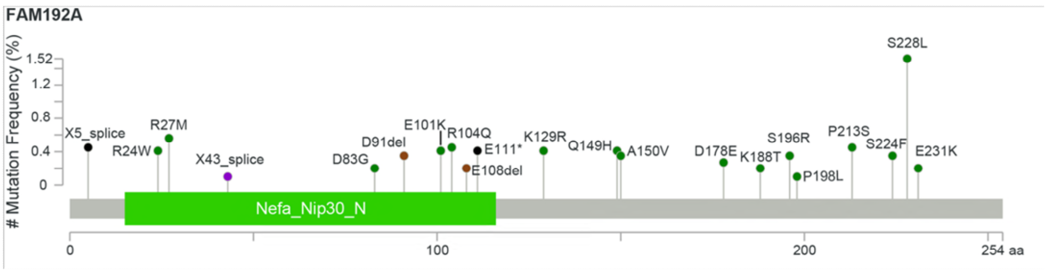

Supplementary Figure 8. GCTA analysis of NIP30. (A) Distribution of point mutations in the NIP30 gene.

Supplementary Table 1 NIP30 mutation analysis from TCGA database

Study	Protein Change	Mutation Type	Chromosome	Start Pos	End Pos	Ref	Var	Mut cases	All samples	Mutant frequency
Kidney Chromophobe (TCGA, Provisional)	S228L	Missense_Mutation	16	57188284	57188284	G	A	1	66	1.515%
Lung Squamous Cell Carcinoma (TCGA, Provisional)	R27M	Missense_Mutation	16	57207687	57207687	C	A	1	178	0.562%
Colorectal Adenocarcinoma (TCGA, Provisional)	P213S	Missense_Mutation	16	57188330	57188330	G	A	1	220	0.455%
Colorectal Adenocarcinoma (TCGA, Provisional)	R104Q	Missense_Mutation	16	57206200	57206200	C	T	1	220	0.455%
Kidney Renal Clear Cell Carcinoma (TCGA, Provisional)	X5_splice	Splice_Site	16	57207783	57207783	T	A	2	448	0.446%
Uterine Corpus Endometrial Carcinoma (TCGA, Provisional)	E111*	Nonsense_Mutation	16	57206180	57206180	C	A	1	242	0.413%
Uterine Corpus Endometrial Carcinoma (TCGA, Provisional)	E101K	Missense_Mutation	16	57206210	57206210	C	T	1	242	0.413%
Uterine Corpus Endometrial Carcinoma (TCGA, Provisional)	R24W	Missense_Mutation	16	57207697	57207697	G	A	1	242	0.413%
Uterine Corpus Endometrial Carcinoma (TCGA, Provisional)	Q149H	Missense_Mutation	16	57201040	57201040	C	A	1	242	0.413%
Uterine Corpus Endometrial Carcinoma (TCGA, Provisional)	K129R	Missense_Mutation	16	57201101	57201101	T	C	1	242	0.413%
Skin Cutaneous Melanoma (TCGA, Provisional)	S224F	Missense_Mutation	16	57188296	57188296	G	A	1	287	0.348%
Skin Cutaneous Melanoma (TCGA, Provisional)	D91del	In_Frame_Del	16	57206236	57206238	TCA	-	1	287	0.348%
Stomach Adenocarcinoma (TCGA, Provisional)	S196R	Missense_Mutation	16	57188381	57188381	T	G	1	287	0.348%
Stomach Adenocarcinoma (TCGA, Provisional)	A150V	Missense_Mutation	16	57201038	57201038	G	A	1	287	0.348%
Liver Hepatocellular Carcinoma (TCGA, Provisional)	D178E	Missense_Mutation	16	57197926	57197926	A	T	1	366	0.273%
Prostate Adenocarcinoma (TCGA, Provisional)	D83G	Missense_Mutation	16	57206263	57206263	T	C	1	492	0.203%
Head and Neck Squamous Cell Carcinoma (TCGA, Provisional)	K188T	Missense_Mutation	16	57188404	57188404	T	G	1	504	0.198%
Head and Neck Squamous Cell Carcinoma (TCGA, Provisional)	E108del	In_Frame_Del	16	57206187	57206189	TTC	-	1	504	0.198%
Head and Neck Squamous Cell Carcinoma (TCGA, Provisional)	E231K	Missense_Mutation	16	57188276	57188276	C	T	1	504	0.198%
Breast Invasive Carcinoma (TCGA, Provisional)	P198L	Missense_Mutation	16	57188374	57188374	G	A	1	963	0.104%
Breast Invasive Carcinoma (TCGA, Provisional)	X43_splice	Splice_Region	16	57206793	57206793	G	A	1	963	0.104%

Supplementary Table 2 NIP30 deletion analysis from TCGA database

	Deletion cases	All cases	Frequency
Bladder Urothelial Carcinoma (TCGA, Provisional)	13	408	3.19%
Prostate Adenocarcinoma (TCGA, Provisional)	14	492	2.85%
Lymphoid Neoplasm Diffuse Large B-cell Lymphoma (TCGA, Provisional)	1	48	2.08%
Uveal Melanoma (TCGA, Provisional)	1	80	1.25%
Skin Cutaneous Melanoma (TCGA, Provisional)	3	367	0.82%
Thymoma (TCGA, Provisional)	1	123	0.81%
Stomach Adenocarcinoma (TCGA, Provisional)	3	441	0.68%
Esophageal Carcinoma (TCGA, Provisional)	1	184	0.54%
Acute Myeloid Leukemia (TCGA, Provisional)	1	191	0.52%
Sarcoma (TCGA, Provisional)	1	257	0.39%
Cervical Squamous Cell Carcinoma and Endocervical Adenocarcinoma (TCGA, Provisional)	1	295	0.34%
Liver Hepatocellular Carcinoma (TCGA, Provisional)	1	370	0.27%
Lung Adenocarcinoma (TCGA, Provisional)	1	516	0.19%
Head and Neck Squamous Cell Carcinoma (TCGA, Provisional)	1	522	0.19%
Uterine Corpus Endometrial Carcinoma (TCGA, Provisional)	1	539	0.19%
Glioblastoma Multiforme (TCGA, Provisional)	1	577	0.17%
Ovarian Serous Cystadenocarcinoma (TCGA, Provisional)	1	579	0.17%
Breast Invasive Carcinoma (TCGA, Provisional)	1	1080	0.09%

Supplementary Table 3 Phosphorylated at S228

$b-H_3PO_4$	b				y	y^{+2}	$y-H_3PO_4$	$y-H_3PO_4^{+2}$
---	---	1	S	21	---	---	---	---
---	145.0608	2	G	20	1995.7663	998.3868	1897.7894	949.3983
---	232.0928	3	S	19	1938.7448	969.876	1840.7679	920.8876
---	319.1248	4	S	18	1851.7128	926.36	1753.7359	877.3716
---	434.1518	5	D	17	1764.6807	882.844	1666.7038	833.8556
---	521.1838	6	S	16	1649.6538	825.3305	1551.6769	776.3421
---	650.2264	7	E	15	1562.6218	781.8145	1464.645	732.8261
---	737.2584	8	S	14	1433.579	717.2932	1335.602	668.3048
---	824.2904	9	S	13	1346.547	673.7772	1248.57	624.7888
893.3119	991.2888	10	S(Phospho)	12	1259.515	630.2612	1161.538	581.2727
1008.339	1106.316	11	D	11	1092.517	546.762	---	---
1095.371	1193.348	12	S	10	977.4898	489.2485	---	---
1224.414	1322.39	13	E	9	890.4578	445.7325	---	---
1281.435	1379.412	14	G	8	761.4152	381.2112	---	---
1382.483	1480.46	15	T	7	704.3937	352.7005	---	---
1495.567	1593.5436	16	I	6	603.3461	302.1767	---	---
1609.6096	1707.5865	17	N	5	490.262	245.6346	---	---
1680.6467	1778.6236	18	A	4	376.2191	188.6132	---	---
1781.6944	1879.6713	19	T	3	305.1819	153.0946	---	---
1838.7159	1936.6928	20	G	2	204.1343	102.5708	---	---
---	---	21	K	1	147.1128	74.06	---	---

Supplementary Table 4 Phosphorylated at S230

$b-H_3PO_4$	b				y	y^{+2}	$y-H_3PO_4$	$y-H_3PO_4^{+2}$
---	---	1	S	21	---	---	---	---
---	145.0608	2	G	20	1995.7663	998.3868	1897.7894	949.3983
---	232.0928	3	S	19	1938.7448	969.876	1840.7679	920.8876
---	319.1248	4	S	18	1851.7128	926.36	1753.7359	877.3716
---	434.1518	5	D	17	1764.6807	882.844	1666.7038	833.8556
---	521.1838	6	S	16	1649.6538	825.3305	1551.6769	776.3421
---	650.2264	7	E	15	1562.6218	781.8145	1464.6449	732.8261
---	737.2584	8	S	14	1433.5792	717.2932	1335.602	668.3048
---	824.2904	9	S	13	1346.5471	673.7772	1248.57	624.7888
---	911.3225	10	S	12	1259.515	630.2612	1161.538	581.2727
---	1026.349	11	D	11	1172.483	586.7452	1074.506	537.7567
1095.371	1193.348	12	S(Phospho)	10	1057.456	529.2317	959.4793	480.2433
1224.414	1322.39	13	E	9	890.4578	445.7325	---	---
1281.435	1379.4118	14	G	8	761.4152	381.2112	---	---
1382.4826	1480.4595	15	T	7	704.3937	352.7005	---	---
1495.5667	1593.5436	16	I	6	603.3461	302.1767	---	---
1609.6096	1707.5865	17	N	5	490.262	245.6346	---	---
1680.6467	1778.6236	18	A	4	376.2191	188.6132	---	---
1781.6944	1879.6713	19	T	3	305.1819	153.0946	---	---
1838.7159	1936.6928	20	G	2	204.1343	102.5708	---	---
---	---	21	K	1	147.1128	74.06	---	---
