# Supplementary material for: The REGγ inhibitor NIP30 increases sensitivity to chemotherapy in p53-deficient tumor cells
Source: Nat Commun. 2020 Aug 6;11:3904. doi: 10.1038/s41467-020-17667-7 (PMC7413384; doi:10.1038/s41467-020-17667-7)
Supplement: Supplementary file 3 — Reporting Summary [file 41467_2020_17667_MOESM3_ESM.pdf]

nature research

Corresponding author(s): Xiaotao Li

Last updated by author(s): Apr 18, 2020

Reporting Summary

Nature Research wishes to improve the reproducibility of the work that we publish. This form provides structure for consistency and transparency in reporting. For further information on Nature Research policies, see Authors & Referees and the Editorial Policy Checklist .

Statistics

For all statistical analyses, confirm that the following items are present in the figure legend, table legend, main text, or Methods section.

n/a	Confirmed	
☐	☒	The exact sample size (*n*) for each experimental group/condition, given as a discrete number and unit of measurement
☐	☒	A statement on whether measurements were taken from distinct samples or whether the same sample was measured repeatedly
☐	☒	The statistical test(s) used AND whether they are one- or two-sided<br>*Only common tests should be described solely by name; describe more complex techniques in the Methods section.*
☒	☐	A description of all covariates tested
☒	☐	A description of any assumptions or corrections, such as tests of normality and adjustment for multiple comparisons
☐	☒	A full description of the statistical parameters including central tendency (e.g. means) or other basic estimates (e.g. regression coefficient) AND variation (e.g. standard deviation) or associated estimates of uncertainty (e.g. confidence intervals)
☐	☒	For null hypothesis testing, the test statistic (e.g. *F*, *t*, *r*) with confidence intervals, effect sizes, degrees of freedom and *P* value noted<br>*Give P values as exact values whenever suitable.*
☒	☐	For Bayesian analysis, information on the choice of priors and Markov chain Monte Carlo settings
☒	☐	For hierarchical and complex designs, identification of the appropriate level for tests and full reporting of outcomes
☒	☐	Estimates of effect sizes (e.g. Cohen's *d*, Pearson's *r*), indicating how they were calculated

Our web collection on statistics for biologists contains articles on many of the points above.

Software and code

Policy information about availability of computer code

Data collection	LI-COR Odyssey Infrared Imaging System.
Data analysis	Image Studio Ver 5.2;Prism software (GraphPad 5.0 Software) ;One Way Analysis of Variance (ANOVA)

For manuscripts utilizing custom algorithms or software that are central to the research but not yet described in published literature, software must be made available to editors/reviewers. We strongly encourage code deposition in a community repository (e.g. GitHub). See the Nature Research guidelines for submitting code & software for further information.

Data

Policy information about availability of data

All manuscripts must include a data availability statement. This statement should provide the following information, where applicable:

- Accession codes, unique identifiers, or web links for publicly available datasets
- A list of figures that have associated raw data
- A description of any restrictions on data availability

No datasets were generated or analysed during the current study. Other raw data are available on request.

Field-specific reporting

Please select the one below that is the best fit for your research. If you are not sure, read the appropriate sections before making your selection.

☒ Life sciences ☐ Behavioural & social sciences ☐ Ecological, evolutionary & environmental sciences

For a reference copy of the document with all sections, see nature.com/documents/nr-reporting-summary-flat.pdf

Life sciences study design

All studies must disclose on these points even when the disclosure is negative.

Sample size	Group size was determined based on the results of preliminary experiments and referenced "REGgamma is critical for skin conservancies by modulating the Wnt-beta-catenin pathway. Lei Li et at. Nature Communications. 2015." No statistical method was used to predetermine sample size in animal studies.
Data exclusions	No data was excluded.
Replication	All the results haven been biologically repeated three times or more.
Randomization	Yes, all the animal were randomly to be grouped in all the experiments.
Blinding	The group design and outcome analysis were not performed in a blinded manner, because this is a routine chemistry experiment which is hard to expected. All these experiments have been recorded daily in the lab notebook and all the raw data have been securely stored in the lab.

Reporting for specific materials, systems and methods

We require information from authors about some types of materials, experimental systems and methods used in many studies. Here, indicate whether each material, system or method listed is relevant to your study. If you are not sure if a list item applies to your research, read the appropriate section before selecting a response.

Materials & experimental systems

n/a	Involved in the study	
☐	☒	Antibodies
☐	☒	Eukaryotic cell lines
☒	☐	Palaeontology
☐	☒	Animals and other organisms
☒	☐	Human research participants
☒	☐	Clinical data

Methods

n/a	Involved in the study	
☒	☐	ChIP-seq
☐	☒	Flow cytometry
☒	☐	MRI-based neuroimaging

Antibodies

Antibodies used	Anti-Flag-mouse MBL (anti-DDDDK-mouse) Monoclonal Code No. M185-3LL<br>β-actin-mouse MBL LOT:003 Monoclonal Code No. M177-3<br>anti-HA-mouse Abmart Monoclonal Code No. M20003<br>anti-REGγ-rabbit Abmart Custom antibody<br>anti-NIP30-rabbit Proteintech Rabbit Polyclonal catalog number 16830-1-AP<br>anti-p21-rabbit Proteintech Rabbit Polyclonal catalog number 10355-1-AP<br>anti-CDC25A-rabbit Proteintech Rabbit Polyclonal catalog number 55031-1-AP<br>anti-LATS1 -rabbit CST Rabbit Polyclonal catalog number 3477S<br>pNIP30Ser228 HuaBIO Custom antibody<br>pNIP30Ser230 HuaBIO Custom antibody
Validation	All the antibodies can be validated in the published study and also in our Western blot assays and summarized in the supplemental materials"antibody reporting" .

Eukaryotic cell lines

Policy information about cell lines

Cell line source(s)	HEK293T, H1299, HCT116, PC9, A673,
Authentication	All cell lines were purchased from ATCC.
Mycoplasma contamination	It is negative for mycoplasma, which was verified by PCR.
Commonly misidentified lines<br>(See ICLAC register)	No commonly misidentified cell lines were used in the study.

Animals and other organisms

Policy information about studies involving animals; ARRIVE guidelines recommended for reporting animal research

Laboratory animals	C57BL/6 genetic background
Wild animals	No wild animals were used in the study.
Field-collected samples	No field collected samples were used in the study.
Ethics oversight	Animals were maintained according to the ethical and scientific standards of the Animal Center at East China Normal University.

Note that full information on the approval of the study protocol must also be provided in the manuscript.

Flow Cytometry

Plots

Confirm that:

- [x] The axis labels state the marker and fluorochrome used (e.g. CD4-FITC).
- [x] The axis scales are clearly visible. Include numbers along axes only for bottom left plot of group (a 'group' is an analysis of identical markers).
- [x] All plots are contour plots with outliers or pseudocolor plots.
- [x] A numerical value for number of cells or percentage (with statistics) is provided.

Methodology

Sample preparation	Trypsinize and harvest cells, fix cells into 0.5ml 70% EtOH (pre-cooled to -20°C overnight), store fixed cells on ice at least 24hrs. Spin down cells for 2min at 4,000 rpm. Resuspend cell pellet in 0.5 ml PBS containg 0.25% Triton X-100 and incubate on ice for 15 min. Spin down the cells for 2min at 4,000 rpm. Discard supernatant and resuspend cell pellet in 0.5 ml PBS containg 10 ug/ml RNase A and 20 ug/ml PI(Propidium iodide) stock solution, transfer to FACS tubes and incubate at room temperature (RT) in the dark for 30 min. Ready for FACS.
Instrument	BD LSRFortessa
Software	ModFit LT3.2
Cell population abundance	The instrument counts 10,000 cells autonomously.
Gating strategy	First plot gating (FSC-A/SSC-H) for live cells , then second plot (FSC-A/FSC-H) for single and then only for PI positive cells.

- [x] Tick this box to confirm that a figure exemplifying the gating strategy is provided in the Supplementary Information.
