# Supplementary material for: The REGγ inhibitor NIP30 increases sensitivity to chemotherapy in p53-deficient tumor cells
Source: Nat Commun. 2020 Aug 6;11:3904. doi: 10.1038/s41467-020-17667-7 (PMC7413384; doi:10.1038/s41467-020-17667-7)
Supplement: Supplementary file 4 — Source Data [file 41467_2020_17667_MOESM4_ESM.pdf]

Description of Source Data

The source data underlying Figs 1b—f, 2a, 2d—g, 3a—f,4a—e,5a—f ,6a—d and Supplementary Tables and Supplementary Figs 3F-K and from antibody datasheet /FACS gating strategy are provided as a Source Data file.

Page number 2,11,32
Description: They are original pictures.
Related to Figure 1B/2G/6C

Page number 3-9/12-28
Description: They are original WB gel images.
Related to Figure 1C-1F/Fig.2D,2E/Fig 3/Fig.4/Fig.5

Page number 10,29-31,33-37
Descrition : They are original data.
Related to Figure 2F/Fig.6A,Fig.6B/Fig 6D/ Supplementary Figure 3D,3E/ Supplementary Figure 8

Page number 38-43
Descrition : They are original WB gel images and raw data.
Related Supplementary Figure3F-K

Page number44-53
Descrition : They are antibody reporting.

Page number 54-57
Descrition : They are FACS gating strategy
Related Supplementary Figure 6

Uncropped gel images for Figure 1B

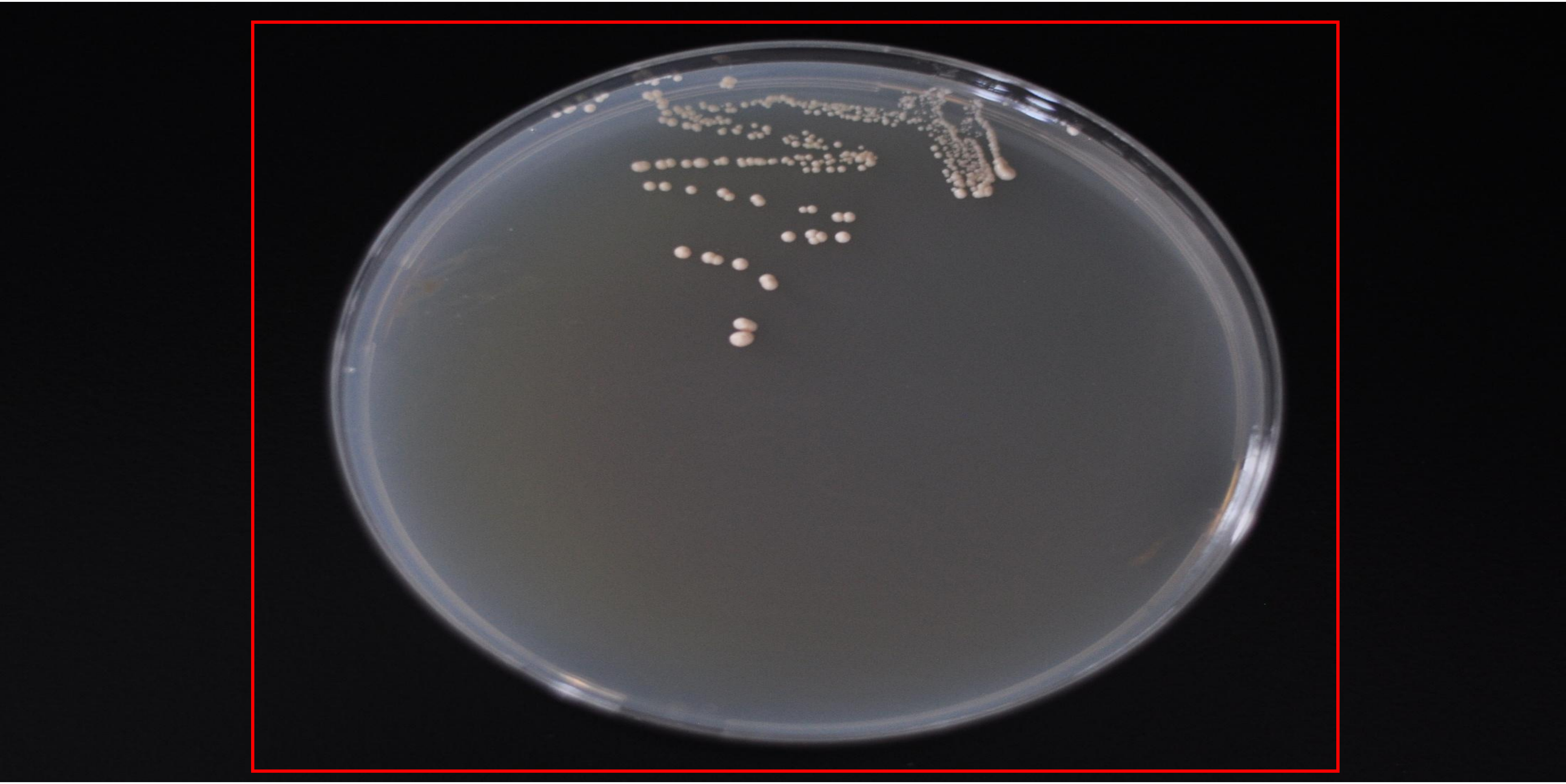

Uncropped gel images for Figure 1C

α-NIP30

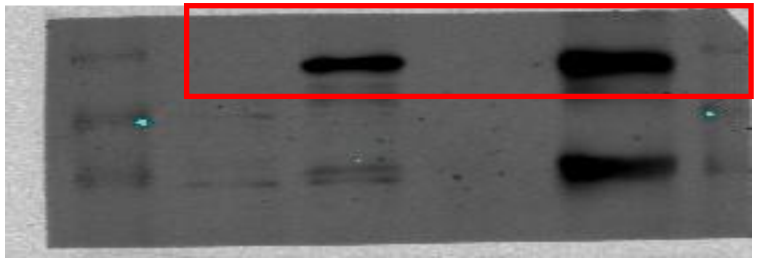

α-REGγ

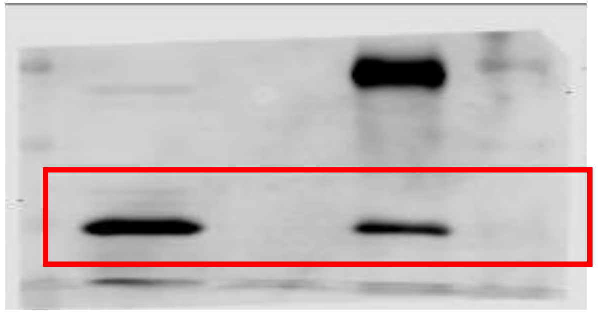

α-NIP30

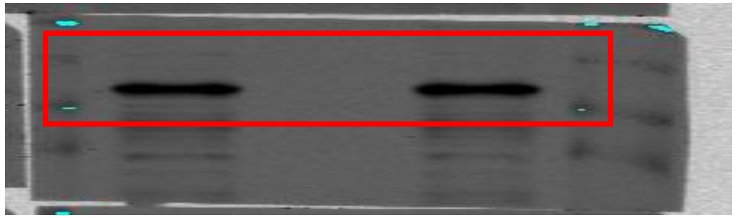

α-REGγ

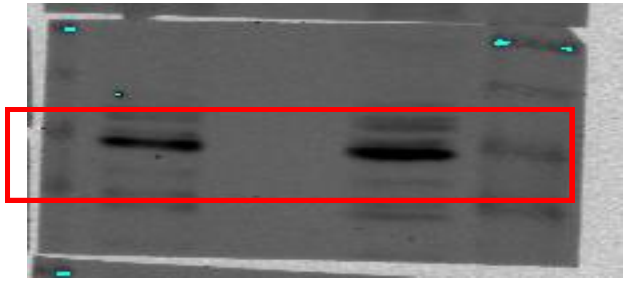

Uncropped gel images for Figure 1D

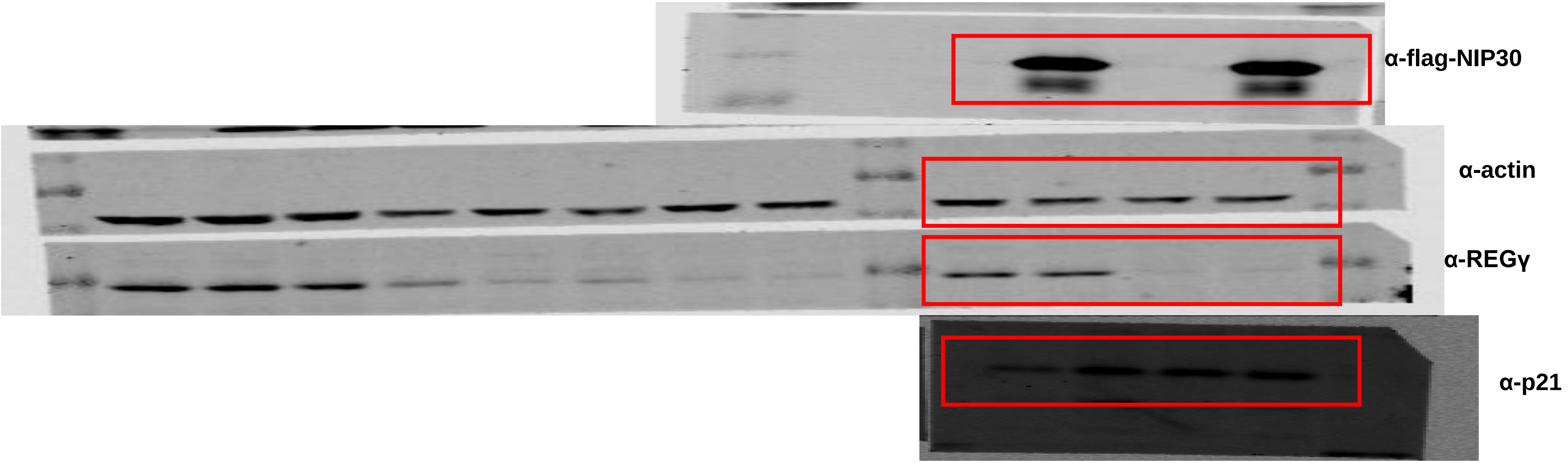

Uncropped gel images for Figure 1E

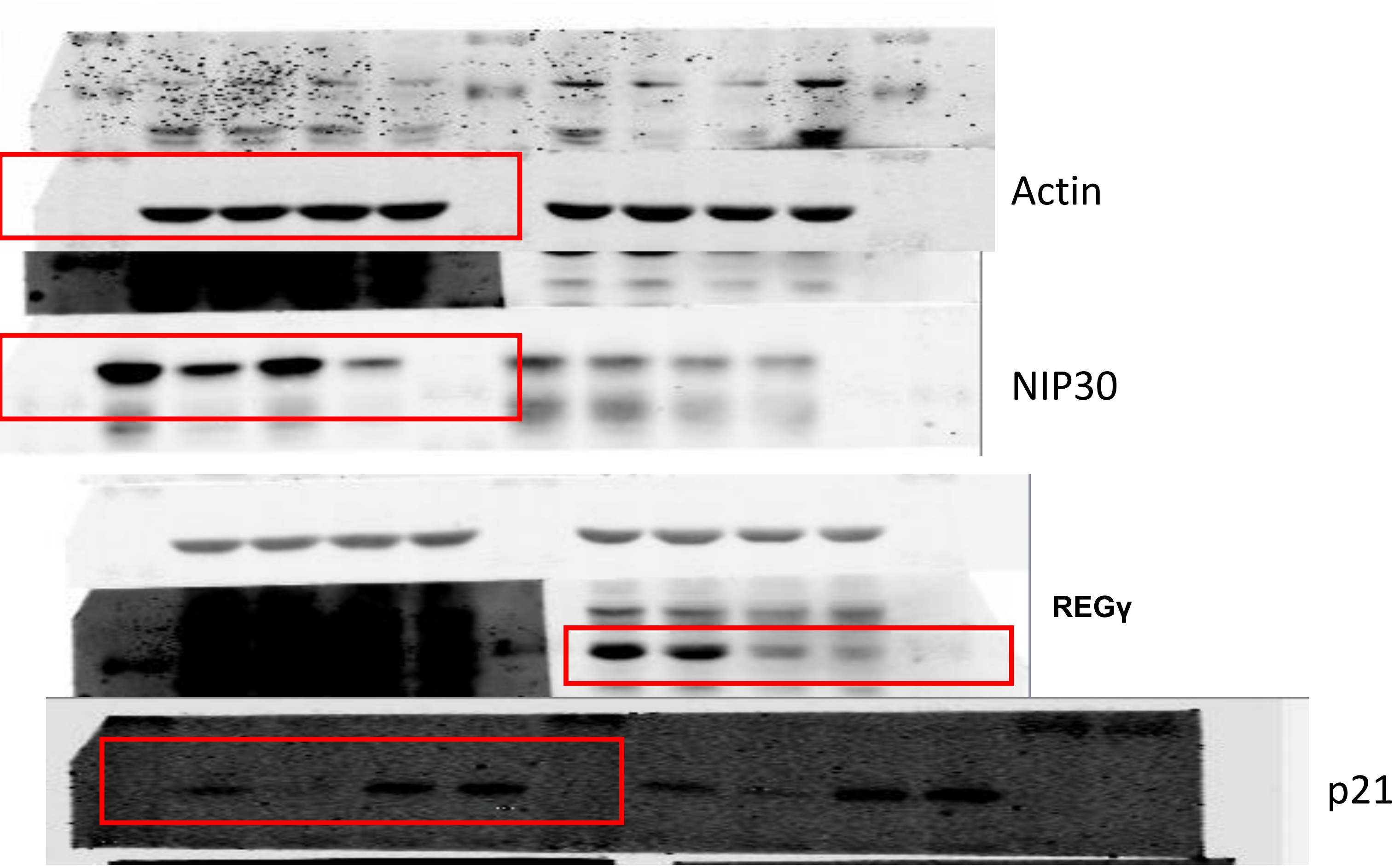

Uncropped gel images for Figure 1F

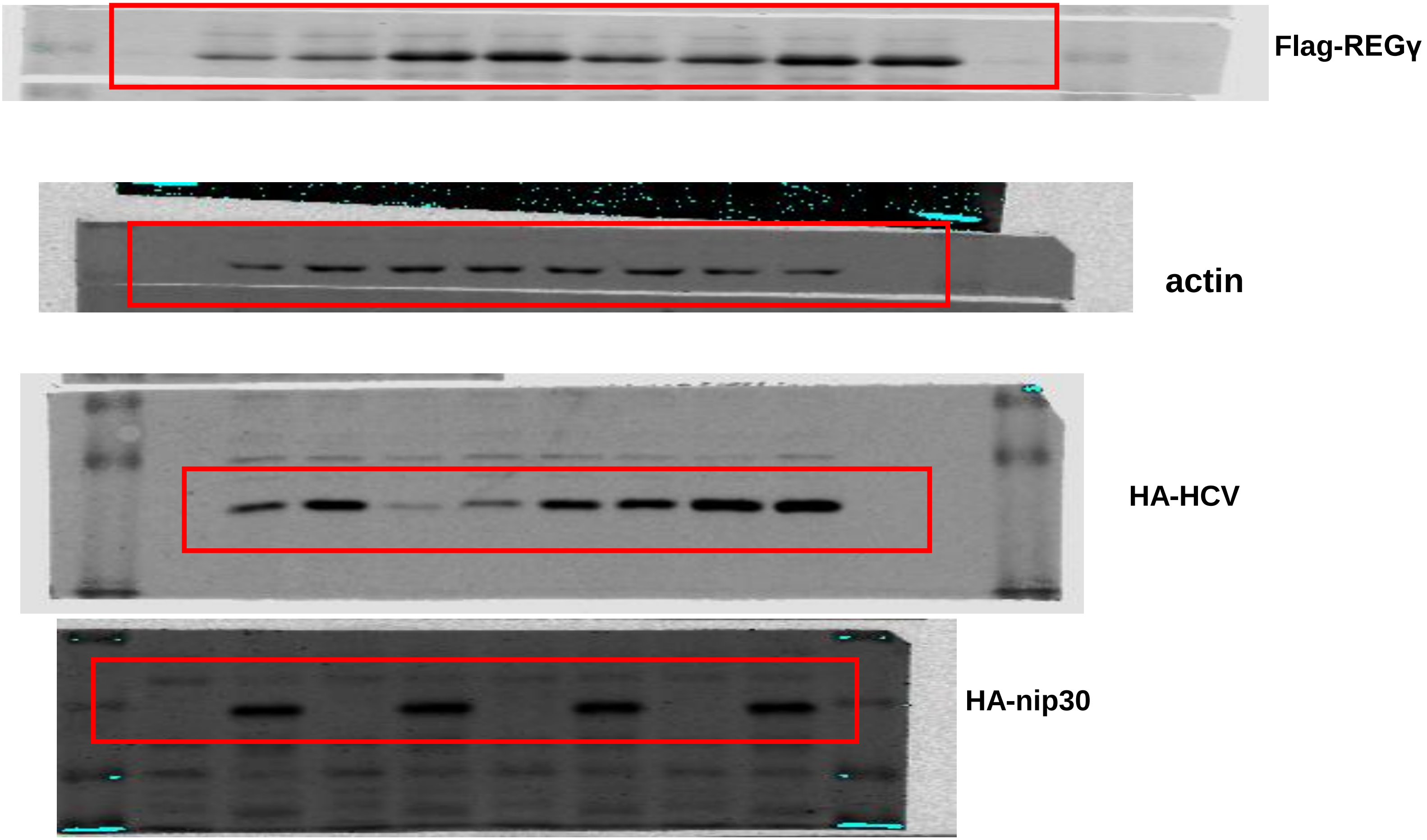

Uncropped gel images for Figure 2A

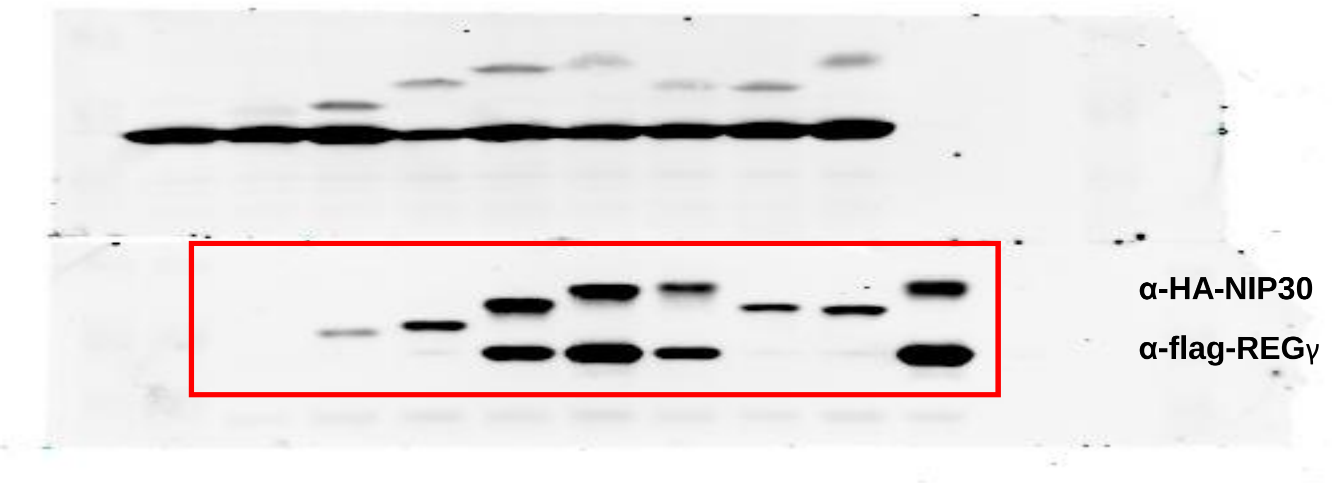

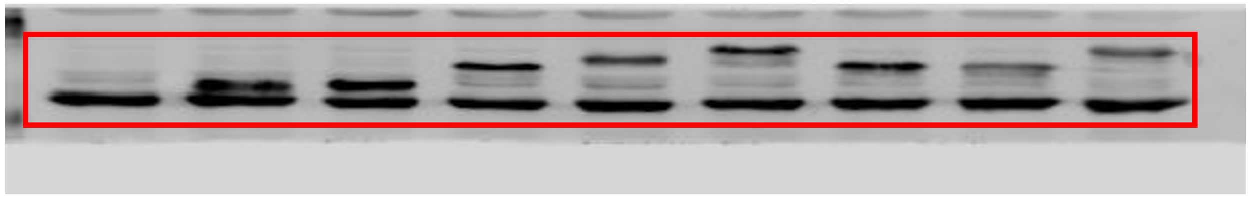

Uncropped gel images for Figure 2D

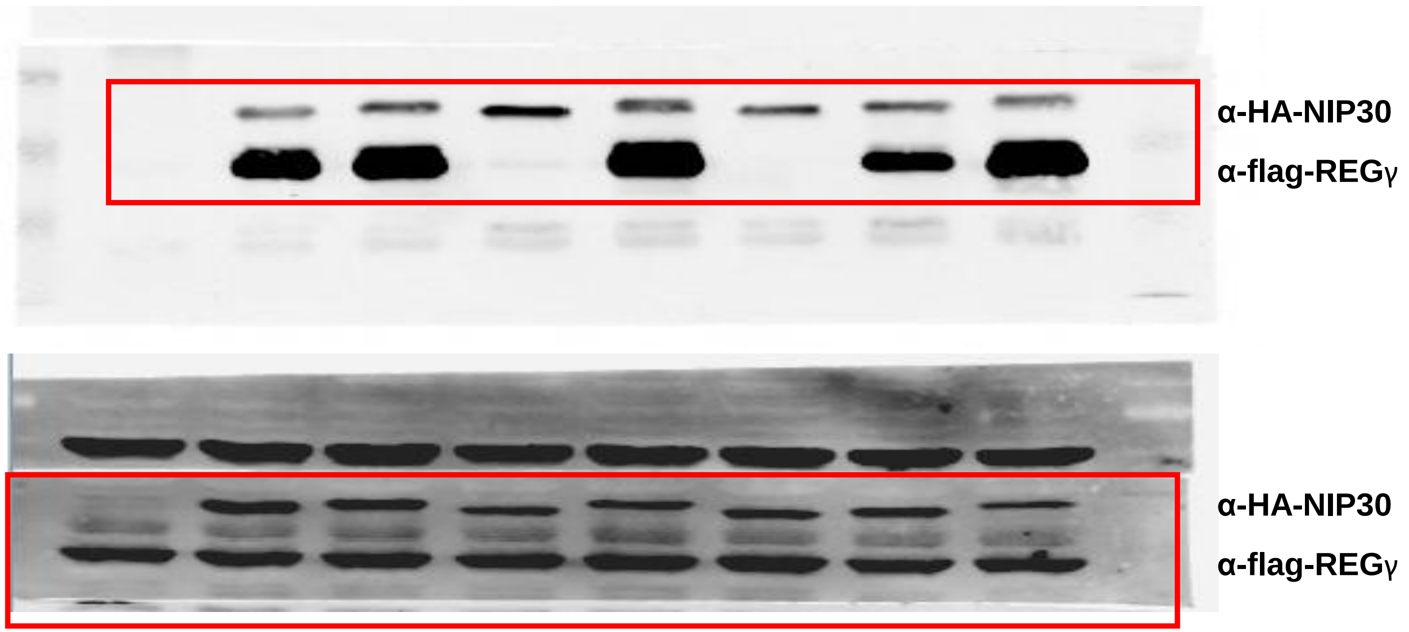

Uncropped gel images for Figure 2E

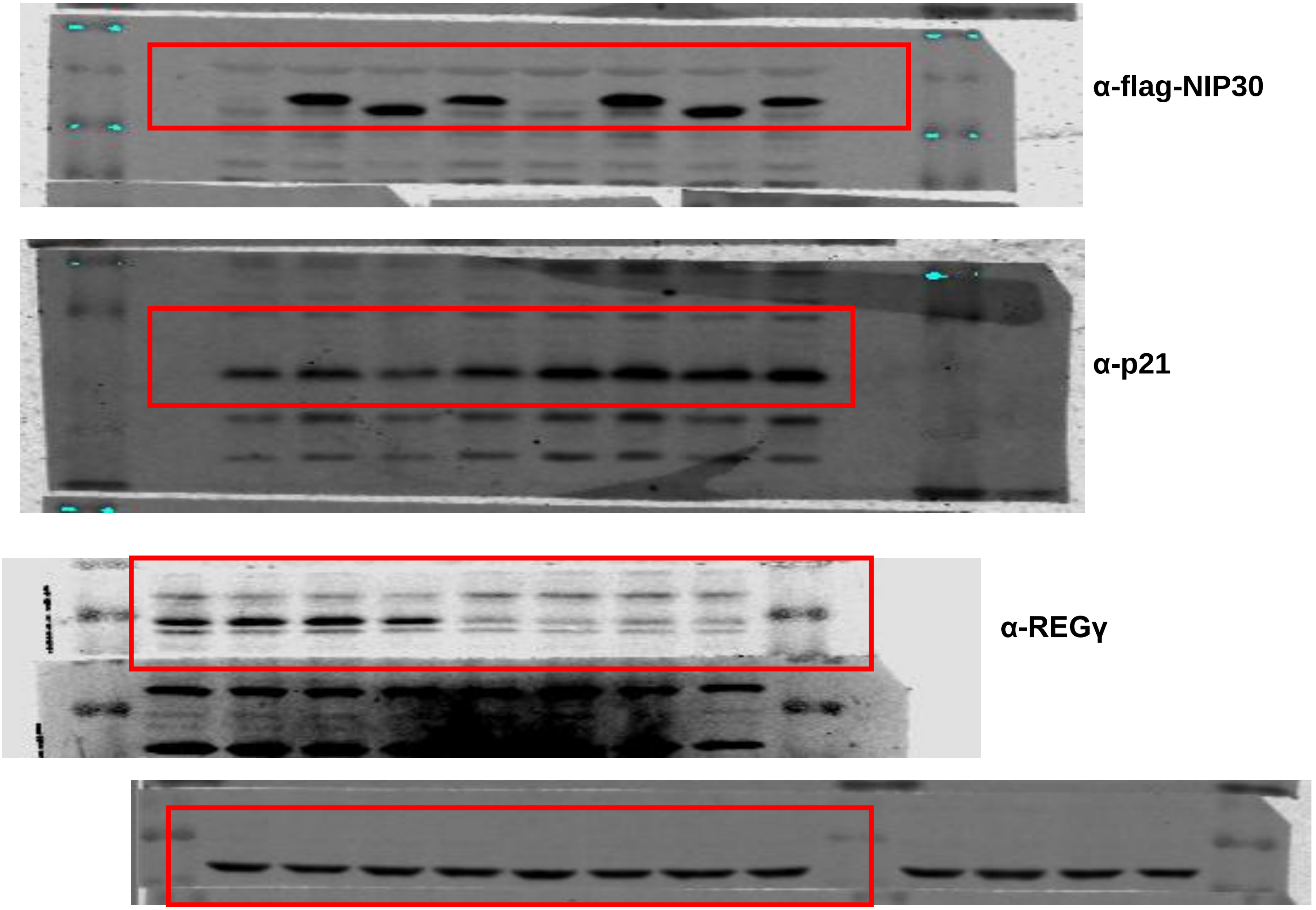

Original data for Figure 2F

A			B			C	
Data Set-A			Data Set-B			Data Set-C	
Mean	SEM	N	Mean	SEM	N	Mean	SEM
0.229500	0.006062	4	0.314640	0.018488	4	0.277312	0.036836
0.747360	0.029193	4	0.492141	0.012594	4	0.700417	0.029275
1.761825	0.071138	4	0.777936	0.015196	4	1.461350	0.031965
2.655000	0.101550	4	1.608156	0.059478	4	2.385385	0.060161

D			E			F	
Data Set-D			Data Set-E			Data Set-F	
Mean	SEM	N	Mean	SEM	N	Mean	SEM
0.226075	0.024897	4	0.203550	0.005353	4	0.180490	0.011751
0.565477	0.060934	4	0.403000	0.027295	4	0.452250	0.011222
0.831263	0.036341	4	0.704475	0.025292	4	0.730937	0.013105
1.746000	0.044202	4	1.423675	0.096436	4	1.492083	0.042914

G			H			I	
Data Set-G			Data Set-H			Title	
Mean	SEM	N	Mean	SEM	N	Mean	SEM
0.196987	0.019383	4	0.170765	0.017857	4		
0.397988	0.014991	4	0.419941	0.021998	4		
0.986200	0.052191	4	0.707296	0.019007	4		
1.573125	0.040706	4	1.466290	0.046259	4		

Uncropped images for 2G

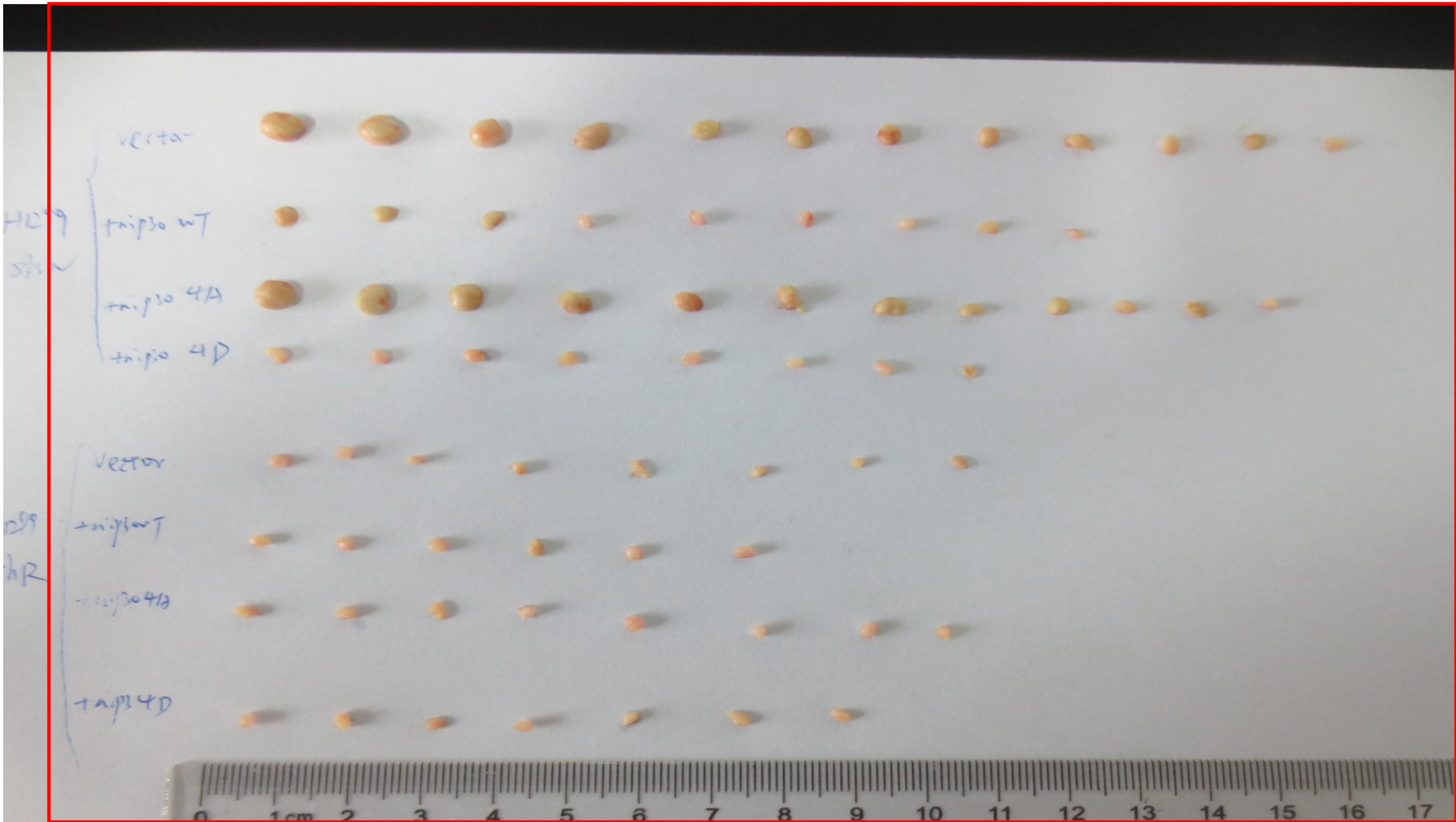

Uncropped gel images for Figure3A

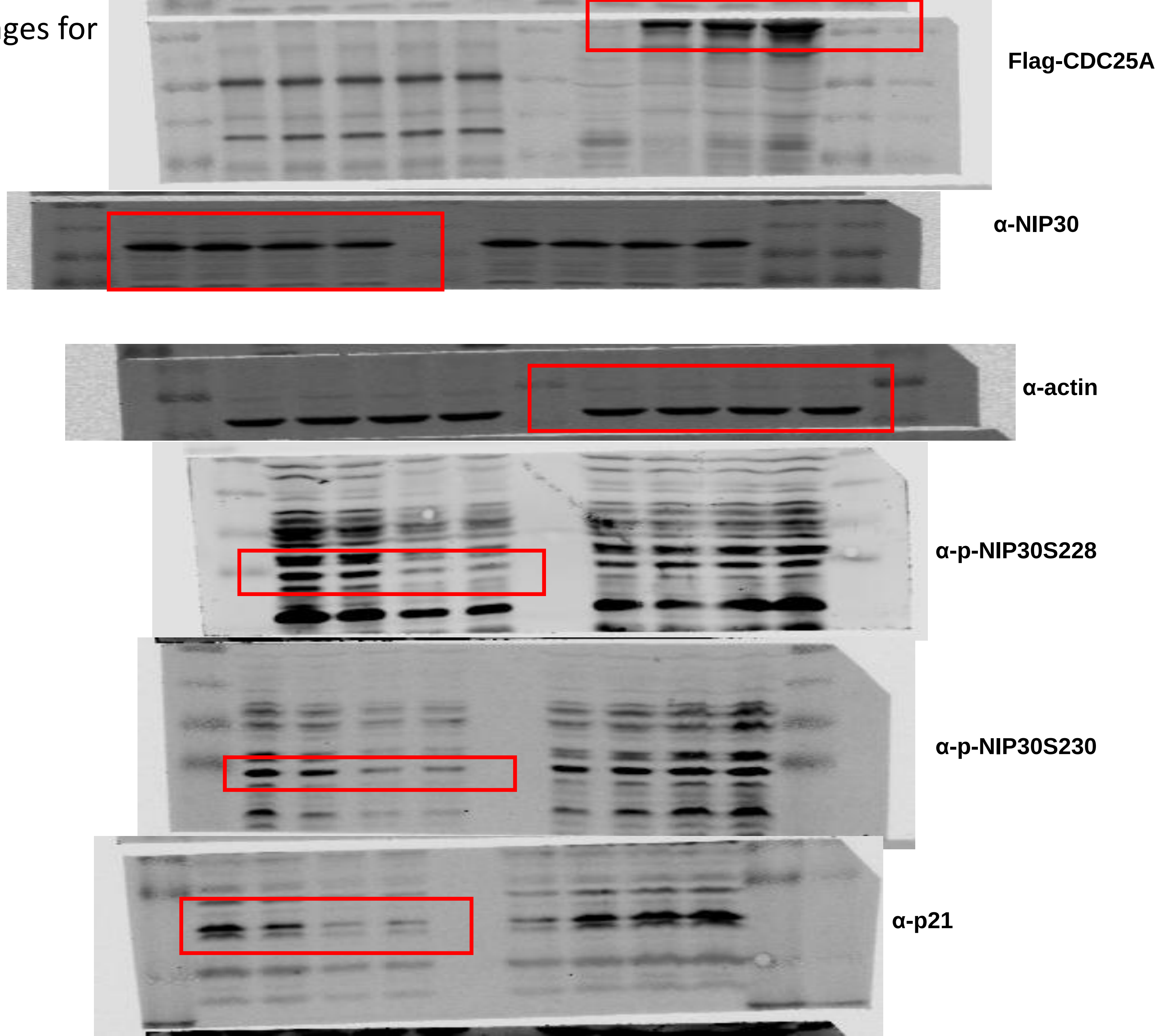

Uncropped gel images for Figure3B

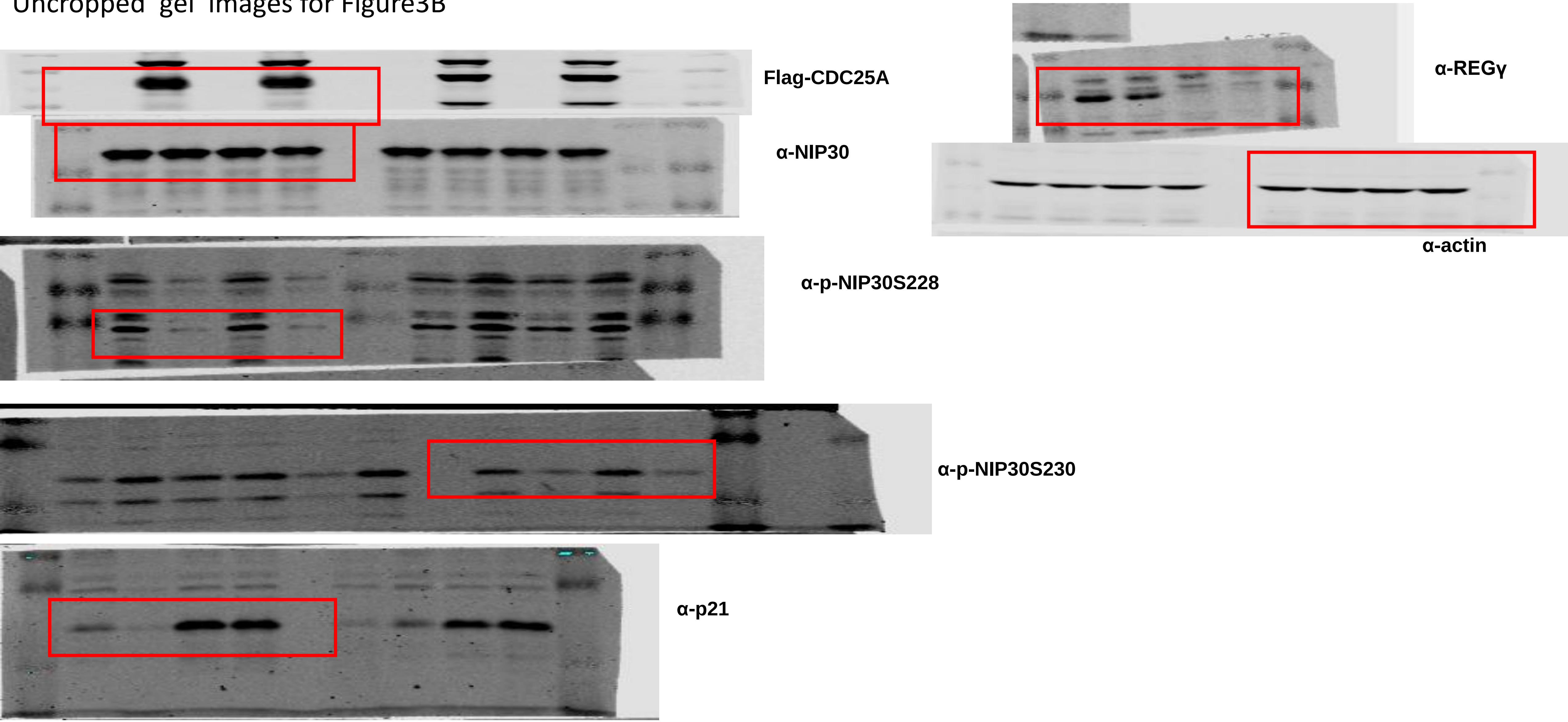

Uncropped gel images for Figure3C

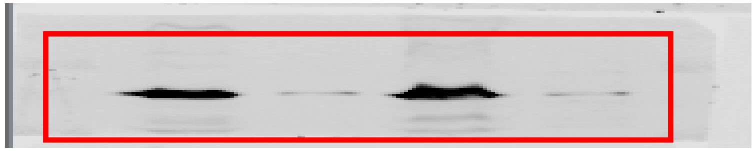

α-CDC25A

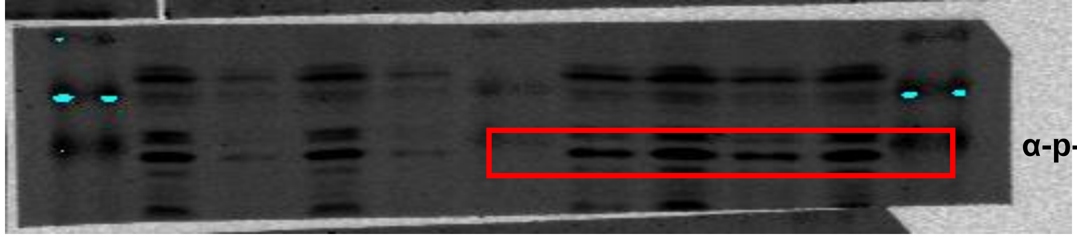

α-p-NIP30S230

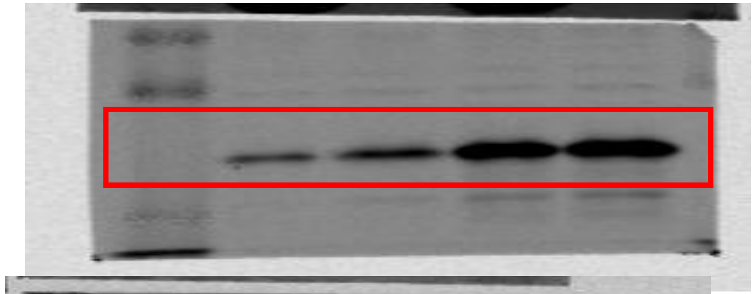

α-p21

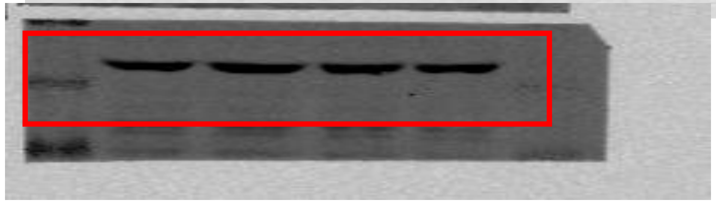

α-NIP30

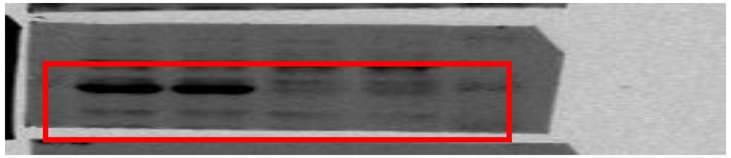

α-REGγ

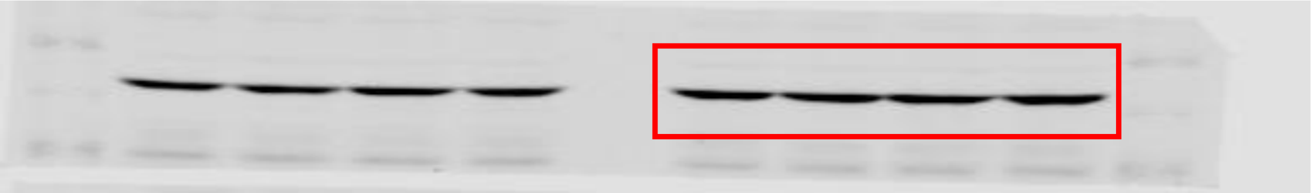

α-actin

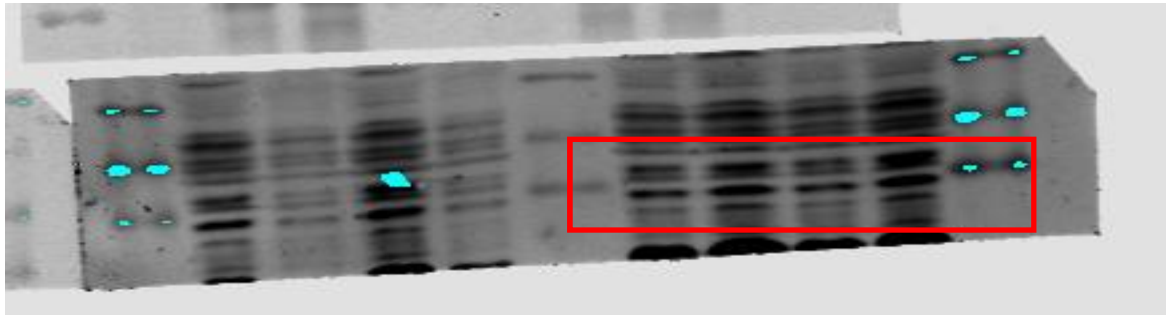

α-p-NIP30S228

Uncropped gel images for Figure3D

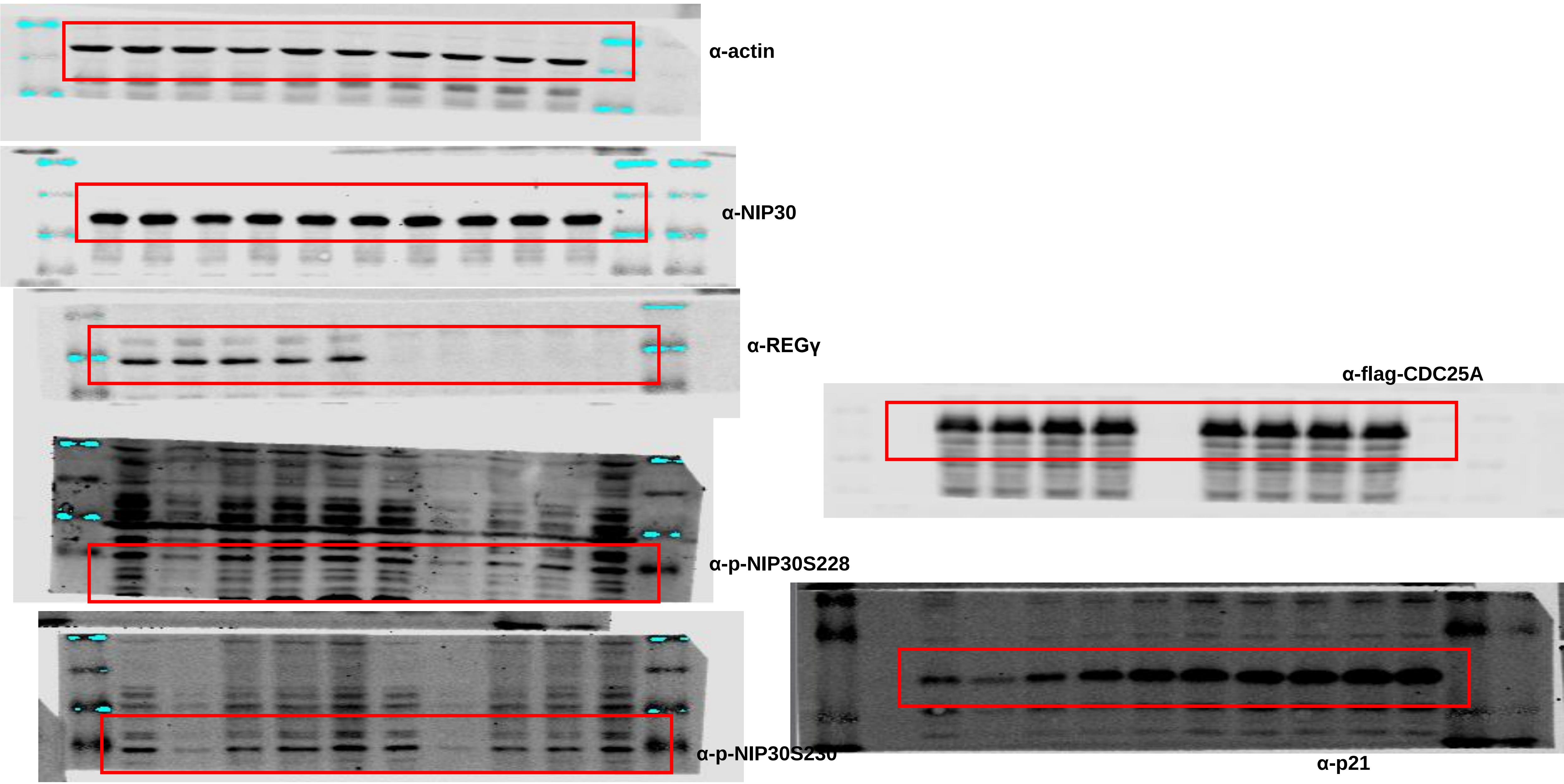

Uncropped gel images for Figure3E

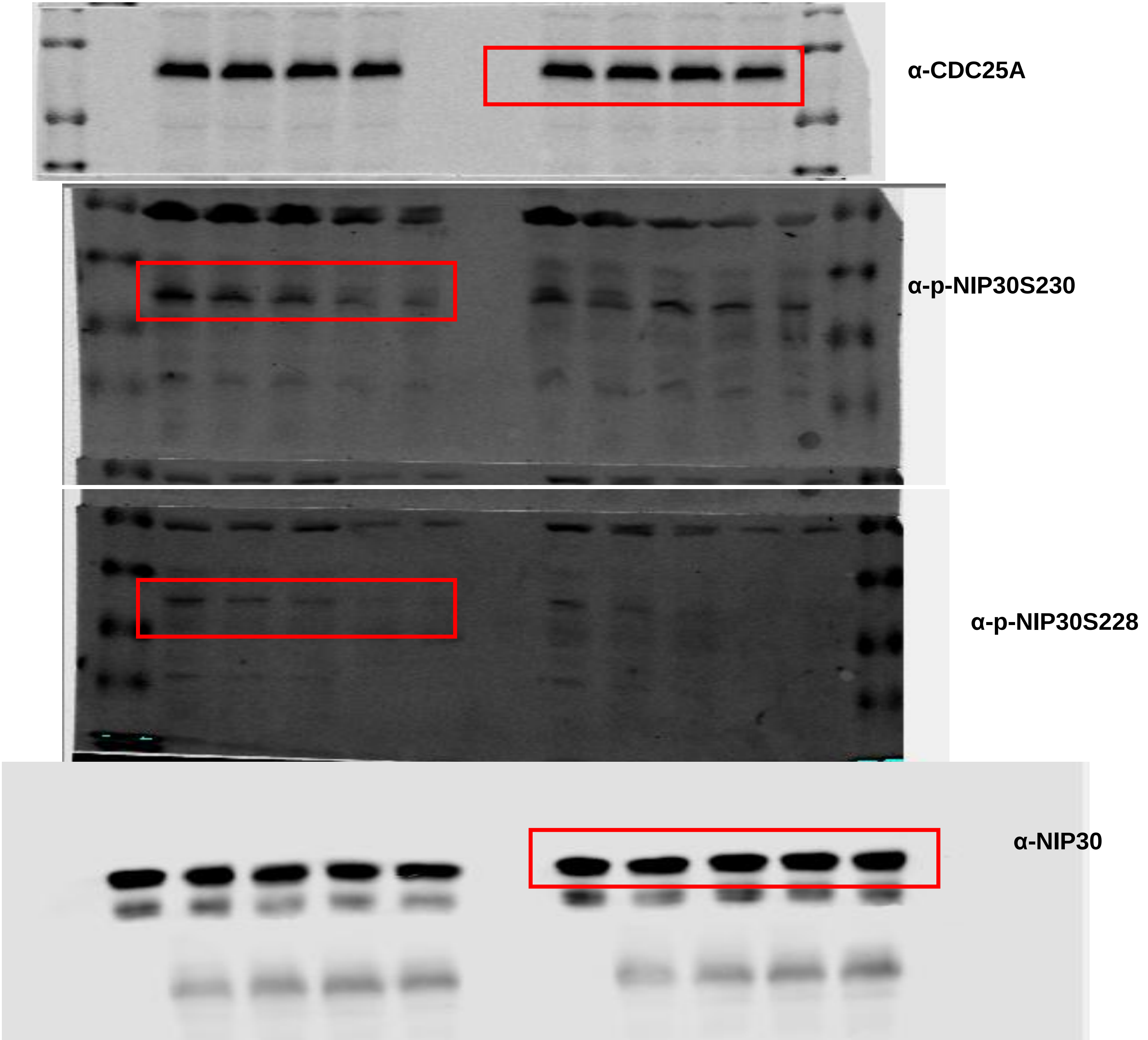

Uncropped gel images for Figure3F

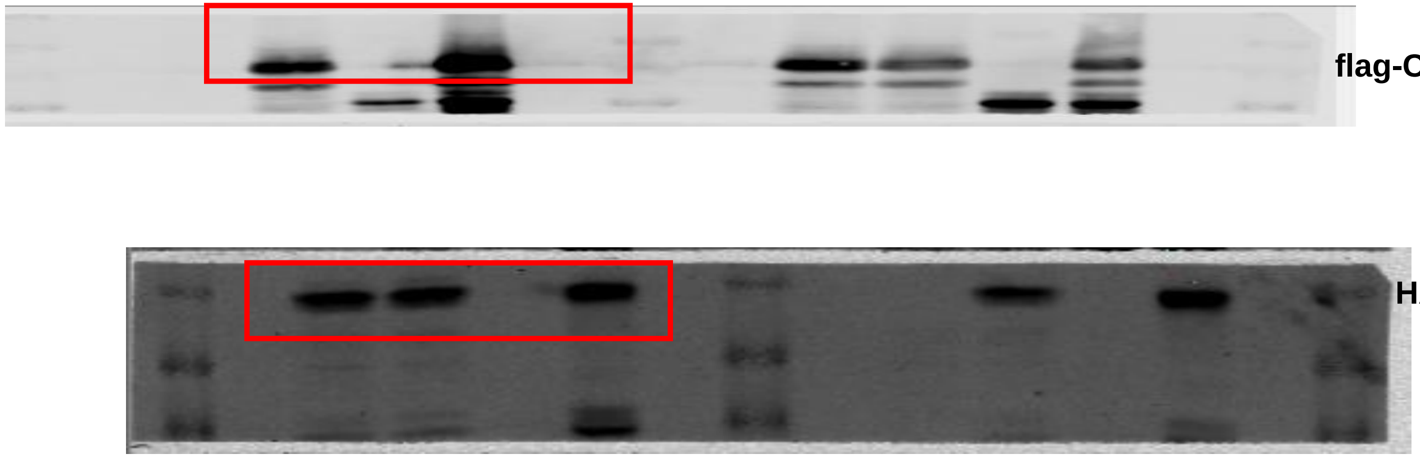

flag-CDC25A

HA-NIP30

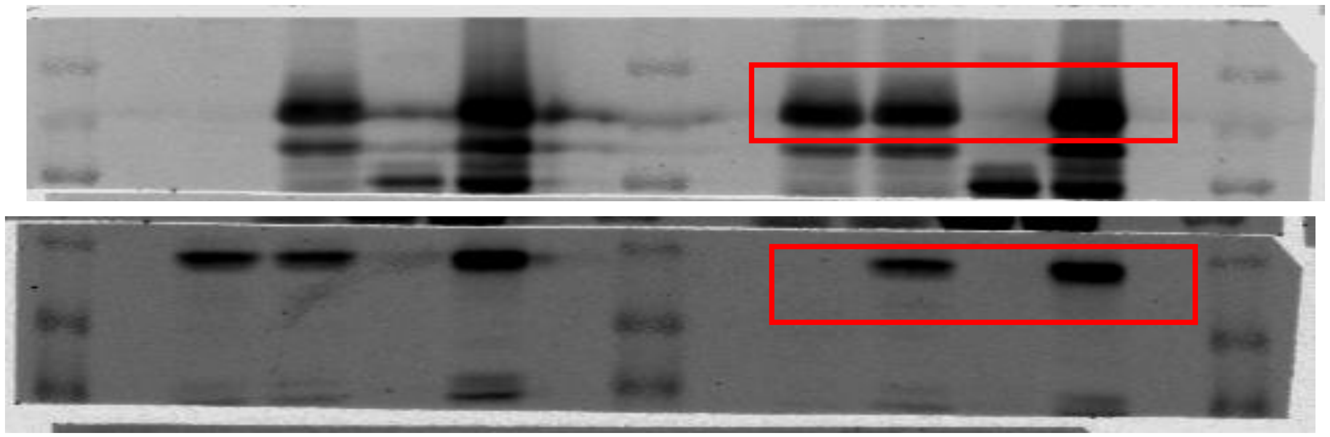

flag-CDC25A

HA-NIP30

Uncropped gel images for Figure 4A

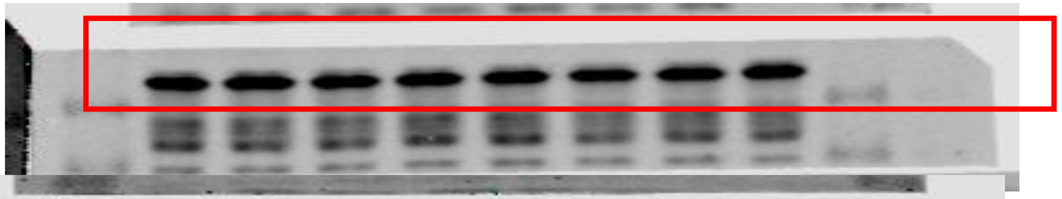

α-actin

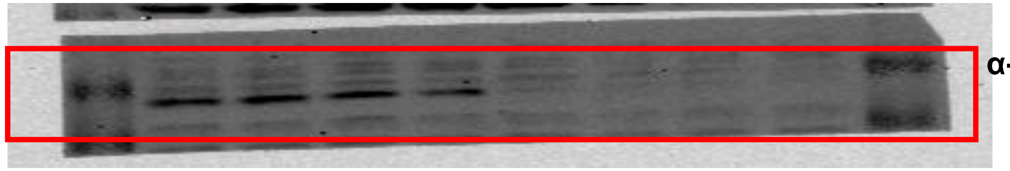

α-REGγ

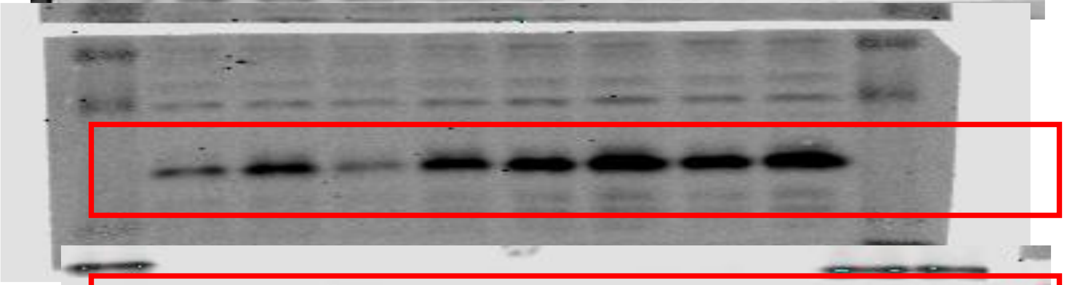

α-p21

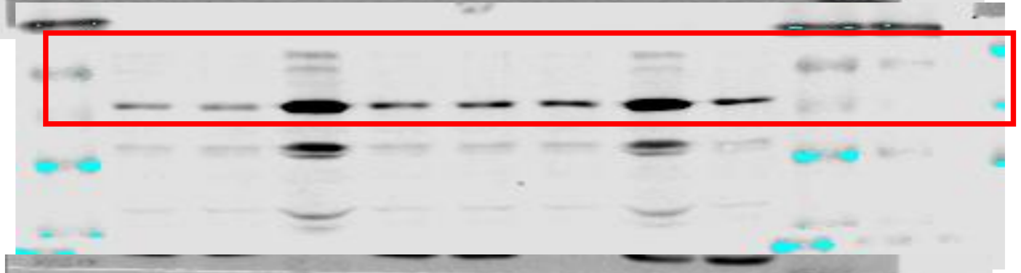

α-CDC25A

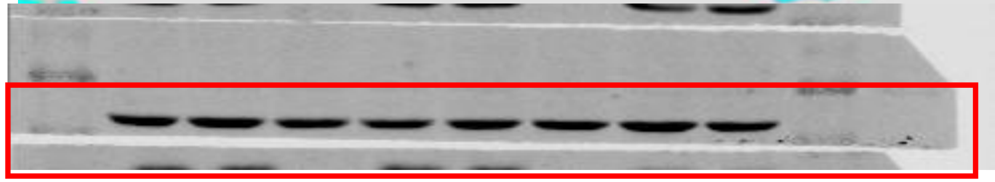

α-actin

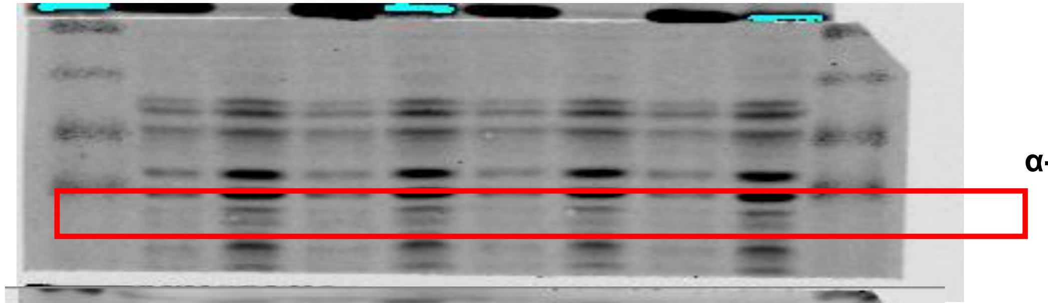

α-p-NIP30S228

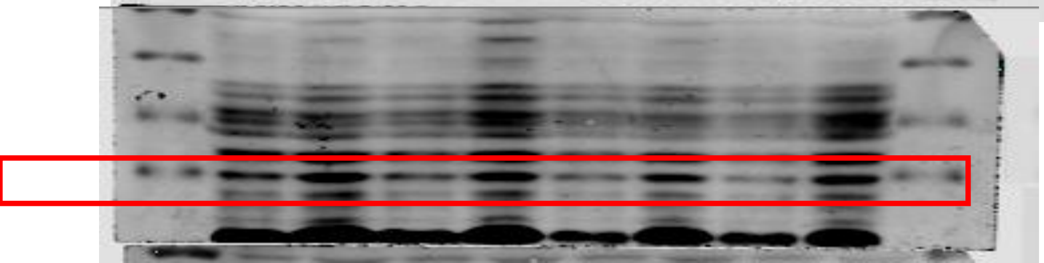

α-p-NIP30S230

Uncropped gel images for Figure 4B

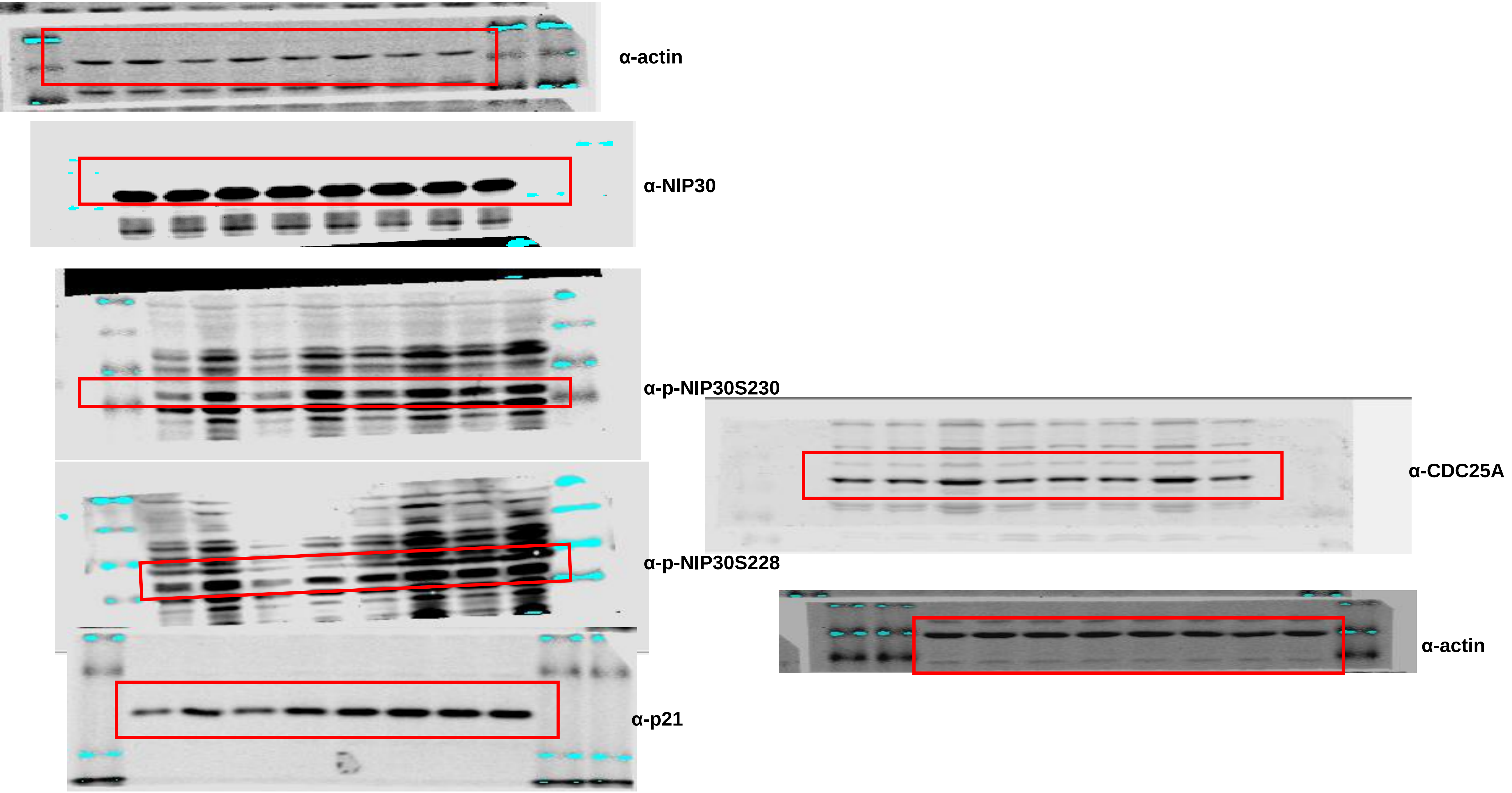

Uncropped gel images for Figure 4C

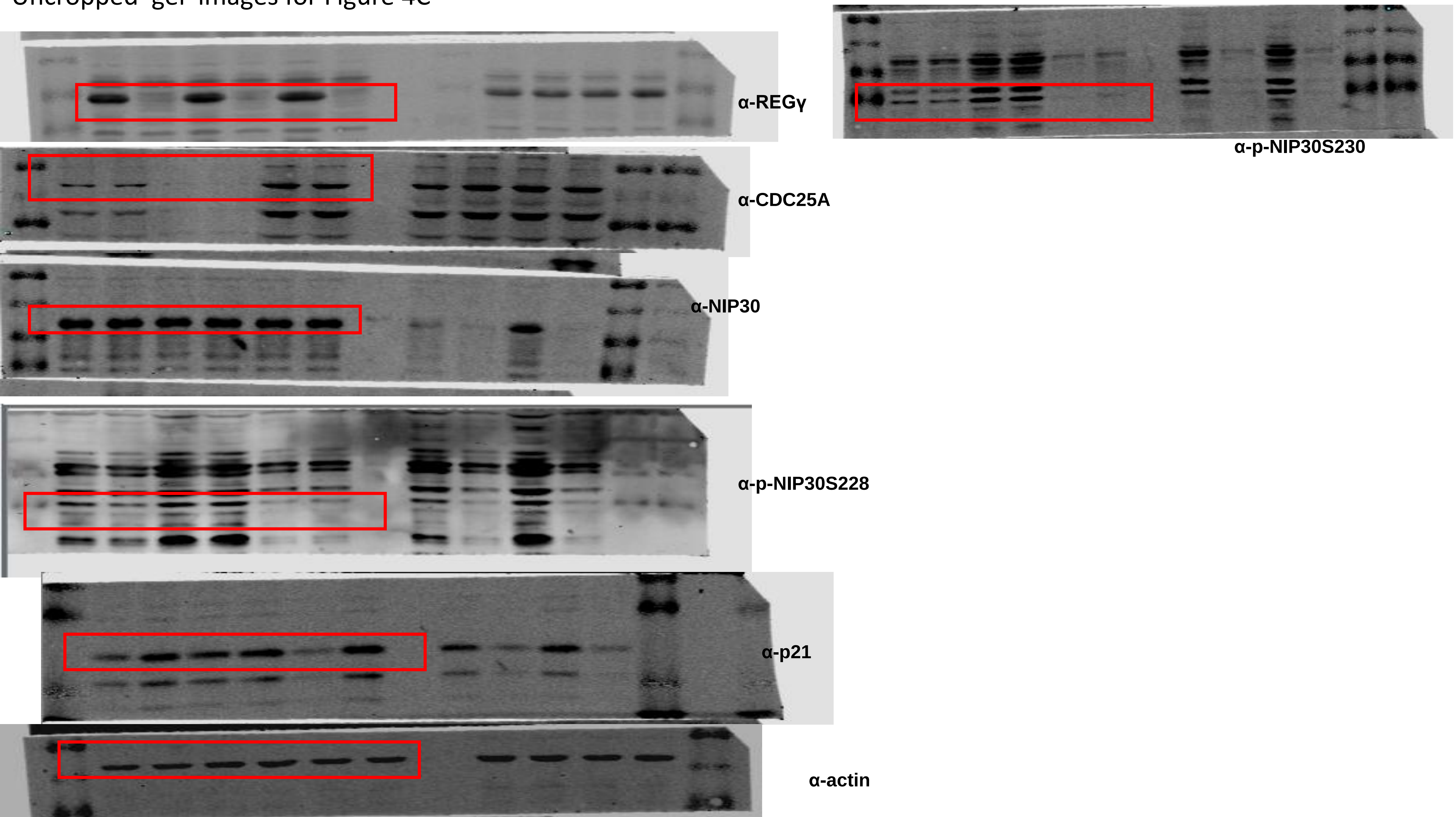

Uncropped gel images for Figure 4D

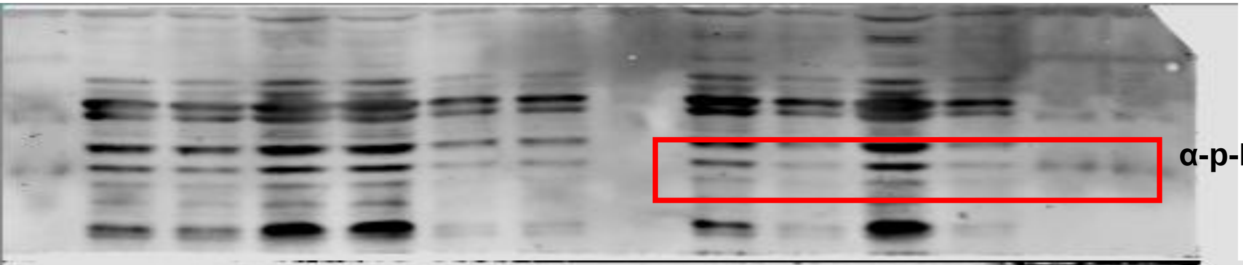

α-p-NIP30S228

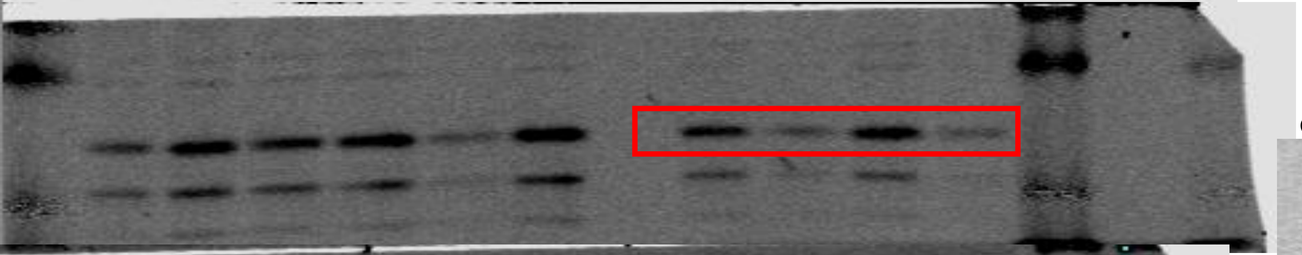

α-NIP30

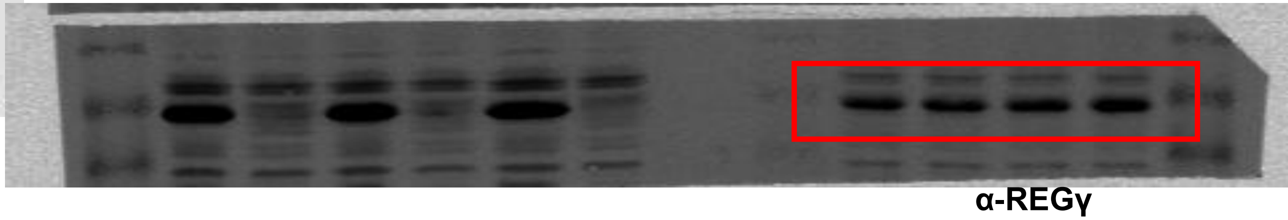

α-REGγ

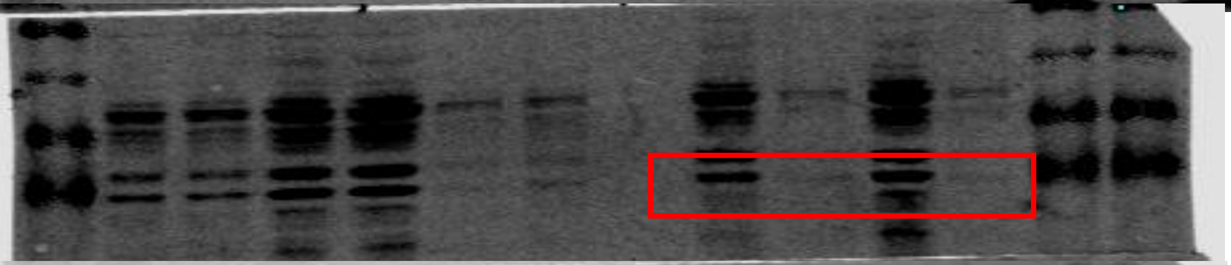

α-p-NIP30S228

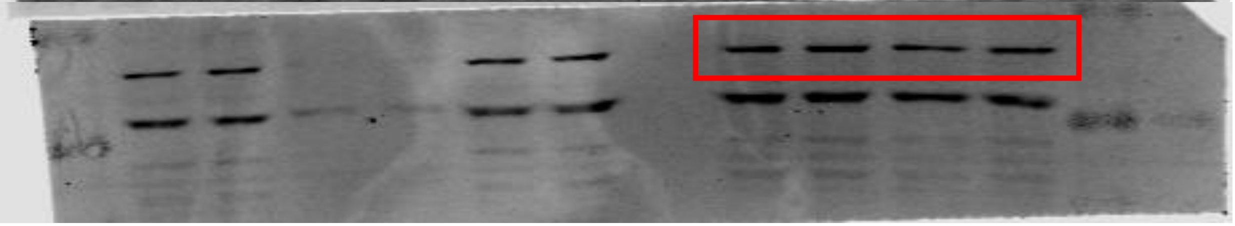

α-CDC25A

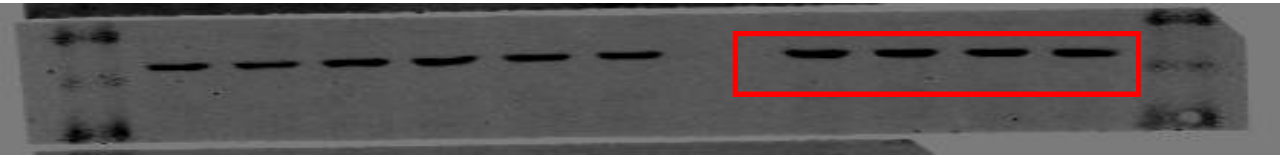

α-actin

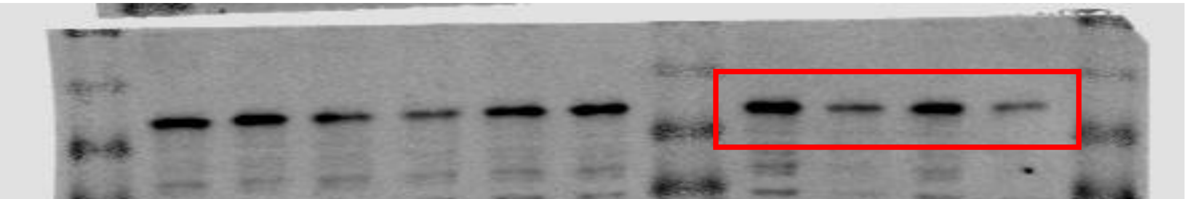

α-p-NIP30S230

Uncropped gel images for Figure 4E

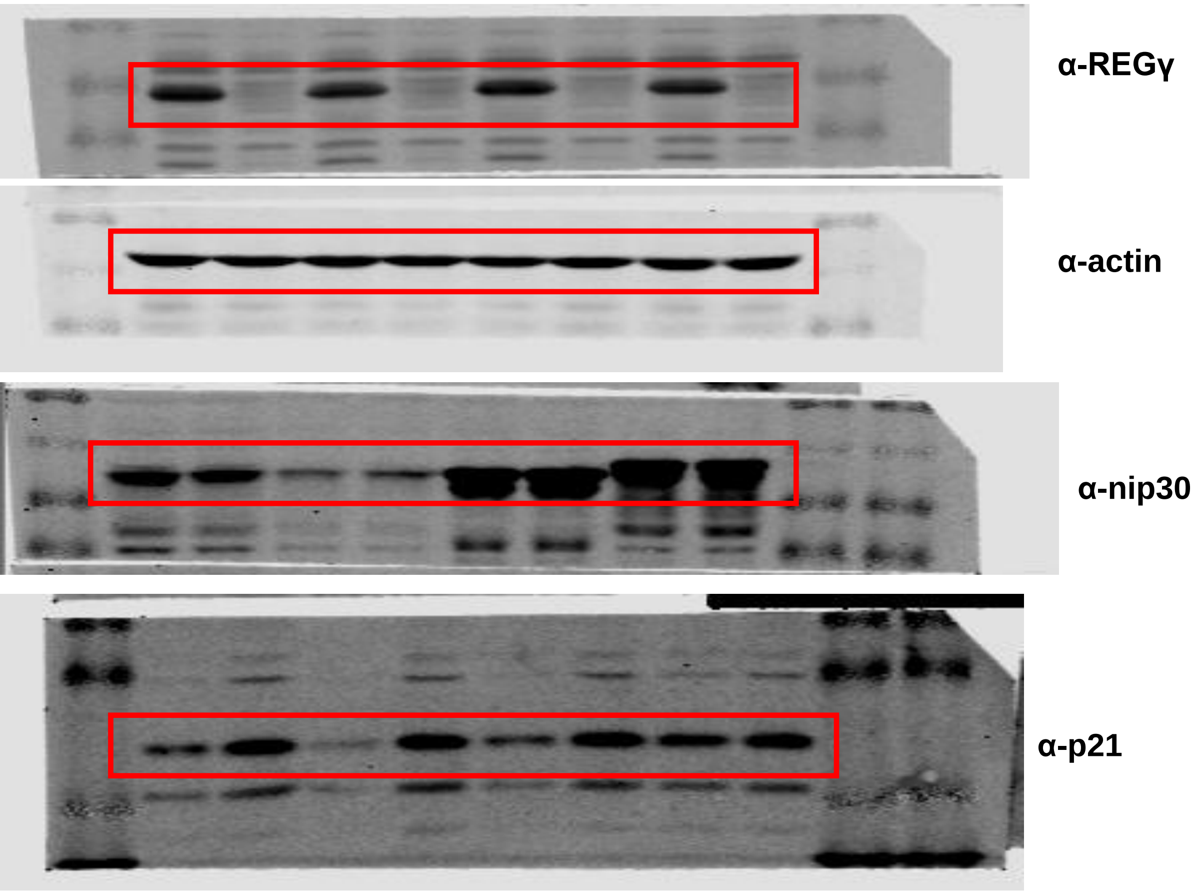

Uncropped gel images for Figure 5A

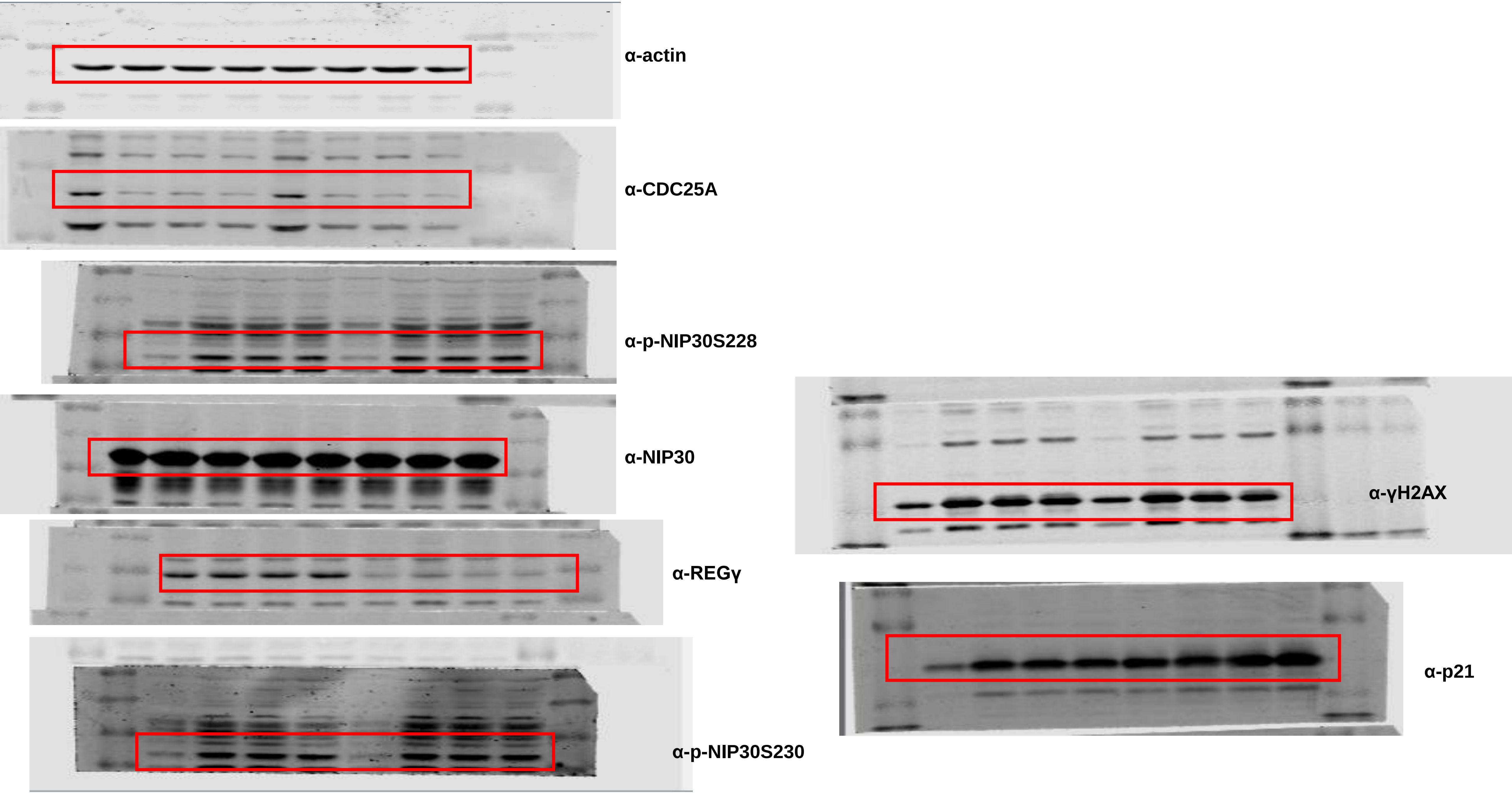

Uncropped gel images for Figure 5B

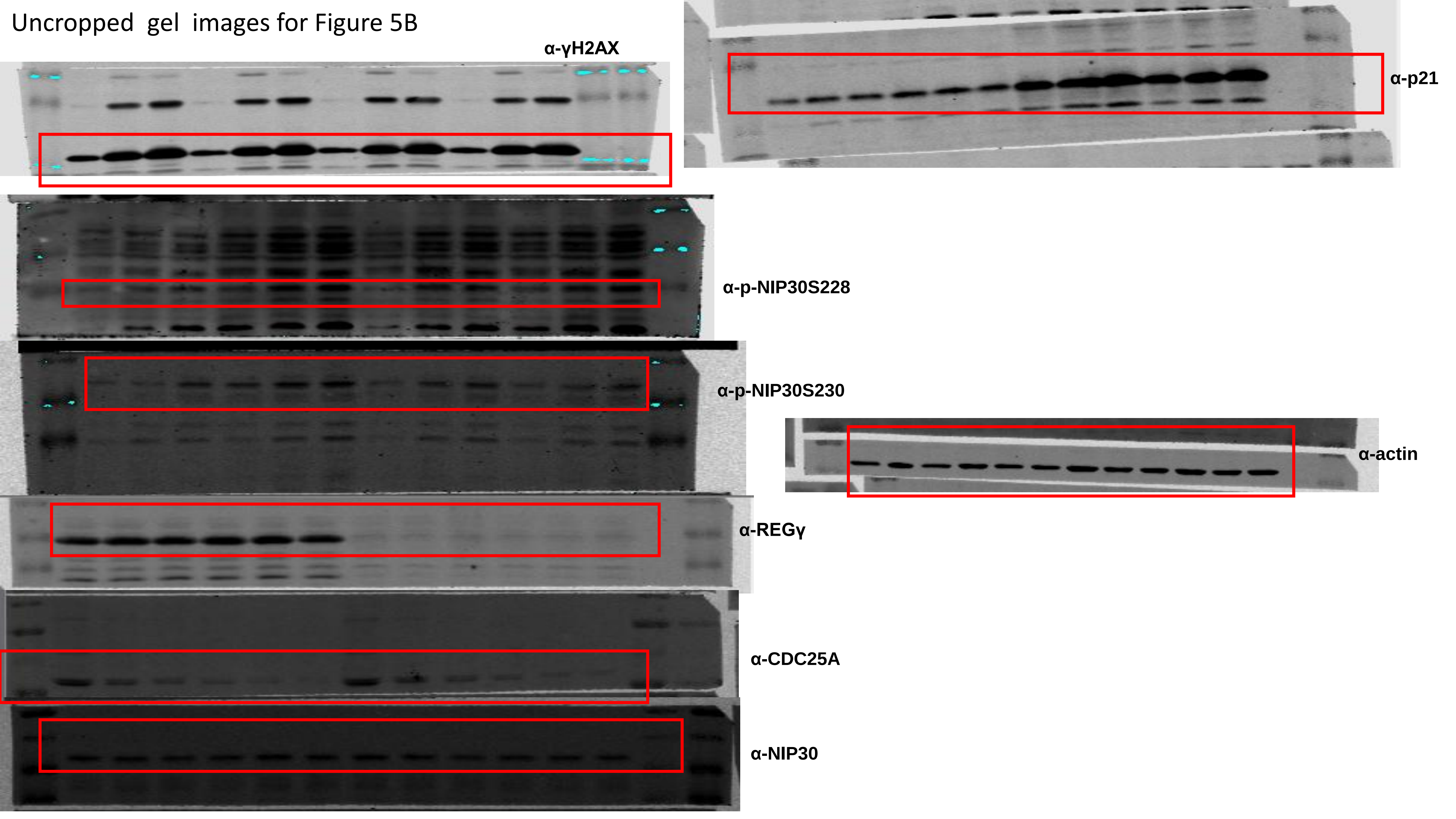

Uncropped gel images for Figure 5C

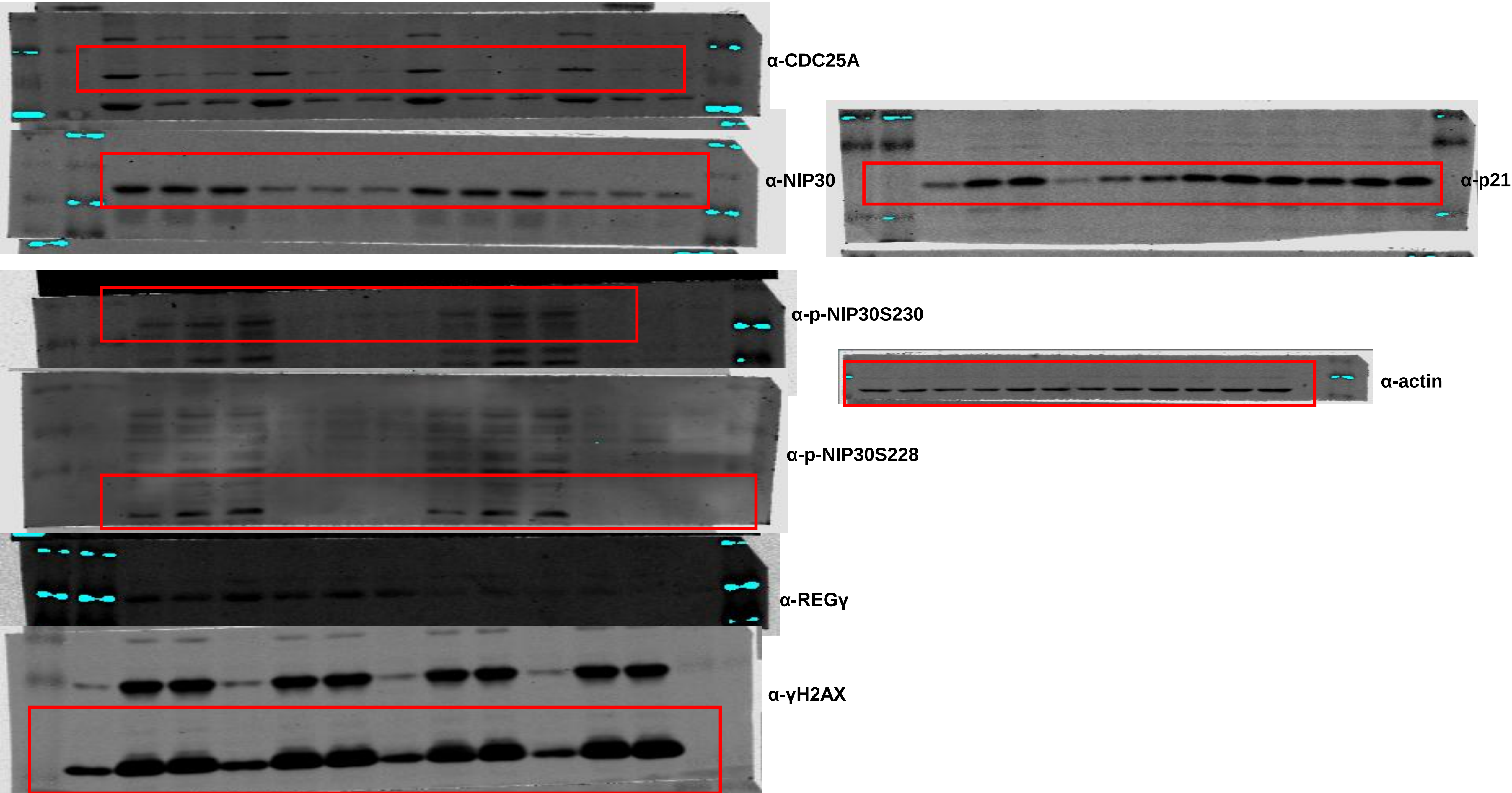

Uncropped gel images for Figure 5D

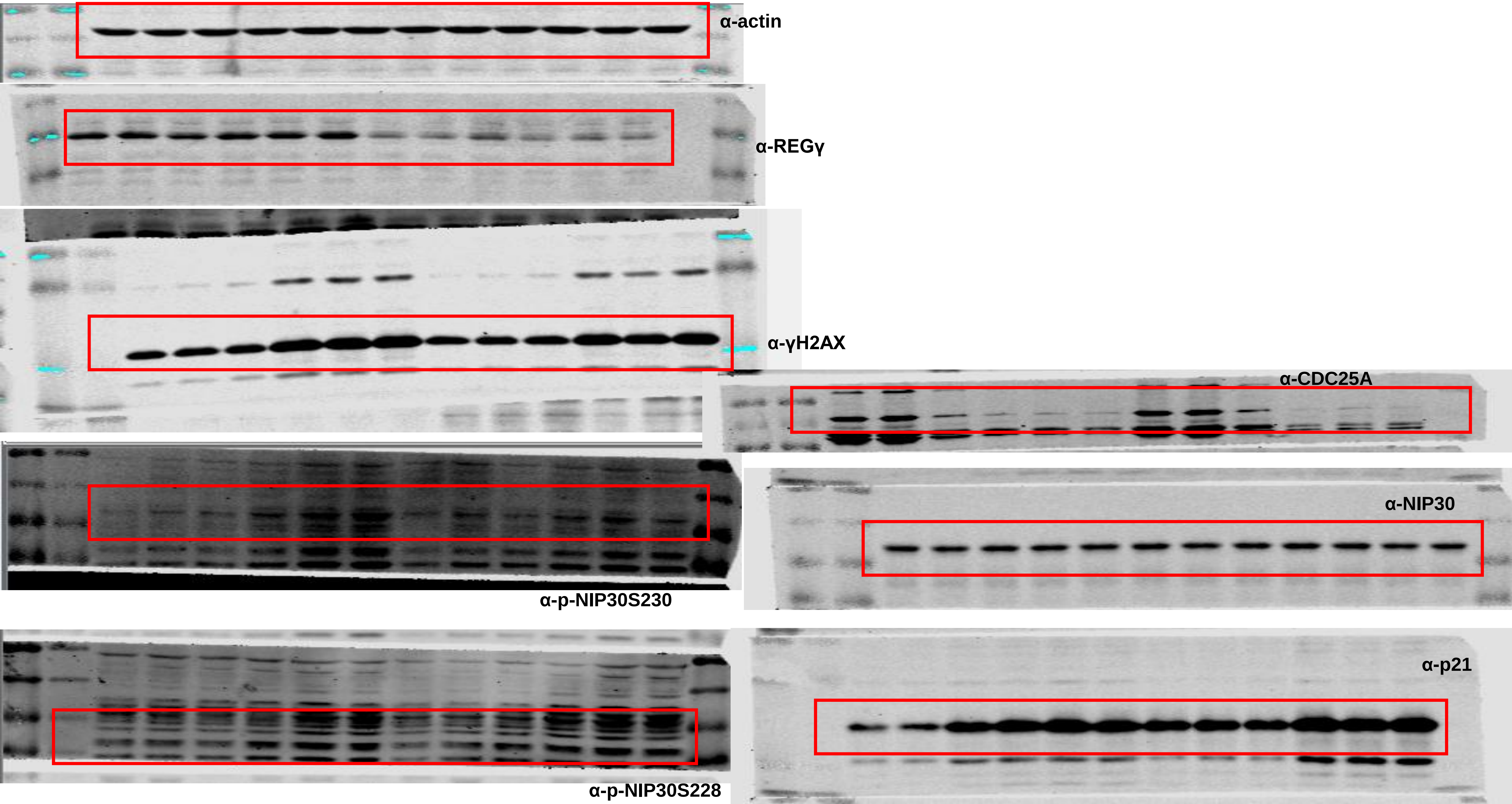

Uncropped gel images for Figure 5E

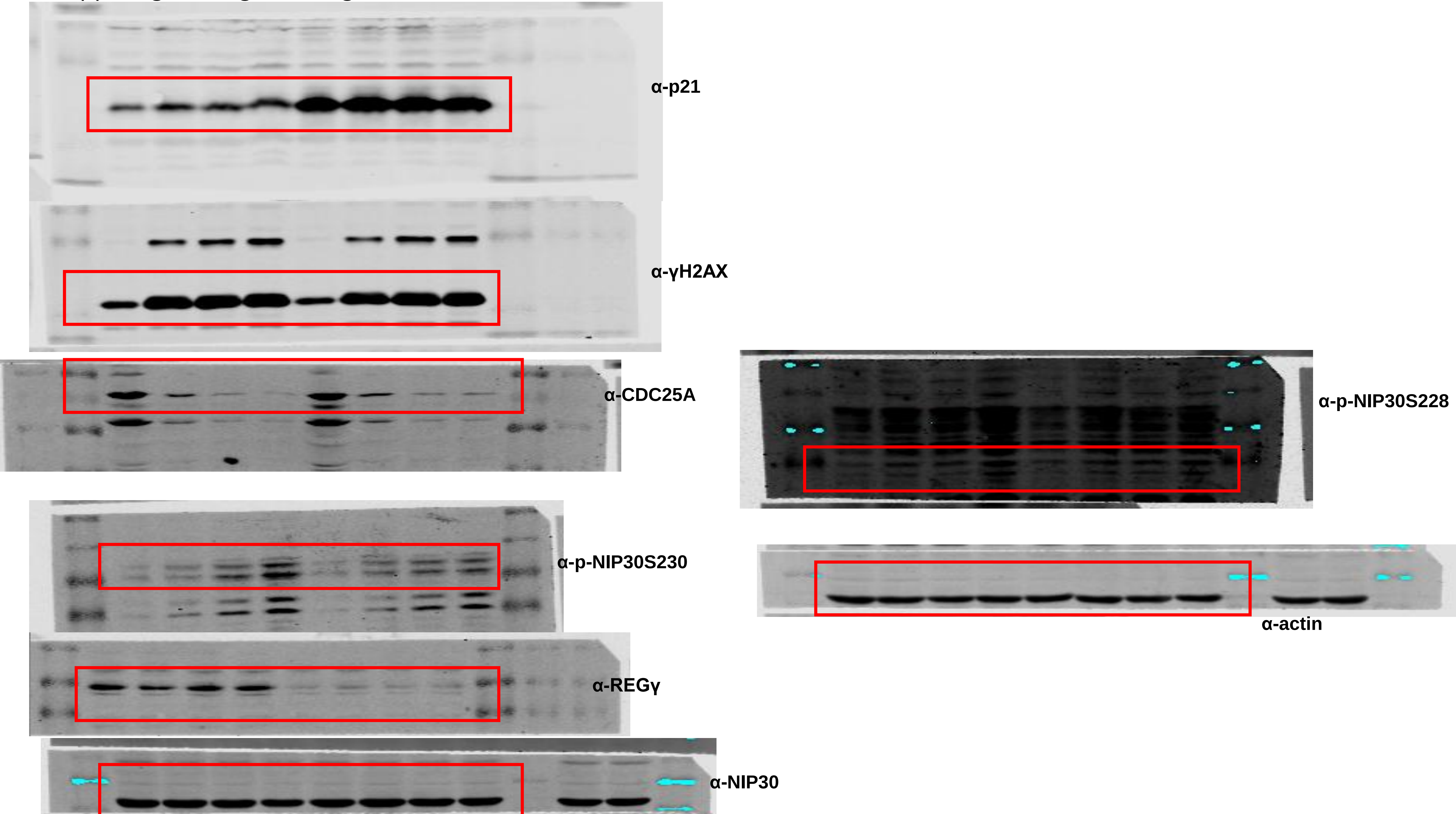

Uncropped gel images for Figure 5F

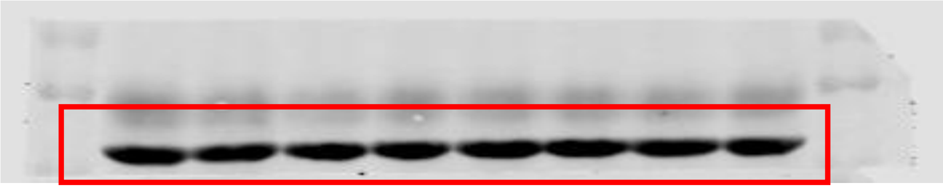

α-actin

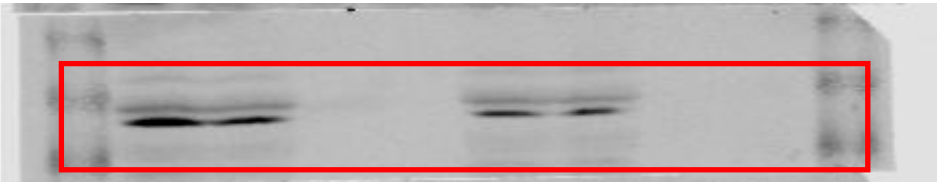

α-REGγ

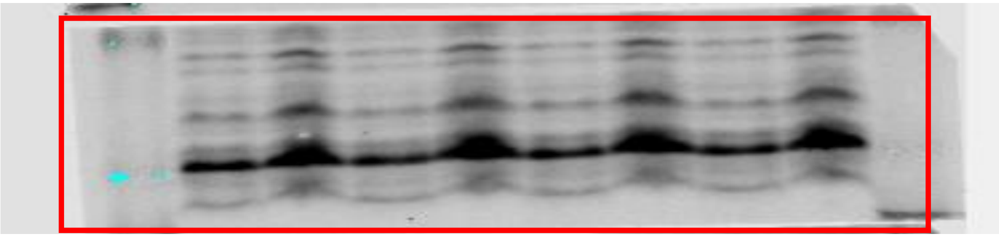

α-γH2AX

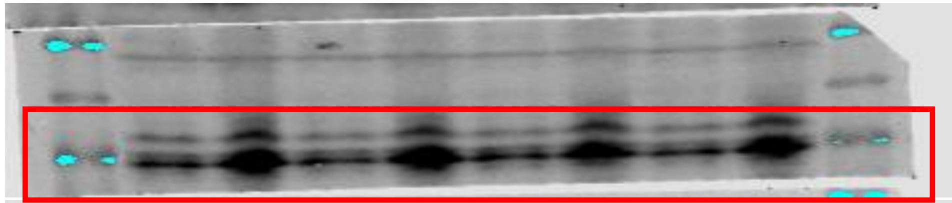

α-p-NIP30S228

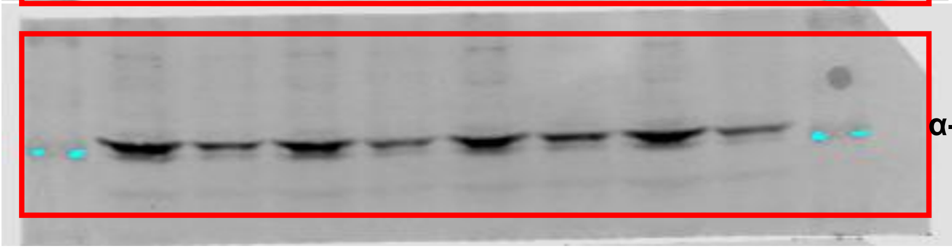

α-CDC25A

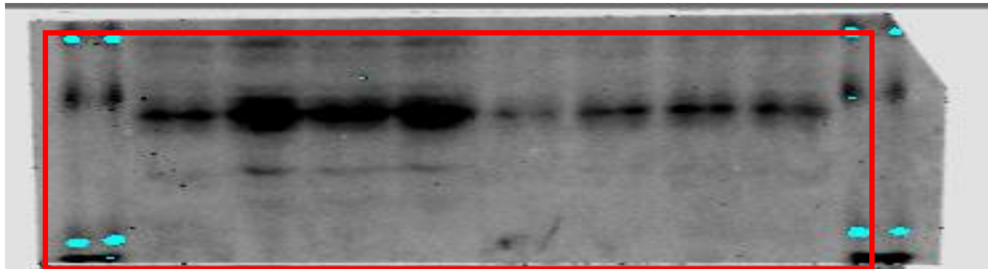

α-p21

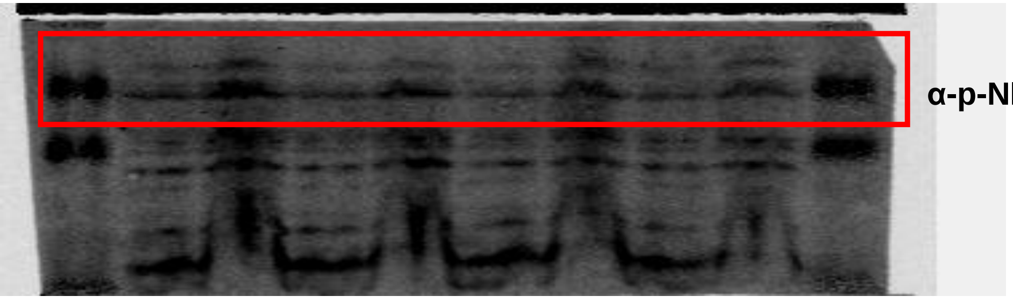

α-p-NIP30S230

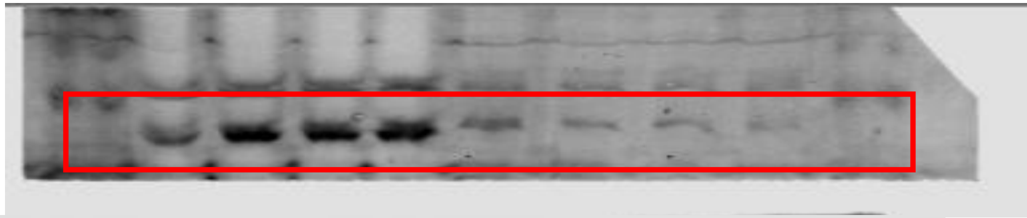

α-p53

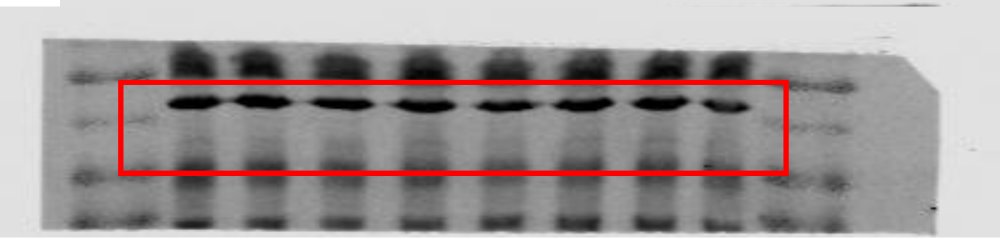

α-NIP30

Original data for Figure 6A

	Cancer Ty	Protein C	Mutation	Chromosom	Start Pos	End Pos	Ref	Var
2	Breast Ir	P198L	Missense_	16	57188374	57188374	G	A
3	Chromophc	S228L	Missense_	16	57188284	57188284	G	A
4	Prostate	D83G	Missense_	16	57206263	57206263	T	C
5	Stomach A	S196R	Missense_	16	57188381	57188381	T	G
6	Stomach A	A150V	Missense_	16	57201038	57201038	G	A
7	Head and	K188T	Missense_	16	57188404	57188404	T	G
8	Head and	E108del	In_Frame_	16	57206187	57206189	TTC	–
9	Uterine E	E111*	Nonsense_	16	57206180	57206180	C	A
10	Uterine E	E101K	Missense_	16	57206210	57206210	C	T
11	Uterine S	R24W	Missense_	16	57207697	57207697	G	A
12	Uterine E	Q149H	Missense_	16	57201040	57201040	C	A
13	Uterine E	K129R	Missense_	16	57201101	57201101	T	C
14	Lung Squa	R27M	Missense_	16	57207687	57207687	C	A
15	Mucinous	P213S	Missense_	16	57188330	57188330	G	A
16	Mucinous	R104Q	Missense_	16	57206200	57206200	C	T
17	Renal Cle	X5_splice	Splice_Si	16	57207783	57207783	T	A
18	Renal Cle	X5_splice	Splice_Si	16	57207783	57207783	T	A
19	Head and	E231K	Missense_	16	57188276	57188276	C	T
20	Hepatocel	D178E	Missense_	16	57197926	57197926	A	T
21	Breast Ir	X43_splic	Splice_Re	16	57206793	57206793	G	A
22	Cutaneous	S224F	Missense_	16	57188296	57188296	G	A
23	Cutaneous	D91del	In_Frame_	16	57206236	57206238	TCA	–

Original data for Figure 6B

able format: Grouped	A			B			C		
	Data Set-A			Data Set-B			Data Set-C		
	Mean	SEM	N	Mean	SEM	N	Mean	SEM	N
0	100.000000	5.972148	4	100.000000	4.391329	4	100.000000	4.385837	4
5	91.414250	4.061247	4	81.982270	6.515805	4	93.861050	4.623367	4
10	81.745380	2.753384	4	55.856880	8.748584	4	84.004310	4.353581	4
20	63.782320	3.019455	4	42.187200	4.951428	4	66.704410	1.640015	4
40	40.956460	1.190171	4	31.490410	3.186132	4	49.904200	3.060161	4

D		
Data Set-D		
Mean	SEM	N
100.000000	7.095990	4
76.427410	1.727302	4
43.348980	3.913222	4
32.576210	1.626112	4
22.007980	2.542746	4

able format: Grouped	A			B			C		
	Data Set-A			Data Set-B			Data Set-C		
	Mean	SEM	N	Mean	SEM	N	Mean	SEM	N
0	100.000000	13.451470	4	100.000000	3.948328	4	100.000000	2.032693	4
25	78.769350	7.240422	4	74.529500	7.315637	4	91.393910	6.433432	4
50	69.131680	7.046267	4	51.334320	6.812981	4	81.368160	6.426945	4
100	56.934420	8.331352	4	42.748060	6.117490	4	71.215990	7.906507	4
200	43.857440	3.951889	4	30.783420	6.036523	4	60.972190	6.722385	4

D		
Data Set-D		
Mean	SEM	N
100.000000	3.798597	4
69.961360	3.882934	4
42.301280	3.301097	4
26.210940	7.995817	4
21.036270	6.738930	4

able format: Grouped	B			C			D		
	Data Set-B			Data Set-C			Data Set-D		
	Mean	SEM	N	Mean	SEM	N	Mean	SEM	N
0	100.000000	3.394450	4	100.000000	4.353409	4	100.000000	7.687875	4
5	66.832700	4.671573	4	90.496620	4.860917	4	57.364370	9.015898	4
10	47.271080	4.318573	4	73.115570	9.895278	4	40.130660	3.692994	4
20	33.921360	3.935569	4	57.745400	8.052529	4	26.164720	4.306086	4
40	23.690750	3.817653	4	35.218570	4.735401	4	16.455440	4.818836	4

D		
Data Set-D		
Mean	SEM	N
100.000000	7.687875	4
57.364370	9.015898	4
40.130660	3.692994	4
26.164720	4.306086	4
16.455440	4.818836	4

able format: Grouped	A			B			C		
	Data Set-A			Data Set-B			Data Set-C		
	Mean	SEM	N	Mean	SEM	N	Mean	SEM	N
0	100.000000	5.777298	4	100.000000	6.051938	4	100.000000	6.322669	4
2.5	86.865070	7.627915	4	73.636610	9.777116	4	90.919900	9.946898	4
5	67.070630	10.705610	4	56.749090	6.516867	4	74.251330	8.167434	4
10	55.840730	11.418050	4	36.269860	8.518215	4	57.548360	6.391429	4
20	45.085430	1.537587	4	23.098660	5.179248	4	50.676320	7.267370	4

D		
Data Set-D		
Mean	SEM	N
100.000000	2.645047	4
66.010610	11.053640	4
46.457760	10.046310	4
32.469280	10.241940	4
19.264430	4.124454	4

Uncropped images for Figure 6C

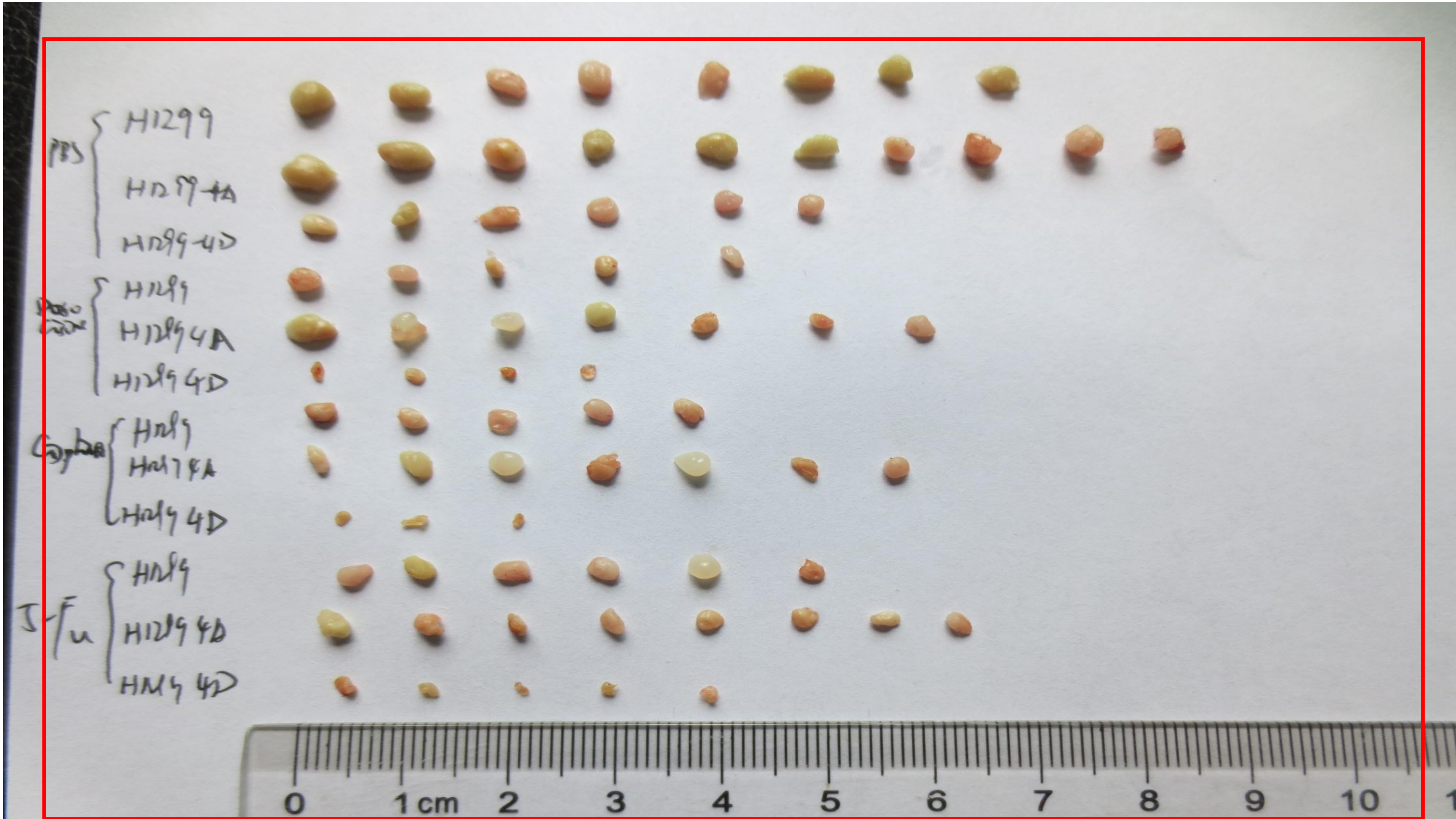

Original data for Figure 6D

	A	B	C	D	E	F	G	H	I	J
	Data Set-A	Data Set-B	Data Set-C	Data Set-D	Data Set-E	Data Set-F	Data Set-G	Data Set-H	Data Set-I	Data Set-J
	Y	Y	Y	Y	Y	Y	Y	Y	Y	Y
1	38.52576	91.32032	8.12500	7.18848	33.28000	0.52000	7.18848	6.32684	0.69212	9.13952
2	20.43808	81.88128	7.18848	5.53696	28.53344	1.42688	5.53696	18.68724	1.42688	9.13952
3	18.68724	65.00000	6.32684	4.16000	17.03936	0.89856	5.53696	22.29500	0.89856	9.13952
4	17.03936	33.28000	5.53696	4.16000	18.68724	1.42688	5.53696	20.43808		9.13952
5	14.04000	38.52576	5.53696	4.16000	14.04000		4.16000	24.26112		8.12500
6	18.68724	35.83892	4.81572		14.04000			14.04000		5.53696
7	15.49132	33.28000			17.03936			15.49132		
8	17.03936	47.38500								
9		53.98796								
10		41.34364								

K	L
Data Set-K	Data Set-L
Y	Y
20.43808	3.03264
17.03936	1.75500
14.04000	0.52000
17.03936	0.89856
15.49132	1.75500
14.04000	
14.04000	
14.04000	

Supplementary Table 1 NIP30 mutation analysis from TCGA database

Study	Protein Change	Mutation Type	Chromosome	Start Pos	End Pos	Ref	Var	Mut cases	All samples	Mutant frequency
**Kidney Chromophobe (TCGA, Provisional)**	S228L	Missense_Mutation	16	57188284	57188284	G	A	1	66	1.515%
**Lung Squamous Cell Carcinoma (TCGA, Provisional)**	R27M	Missense_Mutation	16	57207687	57207687	C	A	1	178	0.562%
**Colorectal Adenocarcinoma (TCGA, Provisional)**	P213S	Missense_Mutation	16	57188330	57188330	G	A	1	220	0.455%
**Colorectal Adenocarcinoma (TCGA, Provisional)**	R104Q	Missense_Mutation	16	57206200	57206200	C	T	1	220	0.455%
**Kidney Renal Clear Cell Carcinoma (TCGA, Provisional)**	X5_splice	Splice_Site	16	57207783	57207783	T	A	2	448	0.446%
**Uterine Corpus Endometrial Carcinoma (TCGA, Provisional)**	E111*	Nonsense_Mutation	16	57206180	57206180	C	A	1	242	0.413%
**Uterine Corpus Endometrial Carcinoma (TCGA, Provisional)**	E101K	Missense_Mutation	16	57206210	57206210	C	T	1	242	0.413%
**Uterine Corpus Endometrial Carcinoma (TCGA, Provisional)**	R24W	Missense_Mutation	16	57207697	57207697	G	A	1	242	0.413%
**Uterine Corpus Endometrial Carcinoma (TCGA, Provisional)**	Q149H	Missense_Mutation	16	57201040	57201040	C	A	1	242	0.413%
**Uterine Corpus Endometrial Carcinoma (TCGA, Provisional)**	K129R	Missense_Mutation	16	57201101	57201101	T	C	1	242	0.413%
**Skin Cutaneous Melanoma (TCGA, Provisional)**	S224F	Missense_Mutation	16	57188296	57188296	G	A	1	287	0.348%
**Skin Cutaneous Melanoma (TCGA, Provisional)**	D91del	In_Frame_Del	16	57206236	57206238	TCA	-	1	287	0.348%
**Stomach Adenocarcinoma (TCGA, Provisional)**	S196R	Missense_Mutation	16	57188381	57188381	T	G	1	287	0.348%
**Stomach Adenocarcinoma (TCGA, Provisional)**	A150V	Missense_Mutation	16	57201038	57201038	G	A	1	287	0.348%
**Liver Hepatocellular Carcinoma (TCGA, Provisional)**	D178E	Missense_Mutation	16	57197926	57197926	A	T	1	366	0.273%
**Prostate Adenocarcinoma (TCGA, Provisional)**	D83G	Missense_Mutation	16	57206263	57206263	T	C	1	492	0.203%
**Head and Neck Squamous Cell Carcinoma (TCGA, Provisional)**	K188T	Missense_Mutation	16	57188404	57188404	T	G	1	504	0.198%
**Head and Neck Squamous Cell Carcinoma (TCGA, Provisional)**	E108del	In_Frame_Del	16	57206187	57206189	TTC	-	1	504	0.198%
**Head and Neck Squamous Cell Carcinoma (TCGA, Provisional)**	E231K	Missense_Mutation	16	57188276	57188276	C	T	1	504	0.198%
**Breast Invasive Carcinoma (TCGA, Provisional)**	P198L	Missense_Mutation	16	57188374	57188374	G	A	1	963	0.104%
**Breast Invasive Carcinoma (TCGA, Provisional)**	X43_splice	Splice_Region	16	57206793	57206793	G	A	1	963	0.104%

Supplementary Table 2 NIP30 deletion analysis from TCGA database

	Deletion cases	All cases	Frequency
**Bladder Urothelial Carcinoma (TCGA, Provisional)**	13	408	3.19%
**Prostate Adenocarcinoma (TCGA, Provisional)**	14	492	2.85%
**Lymphoid Neoplasm Diffuse Large B-cell Lymphoma (TCGA, Provisional)**	1	48	2.08%
**Uveal Melanoma (TCGA, Provisional)**	1	80	1.25%
**Skin Cutaneous Melanoma (TCGA, Provisional)**	3	367	0.82%
**Thymoma (TCGA, Provisional)**	1	123	0.81%
**Stomach Adenocarcinoma (TCGA, Provisional)**	3	441	0.68%
**Esophageal Carcinoma (TCGA, Provisional)**	1	184	0.54%
**Acute Myeloid Leukemia (TCGA, Provisional)**	1	191	0.52%
**Sarcoma (TCGA, Provisional)**	1	257	0.39%
**Cervical Squamous Cell Carcinoma and Endocervical Adenocarcinoma (TCGA, Provisional)**	1	295	0.34%
**Liver Hepatocellular Carcinoma (TCGA, Provisional)**	1	370	0.27%
**Lung Adenocarcinoma (TCGA, Provisional)**	1	516	0.19%
**Head and Neck Squamous Cell Carcinoma (TCGA, Provisional)**	1	522	0.19%
**Uterine Corpus Endometrial Carcinoma (TCGA, Provisional)**	1	539	0.19%
**Glioblastoma Multiforme (TCGA, Provisional)**	1	577	0.17%
**Ovarian Serous Cystadenocarcinoma (TCGA, Provisional)**	1	579	0.17%
**Breast Invasive Carcinoma (TCGA, Provisional)**	1	1080	0.09%

Supplementary Table 3 Phosphorylated at S228 (related Supplementary Figure 3D)

$b-H_3PO_4$	b				y	y^{+2}	$y-H_3PO_4$	$y-H_3PO_4^{+2}$
---	---	1	**S**	21	---	---	---	---
---	145.0608	2	**G**	20	*1995.7663*	998.3868	*1897.7894*	949.3983
---	232.0928	3	**S**	19	*1938.7448*	969.876	*1840.7679*	920.8876
---	319.1248	4	**S**	18	*1851.7128*	926.36	*1753.7359*	877.3716
---	434.1518	5	**D**	17	*1764.6807*	882.844	*1666.7038*	833.8556
---	521.1838	6	**S**	16	*1649.6538*	825.3305	*1551.6769*	776.3421
---	650.2264	7	**E**	15	*1562.6218*	781.8145	1464.645	732.8261
---	737.2584	8	**S**	14	1433.579	717.2932	1335.602	668.3048
---	824.2904	9	**S**	13	1346.547	673.7772	1248.57	624.7888
893.3119	991.2888	10	**S(Phospho)**	12	1259.515	630.2612	1161.538	581.2727
1008.339	1106.316	11	**D**	11	1092.517	546.762	---	---
1095.371	1193.348	12	**S**	10	977.4898	489.2485	---	---
1224.414	1322.39	13	**E**	9	890.4578	445.7325	---	---
1281.435	1379.412	14	**G**	8	761.4152	381.2112	---	---
1382.483	1480.46	15	**T**	7	704.3937	352.7005	---	---
1495.567	*1593.5436*	16	**I**	6	603.3461	302.1767	---	---
1609.6096	*1707.5865*	17	**N**	5	490.262	245.6346	---	---
1680.6467	*1778.6236*	18	**A**	4	376.2191	188.6132	---	---
1781.6944	*1879.6713*	19	**T**	3	305.1819	153.0946	---	---
1838.7159	*1936.6928*	20	**G**	2	204.1343	*102.5708*	---	---
---	---	21	**K**	1	147.1128	*74.06*	---	---

Supplementary Table 4 Phosphorylated at S230 (related Supplementary Figure 3E)

$b-H_3PO_4$	b				y	y^{+2}	$y-H_3PO_4$	$y-H_3PO_4^{+2}$
---	---	1	**S**	21	---	---	---	---
---	*145.0608*	2	**G**	20	*1995.7663*	998.3868	*1897.7894*	949.3983
---	232.0928	3	**S**	19	*1938.7448*	969.876	*1840.7679*	920.8876
---	319.1248	4	**S**	18	*1851.7128*	926.36	*1753.7359*	877.3716
---	434.1518	5	**D**	17	*1764.6807*	882.844	*1666.7038*	833.8556
---	521.1838	6	**S**	16	*1649.6538*	825.3305	*1551.6769*	776.3421
---	650.2264	7	**E**	15	*1562.6218*	781.8145	*1464.6449*	732.8261
---	737.2584	8	**S**	14	*1433.5792*	717.2932	1335.602	668.3048
---	824.2904	9	**S**	13	*1346.5471*	673.7772	1248.57	624.7888
---	911.3225	10	**S**	12	1259.515	630.2612	1161.538	581.2727
---	1026.349	11	**D**	11	1172.483	586.7452	1074.506	537.7567
1095.371	1193.348	12	**S(Phospho)**	10	1057.456	529.2317	959.4793	480.2433
1224.414	1322.39	13	**E**	9	890.4578	445.7325	---	---
1281.435	*1379.4118*	14	**G**	8	761.4152	381.2112	---	---
1382.4826	*1480.4595*	15	**T**	7	704.3937	352.7005	---	---
1495.5667	*1593.5436*	16	**I**	6	603.3461	302.1767	---	---
1609.6096	*1707.5865*	17	**N**	5	490.262	245.6346	---	---
1680.6467	*1778.6236*	18	**A**	4	376.2191	188.6132	---	---
1781.6944	*1879.6713*	19	**T**	3	305.1819	*153.0946*	---	---
1838.7159	*1936.6928*	20	**G**	2	204.1343	*102.5708*	---	---
---	---	21	**K**	1	*147.1128*	*74.06*	---	---

Uncropped gel images for Supplemental Figure3H

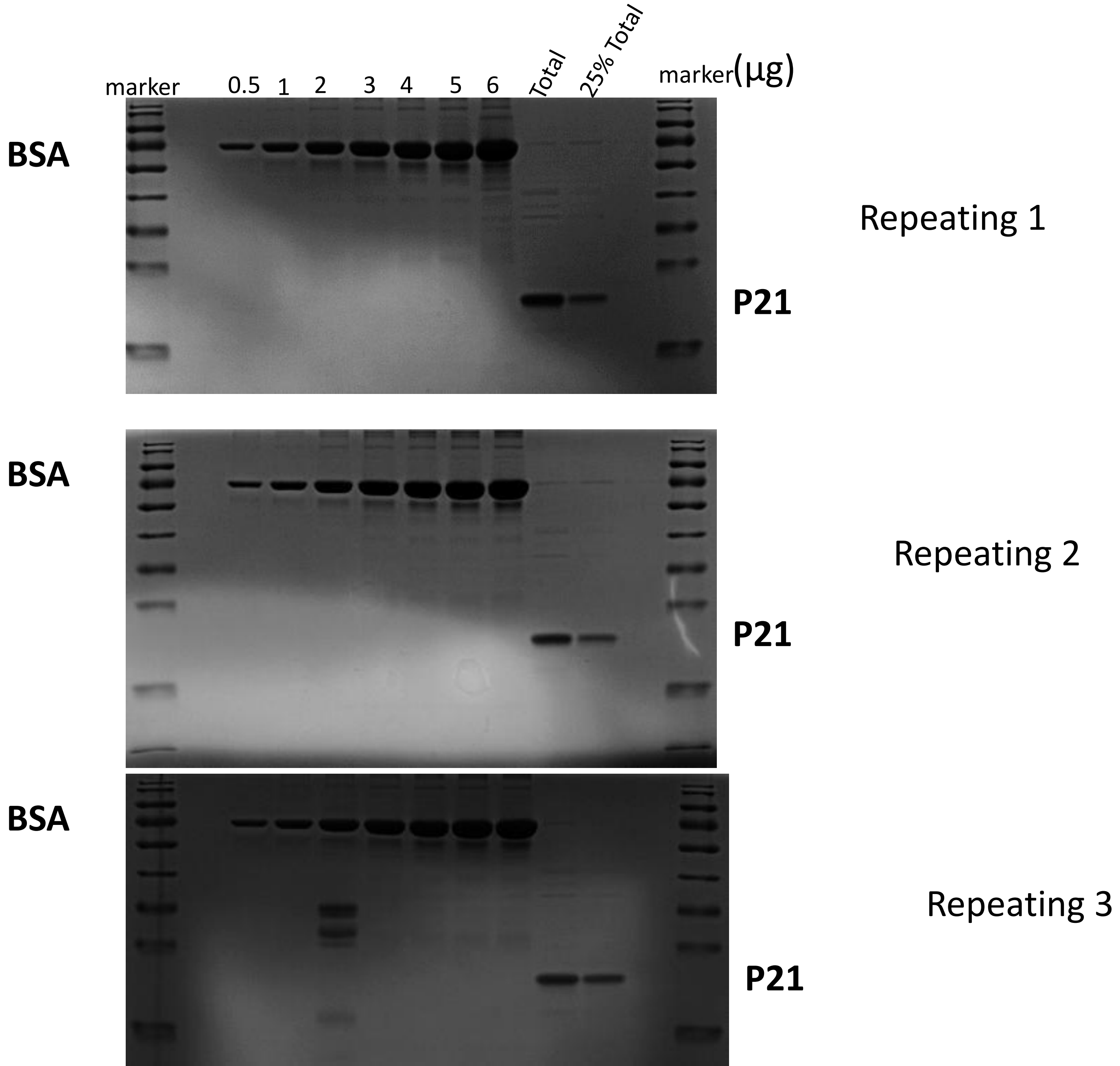

Uncropped gel images for Supplemental Figure3I

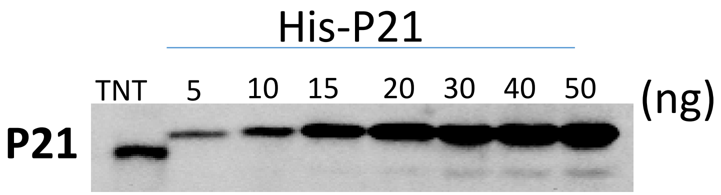

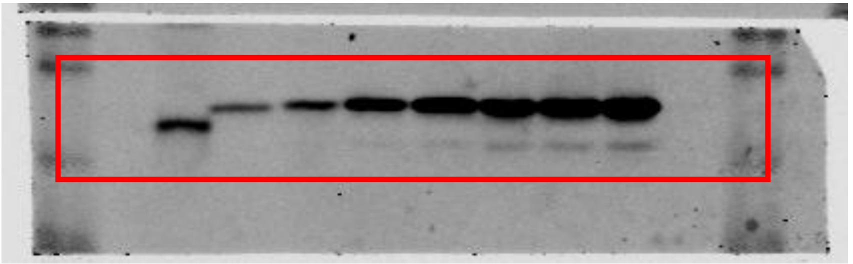

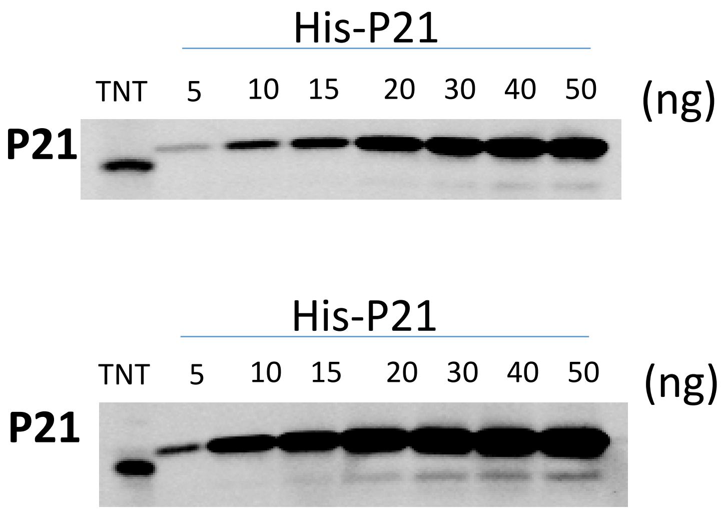

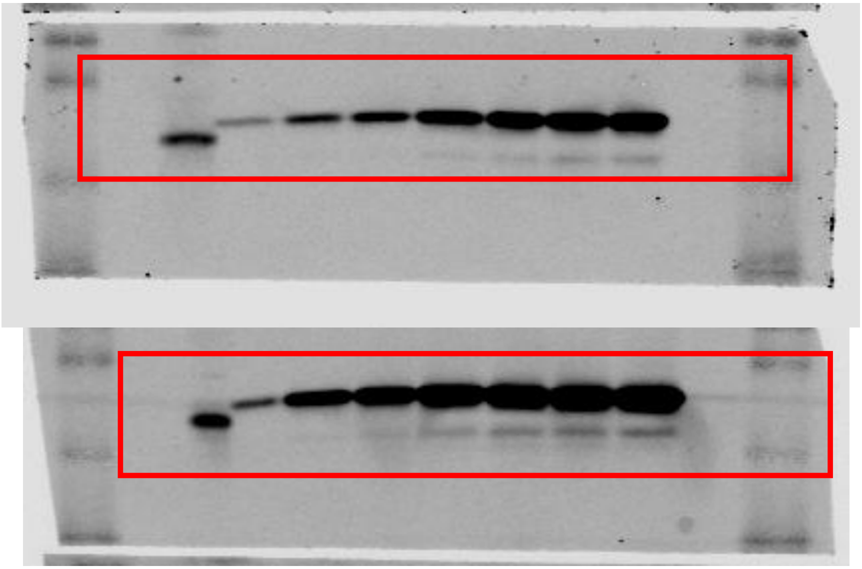

Uncropped gel images for Supplemental Figure3J-K   Repeating 1

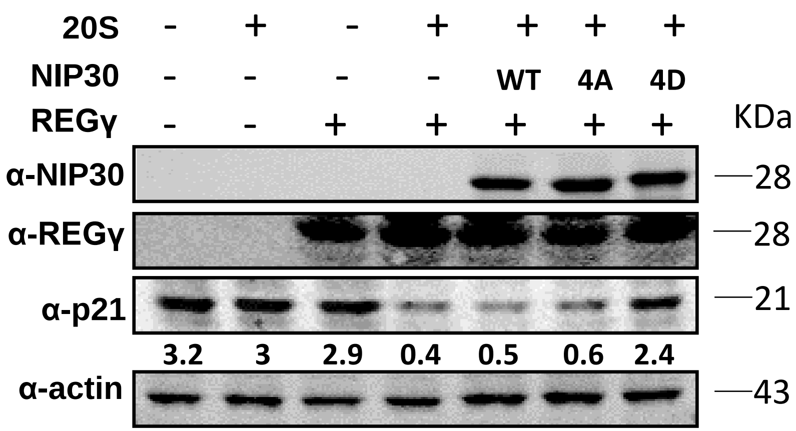

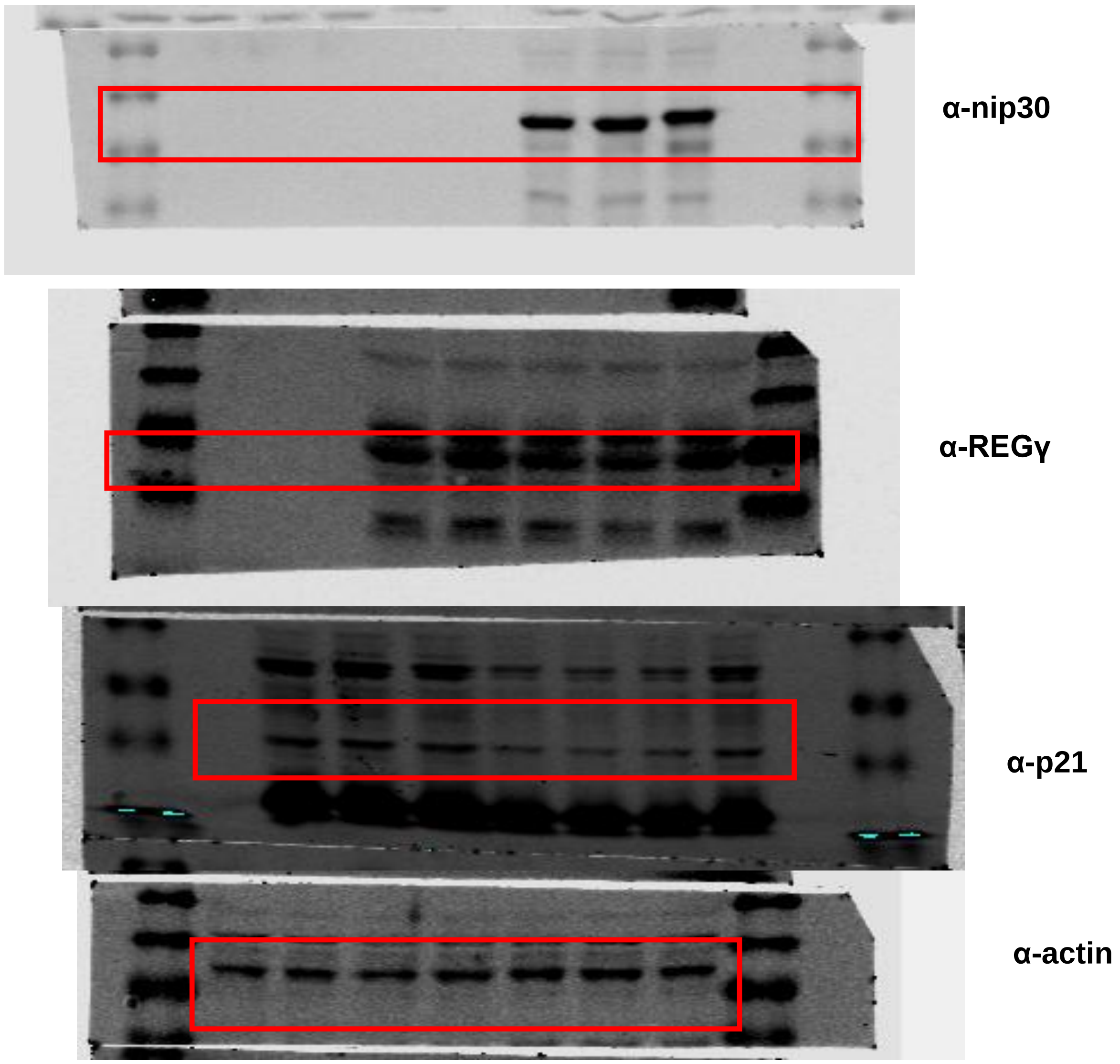

Uncropped gel images for Supplemental Figure3J-K Repeating 2

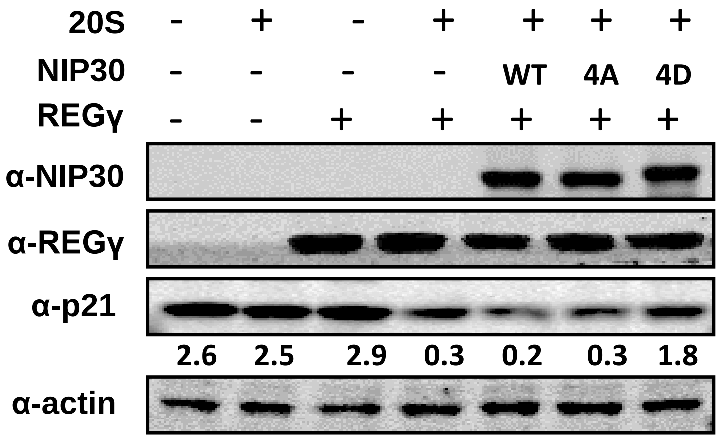

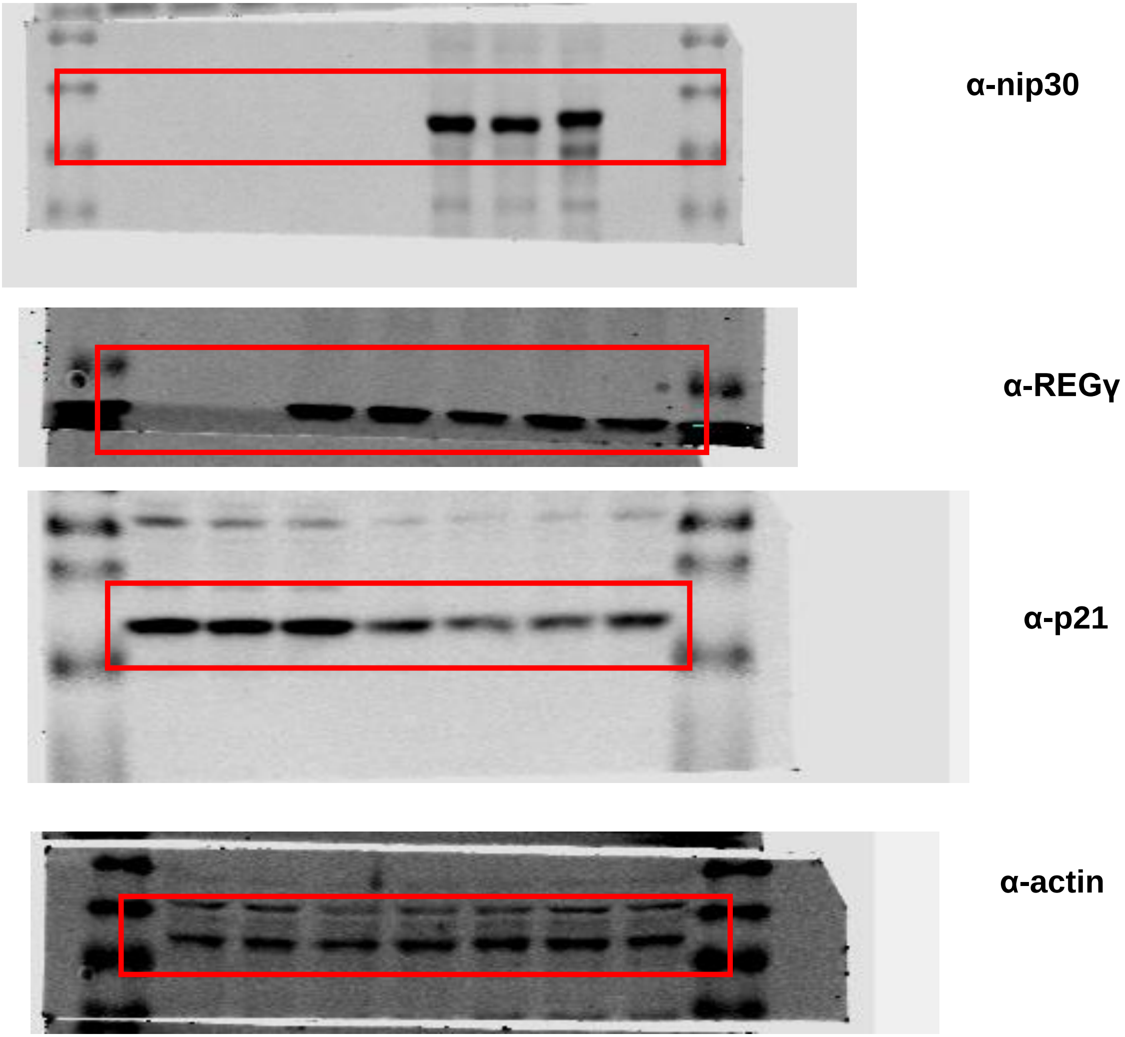

Uncropped gel images for Supplemental Figure3J-K Repeating 3

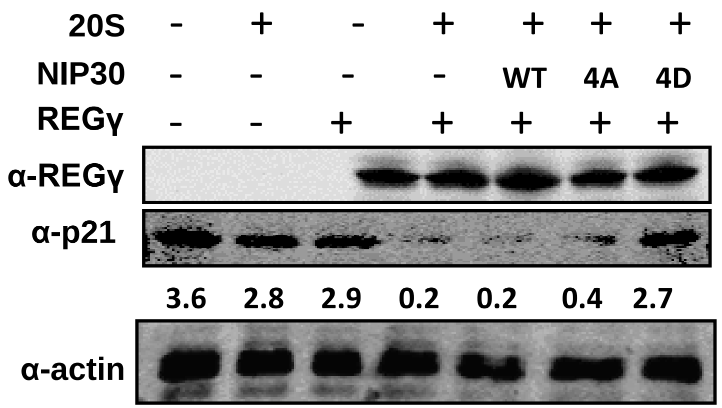

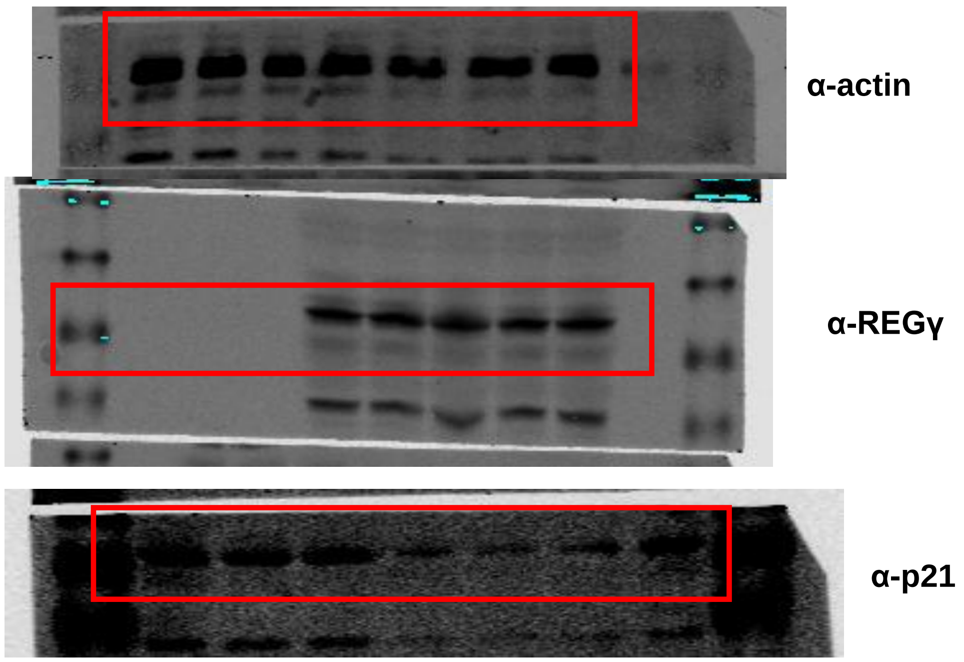

Original data for Supplemental Figure3J-K

Image J value for each lane per experiment for actin and p21
(S-fig3K-table1)

	Grayscale Value by Image J						
	Repeat1		Repeat2		Repeat3		
	actin	p21	actin	p21	actin	actin/20	p21
lane1	10570	33974	10113	26052	189740	9487	33807
lane2	10666	32192	9958	24399	178525	8926.25	25106
lane3	10787	31012	9994	28486	176434	8821.7	25561
lane4	10618	4620	11475	2946	179131	8956.55	2158
lane5	11615	5846	10331	2309	177536	8876.8	2097
lane6	11386	7178	10419	3627	183433	9171.65	3366
lane7	10082	24568	10237	18746	167203	8360.15	22468

Note.*actin/20 refers to normalization by 20 fold reduction in grayscale values for all actin levels in repeating experiment 3. We did so with the intention to reduce variations among the 3 experiments. We have carefully calculated and concluded that with or without normalization by 20 fold reductions, the final results in the table s3 are the same for the relative p21 expression levels. For example, we got the lane 1 and lane 7 values in repeat 3 as 0.178175 and 0.134376 respectively by p21/actin without normalization of 20-fold reductions. When we normalize lane 7 in repeat3 to 3.2142, we use this formula (Y=0.134376 /0.178175*3.2142=2.4241), which is the same as the calculation with normalization by 20-fold reduction of actin [p21/(actin/20)] as shown in S-fig3K-table3.

P21 value / actin value
(S-fig3K-table2)

	Repeat1	Repeat2	Repeat3
lane1	3.2142	2.5761	3.5635
lane2	3.0182	2.4502	2.8126
lane3	2.8749	2.8503	2.8975
lane4	0.4351	0.2567	0.2409
lane5	0.5033	0.2235	0.2362
lane6	0.6304	0.3481	0.367
lane7	2.4368	1.8312	2.6875

Normalized to the first P21 band value(3.2142)
of the first repeating assay
(S-fig3K-table3)

Normalized data		
Repeat1	Repeat2	Repeat3
3.2142	3.2142	3.2142
3.0182	3.057115	2.536904
2.8749	3.556319	2.613482
0.4351	0.320285	0.217287
0.5033	0.278861	0.213047
0.6304	0.434324	0.331026
2.4368	2.284788	2.424067

When normalizing the data to the 1st band in repeat2 , we use this formula: Y=N/2.5761*3.2142, （N is the value of repeat2 in S-fig3K-table2); when normalizing the data to the 1st band in repeat3, we use the following formula: Y=N/3.5635*3.2142, (N is the value of repeat3 in S-fig3K-table2).

1 .Anti-Flag-mouse MBL (anti-DDDDK-mouse) reference from antibody Datasheet

Applications

Western blotting

Positive control: Fusion protein containing the Flag Tag

Recommended dilution: 1000-5000

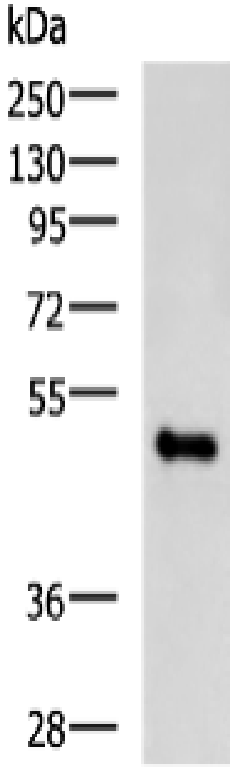

Gel: 8%SDS-PAGE

Lysate: 40 μg

Lane: Fusion protein containing the Flag Tag

Primary antibody: D190828(Flag Tag Antibody) at dilution 1/1000

Secondary antibody: Goat anti mouse IgG at 1/5000 dilution

Exposure time: 30 seconds

2.β-actin-mouse MBL reference from antibody Datasheet

Applications

Western blotting

Predicted band size:42 kDa

Positive control: Corn leaves lysate and Nicotiana tabacum leaves lysates

Recommended dilution: 500-2000

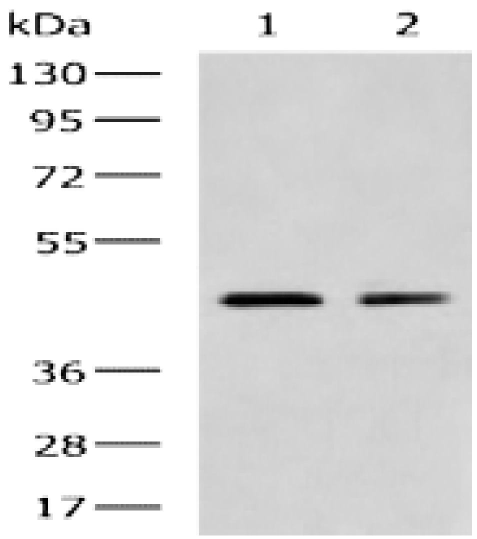

Gel: 8%SDS-PAGE

Lysate: 40 μg

Lane 1-2: Corn leaves lysate and Nicotiana tabacum leaves lysates

Primary antibody: D191048(plant-actin Antibody) at dilution 1/1000

Secondary antibody: Goat anti mouse IgG at 1/5000 dilution

Exposure time: 5 minutes

3.anti-HA-mouse reference from antibody Datasheet

Applications

Western blotting

Predicted band size:55 kDa

Positive control: Fusion proteins containing the HA Tag

Recommended dilution: 20000-100000

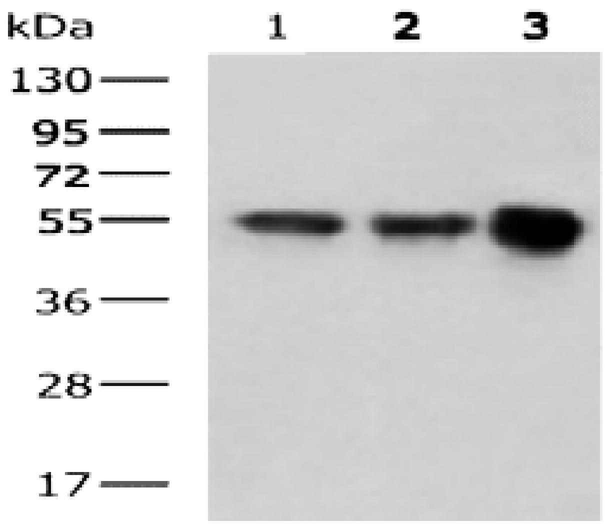

Gel: 8%SDS-PAGE

Lysate: 0.01/0.02/0.04 μg

Lane 1-3: 0.01/0.02/0.04 μg Fusion proteins containing the HA Tag

Primary antibody: D191044(HA tag Antibody) at dilution 1/50000

Secondary antibody: Goat anti mouse IgG at 1/5000 dilution

Exposure time: 30 seconds

4. anti-REGγ-rabbit(γ) reference from antibody Datasheet

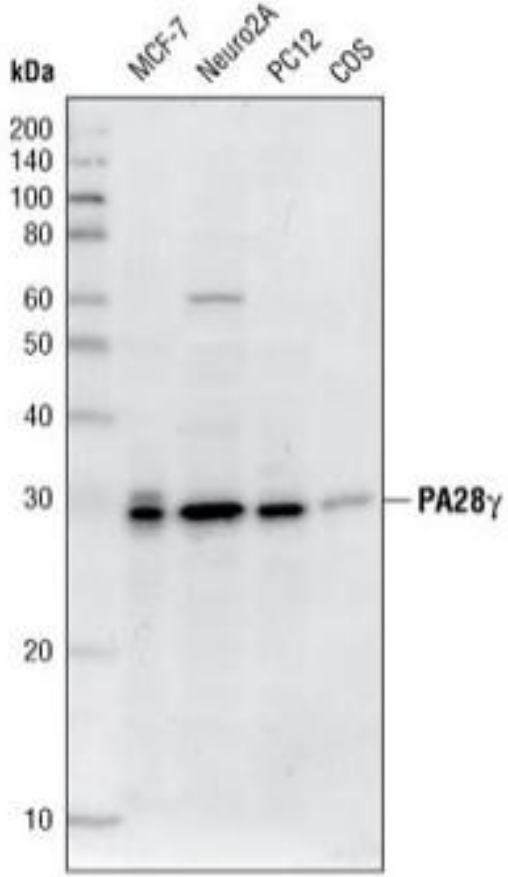

Western blot analysis of extracts from MCF-7, Neuro2A, PC12 and COS cells using PA28γ Antibody.

5. anti-NIP30-rabbit Proteintech Rabbit reference from antibody Datasheet

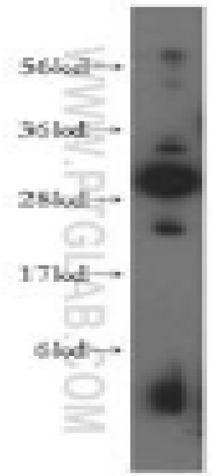

HeLa cells were subjected to SDS PAGE followed by western blot with 16830-1-AP(NIP30 antibody) at dilution of 1:500 incubated at room temperature for 1.5 hours

6. anti-p21-rabbit reference from antibody Datasheet

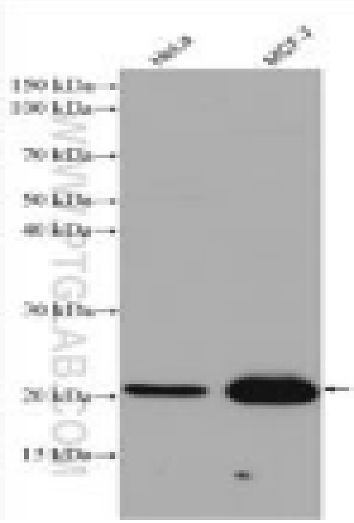

Various lysates were subjected to SDS PAGE followed by western blot with 10355-1-AP (P21 antibody) at dilution of 1:800 incubated at room temperature for 1.5 hours

7. anti-CDC25A-rabbit reference from antibody Datasheet

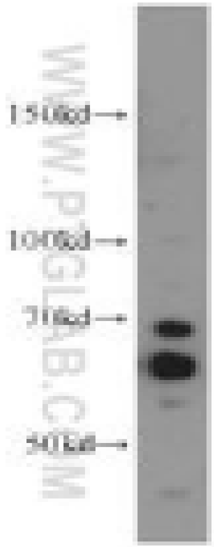

HepG2 cells were subjected to SDS PAGE followed by western blot with 55031-1-AP(CDC25A antibody) at dilution of 1:1500 incubated at room temperature for 1.5 hours

8. anti-LAST1 –rabbit reference from antibody Datasheet

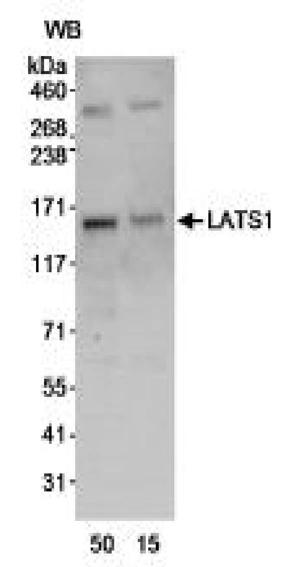

Western blot - Anti-LATS1/WARTS antibody (ab70562)

All lanes : Anti-LATS1/WARTS antibody (ab70562) at 0.4 µg/ml

Lane 1 : Hela whole cell lysate at 50 µg
Lane 2 : Hela whole cell lysate at 15 µg

Developed using the ECL technique.

Predicted band size: 127 kDa
Observed band size: 160 kDa
why is the actual band size different from the predicted?
Additional bands at: 350 kDa. We are unsure as to the identity of these extra bands.

9. pNIP30Ser228 was tested in Supplemental information 4A

A

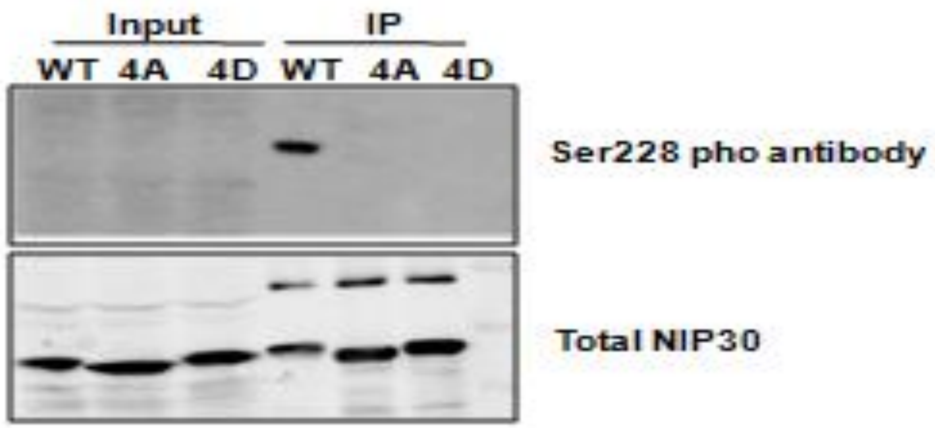

10. pNIP30Ser230 was tested in Supplemental information 4B

B

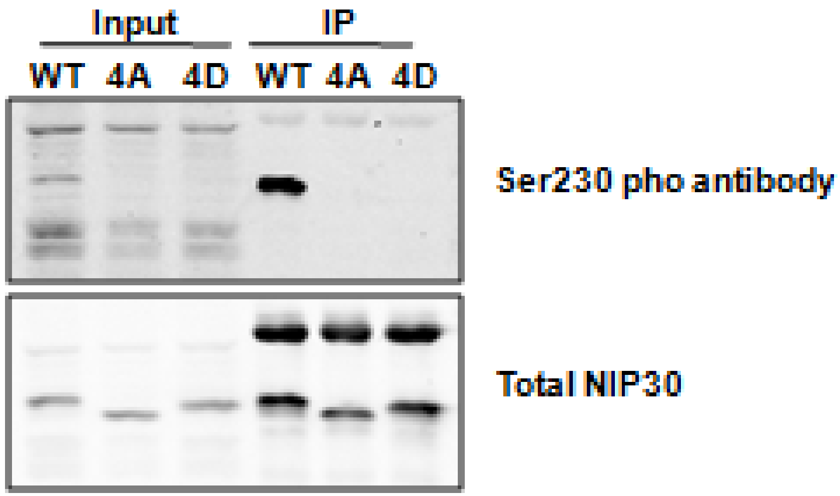

FACS gating strategy

ModFitLT software was used to analysis the FACS data.

Step 1.

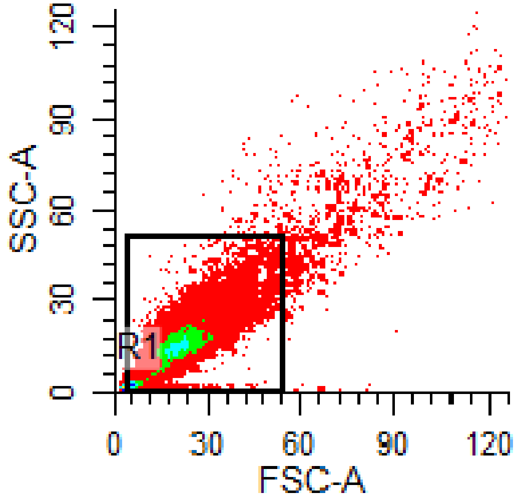

Step2.

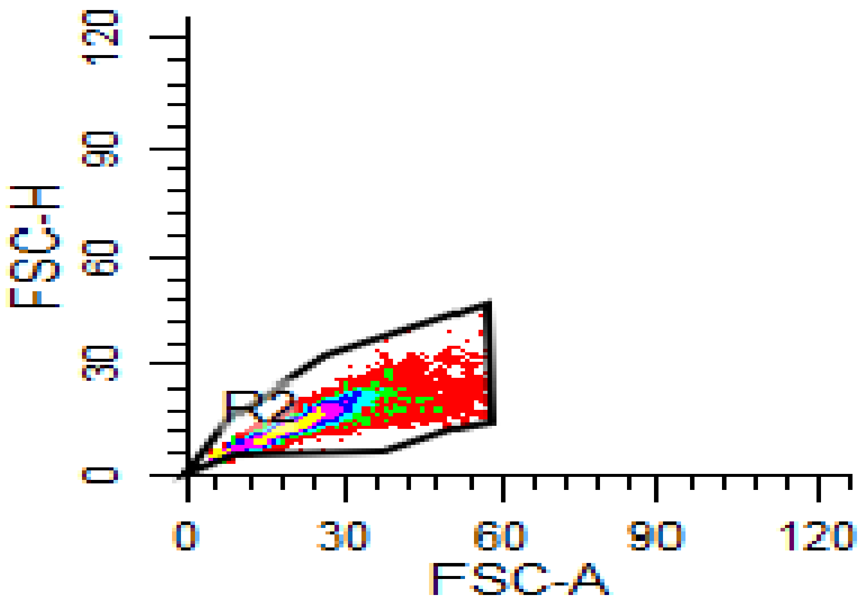

Step3.

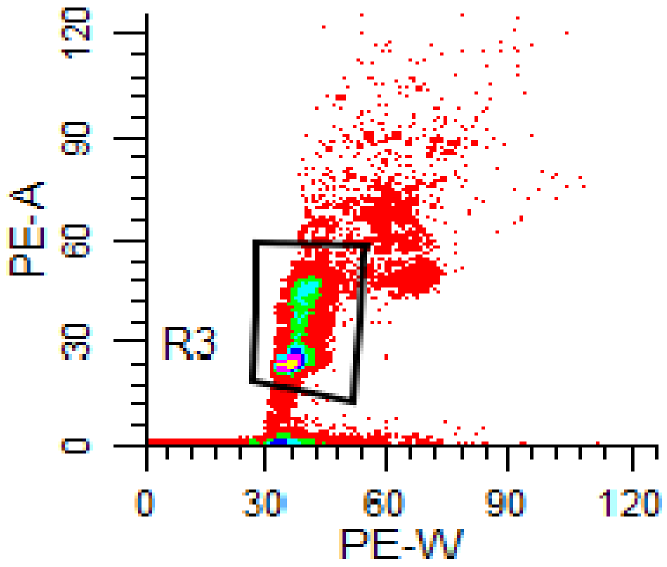

Step4.

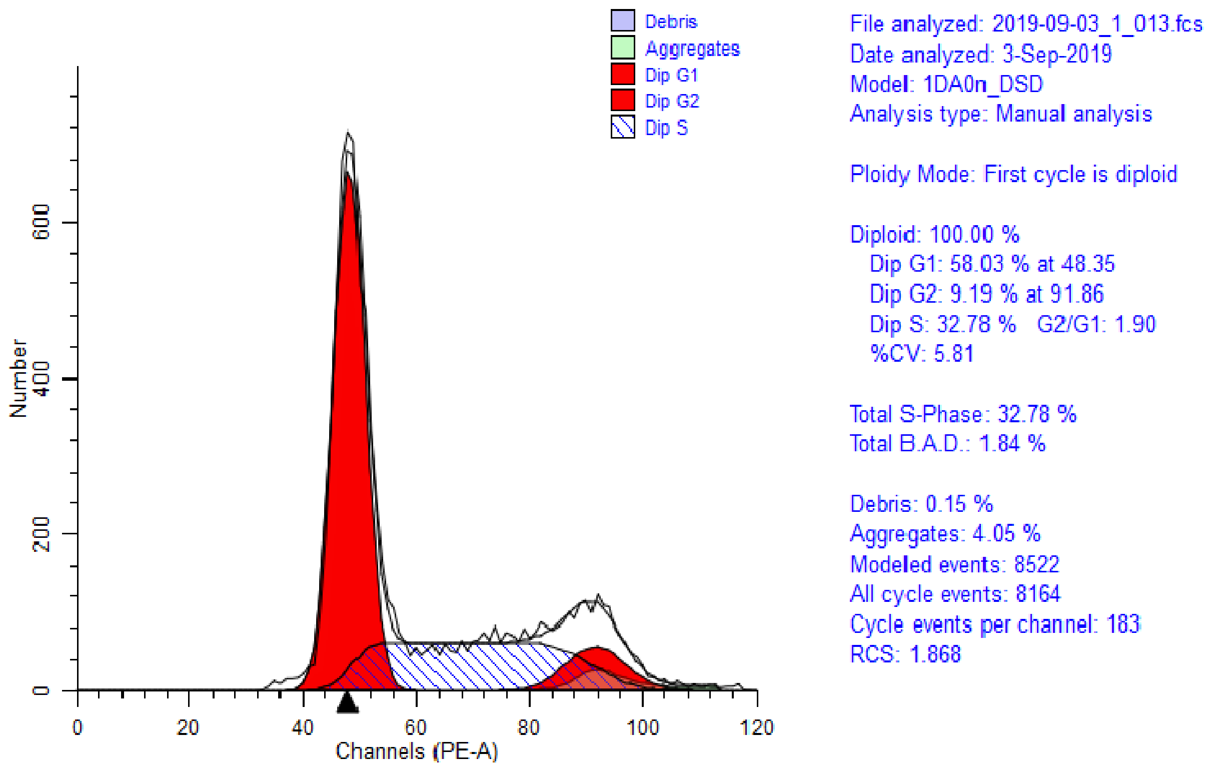
Debris
Aggregates
Dip G1
Dip G2
Dip S
Number
0
200
400
600
0
20
40
60
80
100
120
Channels (PE-A)
File analyzed: 2019-09-03_1_013.fcs
Date analyzed: 3-Sep-2019
Model: 1DA0n_DSD
Analysis type: Manual analysis
Ploidy Mode: First cycle is diploid
Diploid: 100.00 %
Dip G1: 58.03 % at 48.35
Dip G2: 9.19 % at 91.86
Dip S: 32.78 % G2/G1: 1.90
%CV: 5.81
Total S-Phase: 32.78 %
Total B.A.D.: 1.84 %
Debris: 0.15 %
Aggregates: 4.05 %
Modeled events: 8522
All cycle events: 8164
Cycle events per channel: 183
RCS: 1.868
